# Garcinuntones A‒C, three new polyprenylated xanthones with cytotoxic and anti-HIV-1 activities from *Garcinia nuntasaenii*

**DOI:** 10.1007/s11418-026-02060-3

**Published:** 2026-07-01

**Authors:** Suppisak Chaturonrutsamee, Chutima Kuhakarn, Sariyarach Thanasansurapoung, Samran Prabpai, Arthit Chairoungdua, Radeekorn Akkarawongsapat, Narong Nuntasaen, Sakchai Hongthong, Bodee Nutho, Vichai Reutrakul

**Affiliations:** 1 Department of Chemistry, Faculty of Science, Mahidol University, Rama VI Road, Bangkok, 10400 Thailand; 2 Center of Excellence for Innovation in Chemistry (PERCH-CIC), Faculty of Science, Mahidol University, Rama VI Road, Bangkok, 10400 Thailand; 3 Research and Innovation Department, International Laboratories Corp., Ltd., Bang Phli, Samut Prakan, 10540 Thailand; 4 Department of Physiology, Faculty of Science, Mahidol University, Rama VI Road, Bangkok, 10400 Thailand; 5 Department of Microbiology, Faculty of Science, Mahidol University, Rama VI Road, Bangkok, 10400 Thailand; 6 Science and Innovation for Development Program, and Division of Chemistry, Faculty of Science and Technology, Rajabhat Rajanagarindra University, Chachoengsao, 24000 Thailand; 7 Department of Pharmacology, Faculty of Science, Mahidol University, Rama VI Road, Bangkok, 10400 Thailand

**Keywords:** *Garcinia nuntasaenii*, Xanthones, Triterpenes, Cytotoxicity, Anti-HIV-1

## Abstract

Three previously undescribed polyprenylated xanthone derivatives named garcinuntones A‒C (**1** − **3**), together with fifteen known compounds (**4** − **18**) were isolated from the leaves and twigs of *Garcinia nuntasaenii* Ngerns. & Suddee (Clusiaceae). Their structures were determined by analysis of their physical and spectroscopic data and by comparison with those of related compounds reported in the literature. The absolute configuration of garcinuntone B (**2**) was established by single crystal X-ray crystallographic analysis using Cu Kα radiation data. Cytotoxic activity against several cancer cell lines (P-388, KB, HT-29, MCF-7, A549, and ASK) and anti-HIV-1 activity using syncytium inhibition and anti-HIV-1 reverse transcriptase assays of some isolated compounds were evaluated. Garcinuntone B exhibited cytotoxicity against some tested cell lines with IC_50_ values in the range of 4.82 to 6.92 μM. In anti-HIV-1 assays, garcinuntone B demonstrated selective activity against the reverse transcriptase (RT) enzyme, showing an IC_50_ of 23.61 µM, while it proved to be toxic to the host cell line in the syncytium inhibition assay. In addition, the binding affinity of garcinuntone B to HIV-1 RT was assessed through molecular docking study. Like other non-nucleoside RT inhibitors, garcinuntone B showed stable number of contacts with the binding residues in the binding pocket of HIV-1 RT enzyme.

**Graphical abstract:**

**Figure:**
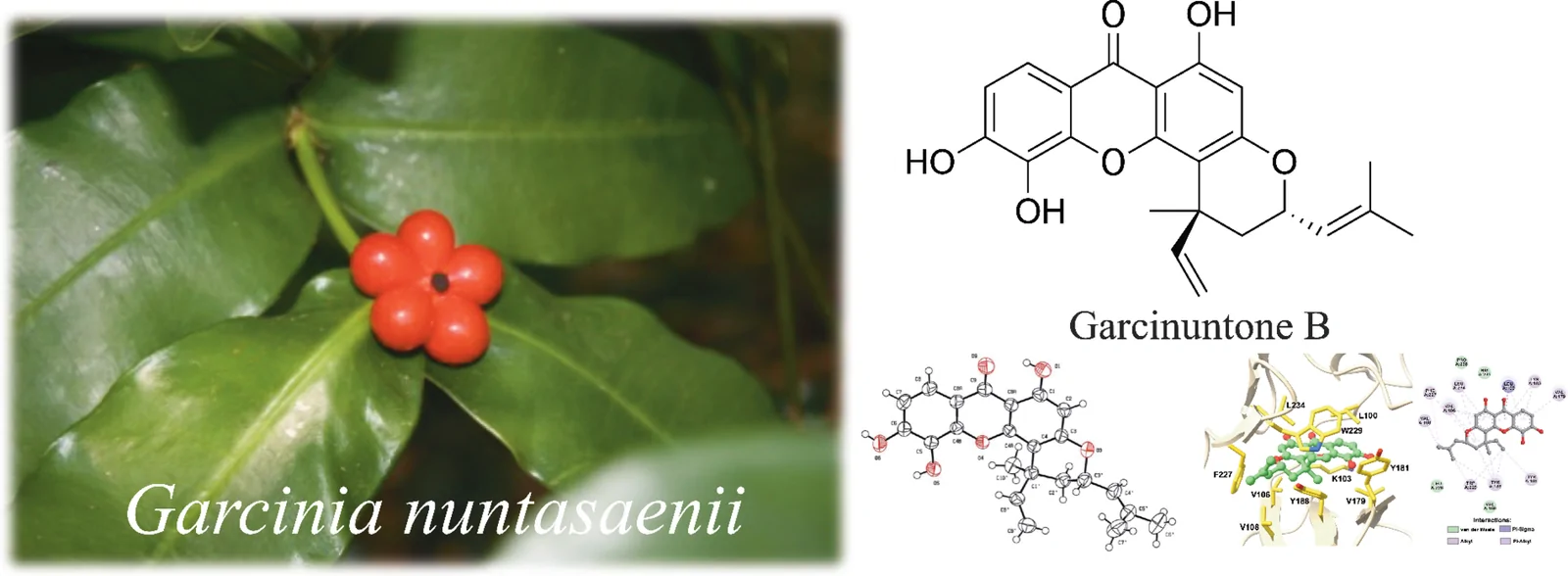

**Supplementary Information:**

The online version contains supplementary material available at https://doi.org/10.1007/s11418-026-02060-3.

## Introduction

The genus *Garcinia* comprises more than 300 species and belongs to the family Clusiaceae [1–3]. This genus is a rich source of secondary metabolites with diverse bioactivities such as anti-inflammatory [4], anticancer [5], anti-oxidant [6], and antibacterial activities [7]. Previous reports on phytochemical investigations of plants in the *Garcinia* genus revealed that they produce a large number of biologically active substances such as benzophenones [8], biflavonoids [9], biphenyls [10], flavonoids [11], triterpenes [12], and xanthones [5].

*Garcinia nuntasaenii* Ngerns. & Suddee (Clusiaceae) called in Thai “Chang-nga-ek” grows in the open-dry evergreen forest of the Northeastern part of Thailand [13]. The roots of *G. nuntasaenii* were used to treat muscle soreness in traditional Thai medicine. In our continuing search for new biologically active compounds from plants in the *Garcinia* genus [8, 12], earlier, we have reported the isolation of polycyclic polyprenylated acylphloroglucinols (PPAPs), biphenyls, flavonoids, biflavonoids, lanostane triterpenoids, and xanthones from the hexanes, ethyl acetate and methanol extracts of the roots of *G. nuntasaenii* [14]. In this study, we isolated three previously undescribed constituents from the leaves and twigs of *G. nuntasaenii* and the procedures used to determine the chemical structures are described below. Some of the isolated compounds were evaluated for their cytotoxicity against various cancerous cell lines and anti-HIV-1 effects using syncytium inhibition and reverse transcriptase assays.

## Results and discussion

### Isolation and structure determination

**Fig. 1:**
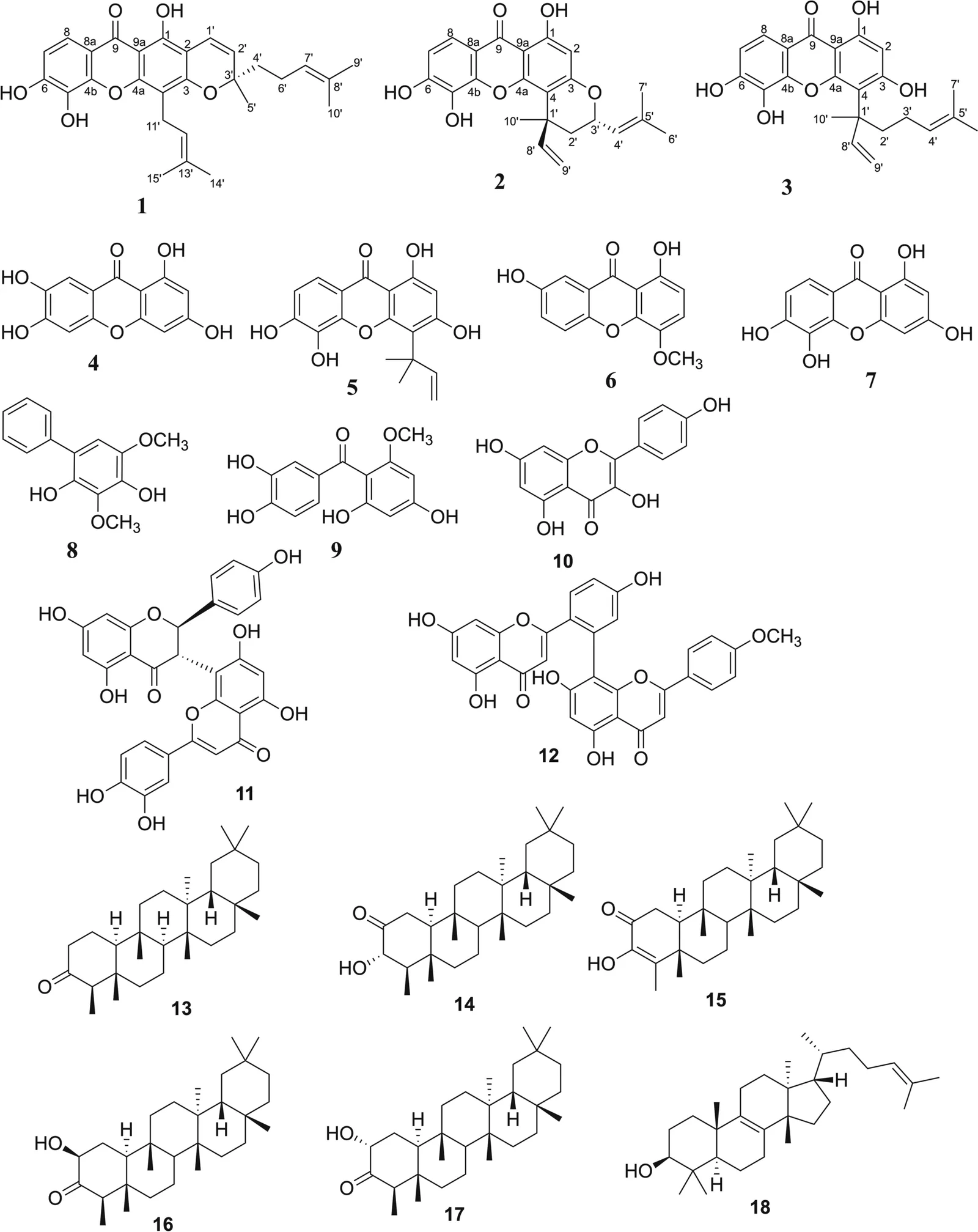
Structures of compounds **1**‒**18** isolated from the leaves and twigs of *Garcinia nuntasaenii*

Primarily, cytotoxicity and anti-HIV-1 activity of the crude hexanes, EtOAc, and MeOH extracts of leaves and twigs of *G. nuntasaenii* were screened (Tables S1 and S2). The results indicated that almost all of the extracts showed interesting anti-HIV-1 activity and having negligible cytotoxic effect, highlighting that these extracts are promising for further investigation. Herein, bioassay-guided purification of hexanes, EtOAc, and MeOH extracts of leaves and twigs of *G. nuntasaenii* led to the isolation of three previously undescribed polyprenylated xanthones, garcinuntones A‒C (**1**‒**3**) as well as fifteen known compounds (Fig. 1). The known compounds were identified as 1,3,6,7-tetrahydroxyxanthone (**4**) [15], assiguxanthone A (**5**) [16], 1,7-dihydroxy-4-methoxyxanthone (**6**) [17], 1,3,5,6-tetrahydroxyxanthone (**7**) [18], garcinuntabiphenyl A (**8**) [14], 4,6,3',4'-tetrahydroxy-2-methoxybenzophenone (**9**) [8], kaempferol (**10**) [19], morelloflavone (**11**) [20], podocarpusflavone A (**12**) [21], friedelin (**13**) [22], 3*α*-hydroxyfriedelan-2-one (**14**) [23], 3-hydroxyfriedel-3-en-2-one (**15**) [23], 2*β*-hydroxyfriedelan-3-one (**16**) [24], cerin (**17**) [25], and euphol (**18**) [26]. The structures of all isolated compounds were depicted as shown in Fig. 1. The data obtained for the known compounds were compared with published values previously reported in the literature.

Compounds **1** − **3** exhibited similar UV spectra with approximate absorption bands at 235, 285, and 330 nm typical for xanthone chromophore [8]. The FT-IR spectra of **1** − **3** showed the similarity of major absorption bands at ν_max_ 3215 − 3656 cm^−1^ (O − H stretching), 3077 − 3050 cm^−1^ (aromatic and olefinic C − H stretching), 1622 − 1657 cm^−1^ (conjugated C = O stretching), and 1580 − 1585 and 1454 − 1475 cm^−1^ (aromatic C = C stretching). Additionally, the ^1^H and ^13^C NMR spectra consist of aromatic and carbonyl resonances. The key differences among the three compounds were the location of chromane and chromene moieties on the xanthone core structure.

**Fig. 2:**
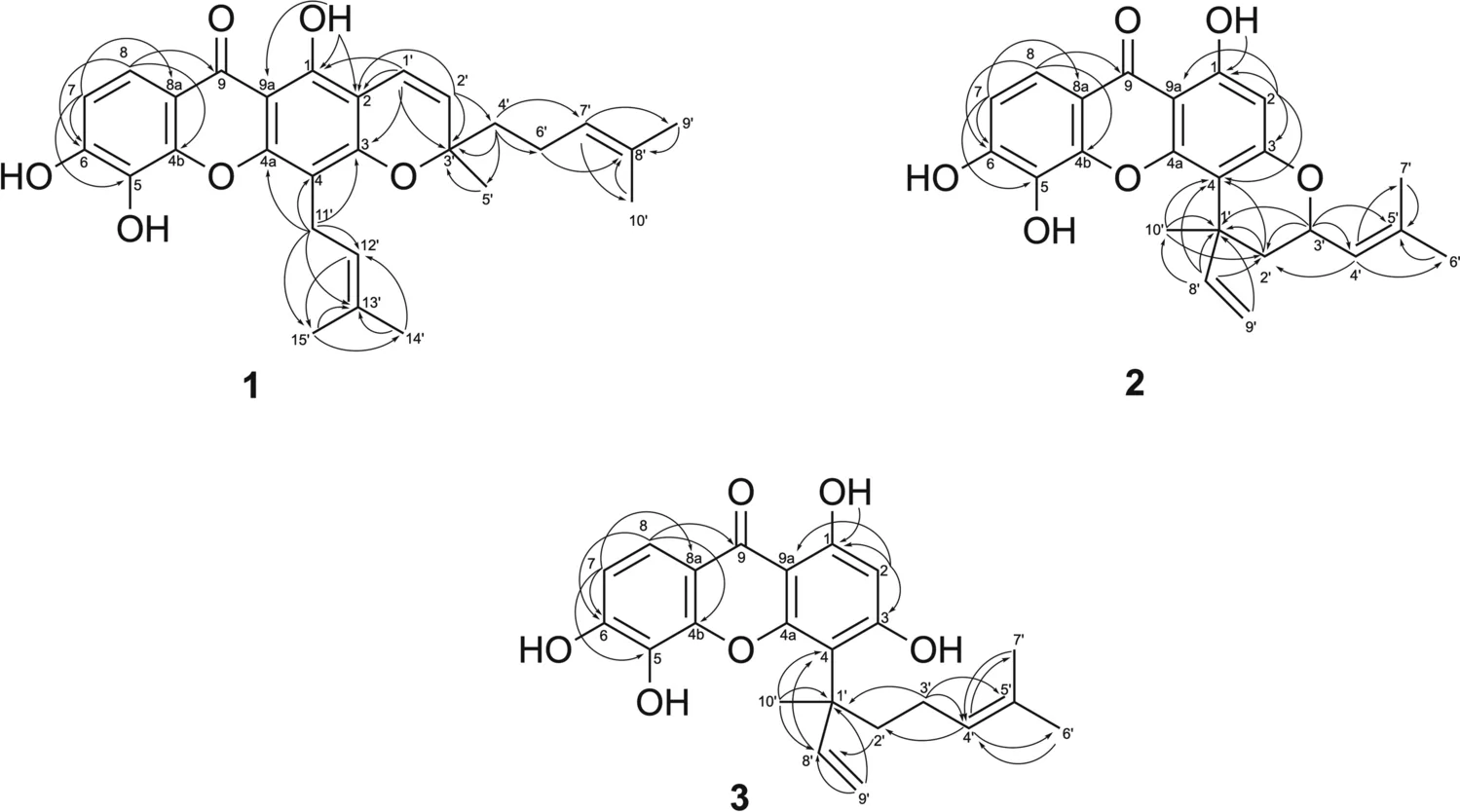
Key HMBC correlations of compounds **1** − **3**

**Fig. 3:**
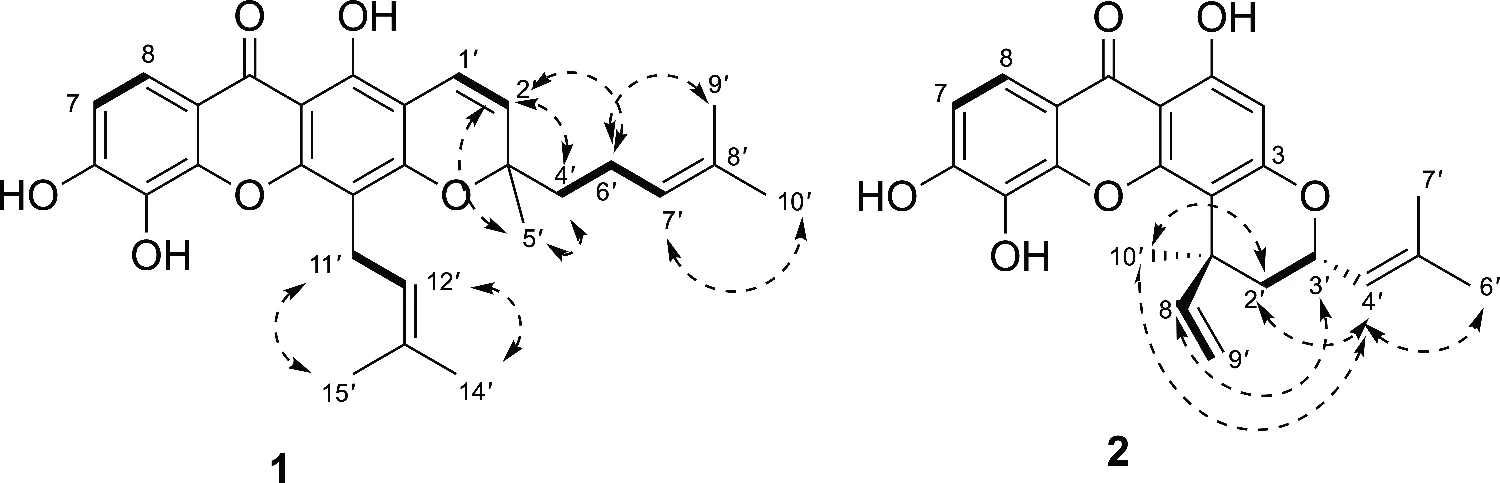
Key COSY (bolded line) and NOESY (arrowed line) correlations of compounds **1** and **2**

Compound **1** was analyzed for molecular formula C_28_H_30_O_6_ from its HRESI-MS at *m*/*z* 485.1933 [M + Na]^+^ (Calcd for C_28_H_30_O_6_Na: 485.1935). The ^1^H and ^13^C NMR data of **1** in acetone-*d*_6_ (Table 1) were similar to those of pruniflorone G [27]. The structural difference was the replacement of the isoprenyl group located at C-4 of the xanthone core present in pruniflorone G for signals of a prenyl group at *δ*_H_ 3.49 (2H, d, *J* = 7.3 Hz, H-11′), *δ*_C_ 21.9 (C-11*'*); *δ*_H_ 5.22 (1H, t, *J* = 7.3 Hz, H-12′), *δ*_C_ 123.5 (C-12*'*); *δ*_H_ 1.57 (3H, s, H-14′), *δ*_C_ 25.9 (C-14*'*); *δ*_H_ 1.78 (3H, s, H-15′), *δ*_C_ 18.1 (C-15*'*) and *δ*_C_ 131.6 (C-13′). The location of prenyl group at C-4 in compound **1** was further confirmed by HMBC correlations from the methylene proton of H-11*'* to C-3 (*δ*_c_ 159.0), C-4 (*δ*_c_ 108.0), C-4a (*δ*_c_ 155.1), C-12*'* (*δ*_c_ 123.5), C-13*'* (*δ*_c_ 131.6), and C-15*'* (*δ*_c_ 18.1) (Fig. 2). Interpretation of coupling constant and the ^1^H-^1^H COSY data for the aromatic protons at *δ*_H_ 6.92 (1H, d, *J* = 8.7 Hz, H-7) and 7.56 (1H, d, *J* = 8.7 Hz, H-8) indicated that these two protons are *ortho* to each other on an aromatic ring (Fig. 3). From HMBC data, the cross peaks between the aromatic protons H-7 (*δ*_H_ 6.92) to the aromatic carbons C-5 (*δ*_C_ 133.5) and C-8a (*δ*_C_ 114.7) as well as from H-8 (*δ*_H_ 7.56) to the aromatic carbon C-6 (*δ*_C_ 152.5) and the carbonyl carbon C-9 (*δ*_C_ 181.6) supported the locations of H-7 and H-8 as shown.

**Table 1:** ^1^H and ^13^C NMR data of **1**‒**3**

Position	**1** (acetone-*d*_6_)		**2** (pyridine-*d*_5_)		**2** (acetone-*d*_*6*_)		**3** (pyridine-*d*_5_)	
	*δ*_H_	*δ*_C_	*δ*_H_	*δ*_C_	*δ*_H_	*δ*_C_	*δ*_H_	*δ*_C_
1		156.8		162.2		162.5		162.4
2		104.7	6.67 (1H, s)	99.5	6.17 (1H, s)	99.7	6.76 (1H, s)	99.7
3		159.0		162.9		163.6		165.6
4		108.0		106.7		107.0		111.5
4a		155.1		156.9		157.2		157.3
4b		147.3		148.0		147.2		147.7
5		133.5		135.1		134.1		134.9
6		152.5		153.8		151.8		153.4
7	6.92 (1H, d, *J* = 8.7)	113.6	7.25 (1H, d, *J* = 8.7)	113.9	7.01 (1H, d, *J* = 8.7)	113.7	7.28 (1H, d, *J* = 8.6)	113.6
8	7.56 (1H, d, *J* = 8.7)	117.5	7.96 (1H, d, *J* = 8.7)	116.6	7.61 (1H, d, *J* = 8.7)	117.1	8.03 (1H, d, *J* = 8.6)	116.4
8a		114.7		114.6		115.1		114.2
9		181.6		181.6		181.7		181.9
9a		103.2		104.3		104.4		103.3
1′	6.67 (1H, d, *J* = 10.1)	116.7		38.7		39.2		45.4
2′	5.61 (1H, d, *J* = 10.1)	127.0	1.85 (1H, dd, *J* = 14.0, 1.9)	43.5	1.86 (1H, d,* J* = 4.0)	43.9	2.43‒2.53 (1H, m)	41.3
			1.92 (1H, dd, *J* = 14.0, 11.0)				2.97 (1H, dt, *J* = 12.5, 3.7)	
3′		81.4	4.96 (1H, ddd, *J* = 11.0, 8.7, 1.9)	71.0	4.8 (1H, dt, *J* = 8.3, 4.0)	71.4	2.31‒2.41 (1H, m)	24.9
							2.43‒2.53 (1H, m)	
4′	1.64‒1.70 (1H, m)	42.5	5.43 (1H, d, *J* = 8.7)	124.6	5.35 (1H, dt, *J* = 8.3, 1.4)	124.8	5.37‒5.41 (1H, m)	126.0
	1.71‒1.77 (1H, m)							
5′	1.40 (3H, s)	27.5		137.2		137.8		130.6
6′	2.03‒2.09 (2H, m)	23.6	1.70 (3H, s)	25.7	1.79 (3H, s)	25.8	1.61 (3H, s)	25.7
7′	5.05 (1H, br t, *J* = 7.2)	124.8	1.62 (3H, s)	18.3	1.70 (3H, s)	18.5	1.56 (3H, s)	17.5
8′		132.1	6.33 (1H, dd, *J* = 17.2, 10.4)	145.5	6.18 (1H, dd, *J* = 17.1, 10.5)	146.0	7.16 (1H, dd, *J* = 17.5, 10.7)	151.2
9′	1.55 (3H, s)	25.7	*E*) 5.13 (1H, dd, *J* = 17.2, 1.0)	113.7	*E*) 4.86 (1H, dd, *J* = 17.1, 1.2)	114.0	*E*) 5.43 (1H, dd, *J* = 17.5, 1.4)	108.0
			*Z*) 5.22 (1H, dd, *J* = 10.4, 1.0)		*Z*) 5.09 (1H, dd, *J* = 10.5, 1.2)		*Z*) 5.25 (1H, dd, *J* = 10.7, 1.4)	
10′	1.48 (3H, s)	17.7	2.12 (3H, s)	27.1	1.83 (3H, s)	27.2	2.35 (3H, s)	28.2
11′	3.49 (2H, d, *J* = 7.3)	21.9						
12′	5.22 (1H, t, *J* = 7.3)	123.5						
13′		131.6						
14′	1.57 (3H, s)	25.9						
15′	1.78 (3H, s)	18.1						
1-OH	13.48 (1H, s)		14.04 (1H, s)		13.21 (1H, s)		14.43 (1H, s)	
5-OH					9.72 (1H, br s)			
6-OH					8.03 (1H, br s)

Prenylated pyranoxanthones have been previously reported in natural sources, particularly in plants of the *Garcinia* genus, and their optical rotations (OR) have been documented [27, 28]. The absolute configuration at C-3′ of **1** was assigned by comparison of its OR and electronic circular dichroism (ECD) data with those of related compounds reported in the literature. By comparison the core structural framework, compound **1** and ( +)-clusifoliol [29] share a 4-methyl-3-pentenyl and a methyl substitution with a stereoginic center at C-3′ position of chromene skeleton. A comparison the OR of **1** ($\left[ {\upalpha } \right]_{{\mathrm{D}}}^{{{27}}}$ +53.8 (*c* 1.0, MeOH) with that of ( +)-clusifoliol ($\left[ {\upalpha } \right]_{{\mathrm{D}}}^{{}}$ +160.0 (*c* 0.01, EtOH)) revealed that the OR values of the two compounds were of the same sign. The absolute configuration of ( +)-clusifoliol was established by its chemical synthesis through Pd-catalyzed asymmetric allylic alkylation and by comparison of chiral HPLC data (retention time of chromatogram) with that of an authentic sample [30]. Based on the spectroscopic data described above, the absolute configuration at C-3′ of **1** was tentatively assigned as *S-*configuration. Furthermore, the absolute configuration of **1** was established by comparison of its ECD data with those of related compounds. A positive Cotton effect (CE) at 277 and 294 nm found in **1** was in good agreement with those of daurichromenic acid (positive CE at 262 nm) [31]; anthopogochromenic acid (positive CE at 290 nm) [32]; and ( +)-daurichromene F (positive CE at 280 nm) [33]. These data suggest that the *M*-helicity of the chromene ring system produces a positive CE in the 270–290 nm region [32, 33]. Thus, the absolute configuration of **1** was finally determined to be *S*-configuration and the structure of compound **1**, named garcinuntone A, was elucidated as shown.

**Fig. 4:**
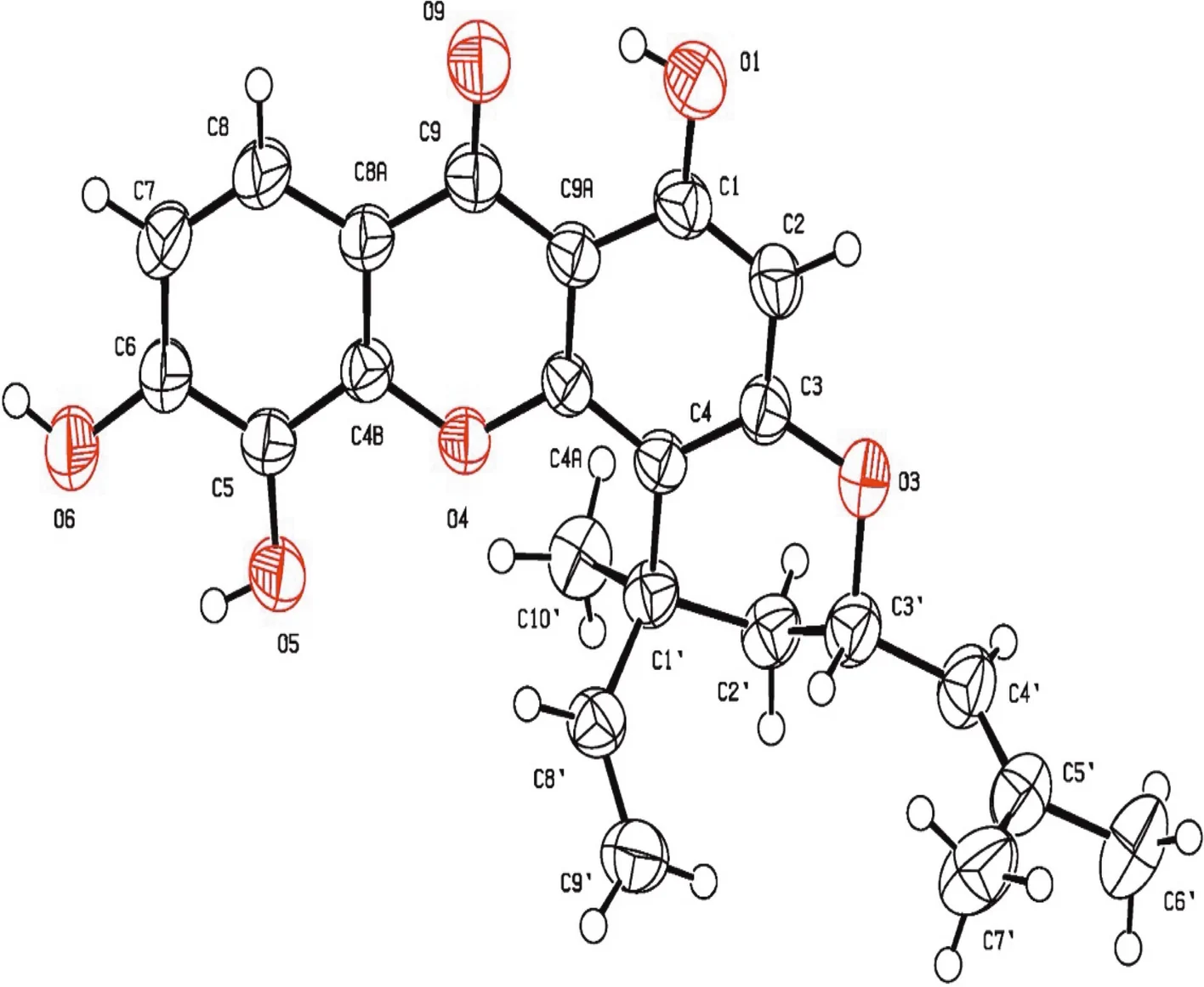
ORTEP view by the X-ray diffraction analysis of compound **2**

Compound **2** was analyzed for molecular formula C_23_H_22_O_6_ from its HRESI-MS at *m*/*z* 417.1313 [M + Na]^+^ (Calcd for C_23_H_22_O_6_Na: 417.1309). The ^1^H and ^13^C NMR spectroscopic data of **2** were acquired in pyridine-*d*_5_ and acetone-*d*_6_ (Table 1). Analysis of ^1^H and ^13^C NMR data of **2** in pyridine-*d*_5_ (Table 1) revealed resonance characteristics similar to those of hypxanthone B [34]. The key differences include (1) the absence of isopropenyl group at C-2*'* present in hypxanthone B, and (2) the presence of an additional sp^3^ quarternary carbon substituted with a methyl group and a vinyl group in compound **2**. The methyl group and the vinyl group were assigned to locate at C-1*'* by key HMBC data (Fig. 2) in which the methine proton H-8*'* (*δ*_H_ 6.33) correlated with C-4 (*δ*_C_ 106.7), C-1′ (*δ*_C_ 38.7), C-2*'* (*δ*_C_ 43.5), and C-10′ (*δ*_C_ 27.1); olefinic protons H-9*'* (*δ*_H_ 5.13 and 5.22) correlated with C-1′ (*δ*_C_ 38.7); and methyl protons H-10*'* (*δ*_H_ 2.12) correlated with C-4 (*δ*_C_ 106.7), C-1′ (*δ*_C_ 38.7), and C-2*'* (*δ*_C_ 43.5). The relative configuration of **2** was also further confirmed by NOESY experiment (Fig. 3). In NOESY spectrum, the correlations between CH_3_-10*'* and H-4*'* suggested that these groups oriented on the same face (*syn* relationship). In addition, H-3*'* showed the NOESY correlations with H-8*'* supporting that they located in the same face. This allowed the assignment of the relative configuration at C-1*'* and C-3*'* of **2** as depicted in Fig. 1. Finally, the chemical structure and the absolute configuration of **2** were also validated by single crystal X-ray diffraction technique by using Cu Kα radiation as shown in Fig. 4 (Crystallographic data have been deposited at the Cambridge Crystallographic Data Centre under the reference number CCDC 2486312). Thus, the structure of compound **2**, namely garcinuntone B, was established as shown in Fig. 1.

Compound **3** was analyzed for molecular formula C_23_H_24_O_6_ from its HRESI-MS at *m*/*z* 397.1643 [M + H]^+^ (Calcd for C_23_H_25_O_6_: 397.1646). The ^1^H and ^13^C NMR data of **3** in pyridine-*d*_5_ (Table 1), in particular, the aromatic region and a hydrogen-bonded phenolic proton were similar to those of garcinuntone B (**2**). The major difference between compounds **2** and **3** was the presence of allylic methylene resonances at *δ*_H_ 2.43 − 2.53 (1H, m, Ha-3′) and 2.31 − 2.41 (1H, m, Hb-3′) connected to C-3′ (*δ*_C_ 24.9) found in **3** instead of an oxygenated allylic methine resonance at *δ*_H_ 4.96 (1H, ddd, *J* = 11.0, 8.7, 1.9, H-3′) connected to C-3′ (*δ*_C_ 71.0) found in compound **2**. The ^1^H and ^13^C NMR spectroscopic data of **3** revealed the presence of a 3,7-dimethylocta-1,6-dien-3-yl side chain and the location of this group was confirmed using HMBC data; H-2*'* (*δ*_H_ 2.97 and 2.43‒2.53) correlated with C-8*'* (*δ*_C_ 151.2); H-3*'* (*δ*_H_ 2.43‒2.53 and 2.31‒2.41) correlated with and C-1*'* (*δ*_C_ 45.4) and C-5*'* (*δ*_C_ 130.6); H-4*'* (*δ*_H_ 5.37‒5.41) correlated with C-6*'* (*δ*_C_ 25.7), and C-2*'* (*δ*_C_ 41.3); H-8*'* (*δ*_H_ 7.16) correlated with C-4 (*δ*_C_ 111.5); H-10*'* (*δ*_H_ 2.35) correlated with C-1*'* (*δ*_C_ 45.4) and C-8*'* (*δ*_C_ 151.2) (Fig. 2). In addition, the key HMBC correlations from H-8′ (*δ*_H_ 7.16) and H-10′ (*δ*_H_ 2.35) to C-4 (*δ*_C_ 111.5) supported that the 3,7-dimethylocta-1,6-dien-3-yl moiety located at C-4 of the xanthone skeleton. Finally, the absolute configuration at C-1′ of compound **3** was undetermined due to the absence of conclusive stereochemical evidence. Additionally, stereochemistry at the identical carbon of the previously published xanthone analogs bearing the 3,7-dimethylocta-1,6-dien-3-yl moiety also remain unassigned [34]. On the basis of the aforementioned data, the structure of **3**, namely garcinuntone C, was elucidated to be as shown in Fig. 1.

### Biological activities

Some isolated compounds, including **1**‒**3**, **7**‒**9**, **12**‒**14**, and **16**‒**17**, were examined for their cytotoxic activity against a panel of mammalian cell lines including murine lymphocytic leukemia (P-388), human nasopharyngeal carcinoma (KB), human colorectal adenocarcinoma (HT-29), human breast cancer (MCF-7), human lung carcinoma (A549), rat glioma (ASK) as well as noncancerous human embryonic kidney cells (HEK-293) and anti-HIV-1 activities (syncytium inhibition assay using the ^ΔTat/Rev^MC99 virus and 1A2 cell line system and reverse transcriptase (RT) assay). The results are shown in Tables 2 and 3, respectively.

**Table 2:** Cytotoxic activities of some isolated compounds

Compounds	Cell lines (IC_50_, µM)						
	P-388	KB	HT-29	MCF-7	A549	ASK	HEK-293
**1**	2.51	2.22	7.72	2.92	5.15	5.26	4.50
**2**	5.36	5.43	–	4.82	6.92	–	6.14
**3**	–	–	–	–	–	–	–
**7**	9.73	8.96	–	9.04	14.15	–	10.19
**8**	–	–	–	–	–	–	–
**9**	1.63	1.63	–	7.31	4.06	2.10	9.89
**12**	–	–	–	–	–	–	–
**13**	–	–	–	–	–	–	–
**14**	–	–	–	–	–	–	–
**16**	–	–	–	–	–	–	–
**17**	–	–	–	–	–	–	–
**Elipticine**	1.74	2.27	2.23	1.67	1.95	2.36	1.71

^a^Results are expressed as IC_50_ (μM): IC_50_ < 20 μM is considered active. P-388 = murine lymphocytic leukemia, KB = human nasopharyngeal carcinoma, HT-29 = human colon cancer, MCF-7 = human breast cancer, A549 = human lung cancer, ASK = cell line from rat glioma, HEK-293 = normal human embryonic kidney cells

**Table 3:** Anti-HIV activities of some isolated compounds

Compounds	Anti-HIV activities						
	Cytotoxic and syncytium inhibition^a^ assays				HIV-1 RT^b^		
	IC_50_ (μM)	EC_50_ (μM)	SI	Activity	%Inhibition at 200 μg/mL	IC_50_ (μM)	Activity
**1**	–	–	–	T	64.04	–	M
**2**	–	–	–	T	88.18	23.61	VA
**3**	–	–	–	T	77.30	42.72	VA
**7**	170.71	28.17	6.06	A	73.49	102.34	VA
**8**	> 507.59	271.66	> 1.87	A	39.54	–	I
**9**	–	–	–	T	98.24	214.31	VA
**12**	> 226.44	> 226.44	–	I	− 108.29	–	I
**13**	> 293.42	> 293.42	–	I	15.94	–	I
**14**	> 282.81	> 282.81	–	I	6.96	–	I
**16**	> 282.81	> 282.81	–	I	−3.33	–	I
**17**	> 282.81	> 282.81	–	I	40.90	–	I

^a^Syncytium inhibition assay: EC_50_ = dose of compound that reduced 50% syncytium formation by ^ΔTat/Rev^MC99 virus in 1A2 cells. IC_50_ = dose of compound that inhibited 50% metabolic activity of uninfected cells 1A2 cells. AZT was used as a positive control for syncytium inhibition assay and averaged from two experiments, EC_50_ > 3.29 × 10^–3^ µM, IC_50_ > 10^–2^ µM and SI > 3.45. SI, selectivity index: IC_50_/EC_50_. Activity: A, active (SI > 1); T, toxic (IC_50_ is less than the lowest concentration tested); I, inactive

^b^RT assay: Compounds were prescreened at 200 μg/ml. Activity; VA = active (> 70% inhibition); M = moderate (> 50% to 70% inhibition); W = weak (> 30–50% inhibition); I = inactive (< 30% inhibition). Fagaronine chloride and nevirapine were used as a positive control for anti-HIV-1 RT assay; IC_50_ fagaronine chloride, 26.40 μM; IC_50_ nevirapine, 7.62 μM averaged from three independent experiments

### Cytotoxic activity

For cytotoxicity, compounds were screened using ellipticine as a positive control (Table 2). Among eleven compounds screened, compounds **1**, **2**, **7**, and **9** exhibited significant cytotoxic activity. Compound **1** showed promising cytotoxic activity against all tested cell lines with IC_50_ in a range of 2.22 − 7.72 µM. Compounds **2** (IC_50_ 4.82 − 6.92 µM) and **7** (IC_50_ 8.96 − 14.15 µM) were found active against P-388, KB, MCF-7, and A-549 cell lines. Compound **9** displayed cytotoxic activity in almost all tested cell lines except HT-29 with IC_50_ values ranging from 1.63 − 9.89 µM. However, compounds **1**, **2**, **7**, and **9** were also toxic against noncancerous HEK-293 cells.

### Anti-HIV activity

The results of anti-HIV activity are shown in Table 3. In the reverse transcriptase assay, compounds **2**,** 3**,** 7**, and** 9** demonstrated inhibitory activity against HIV-1 RT. Compound** 2** exhibited the most potent inhibition with an IC_50_ value of 23.61 µM. While this value is higher than that of the clinically established NNRTI nevirapine (IC_50_ 7.62 µM), it represents a promising lead for a natural scaffold, performing comparably to the alkaloid control fagaronine chloride (IC_50_ 26.40 µM). Due to its significant inhibitory profile and its potential to target the allosteric NNRTI binding site, compound **2** was selected for further molecular docking analysis to elucidate its structural mechanism of action and provide a rationale for its observed in vitro activity.

**Fig. 5:**
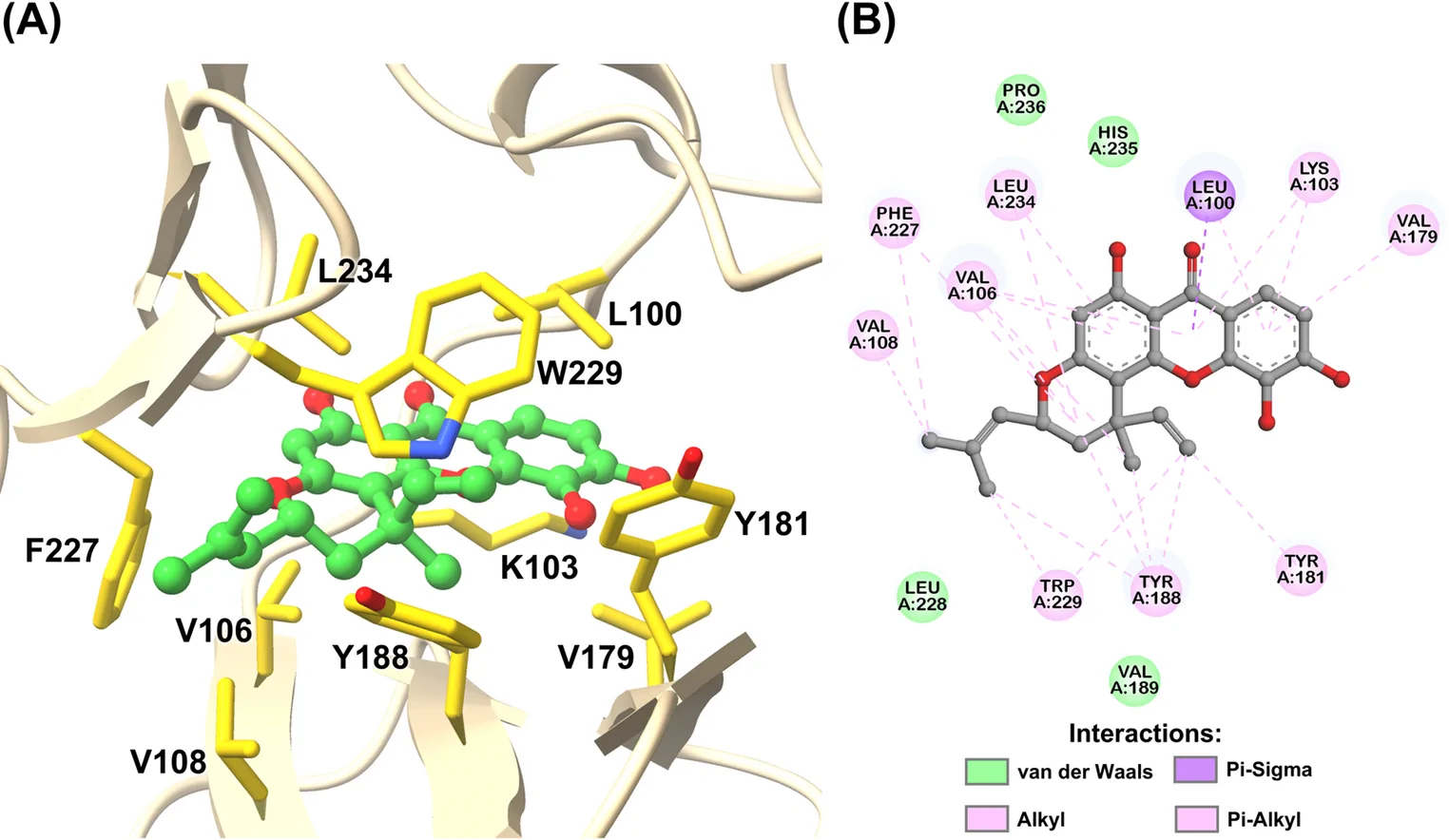
Binding prediction of compound **2** with HIV-1 RT. The molecular docking results of compound **2** bound to HIV-1 RT are represented as **A** a 3D close-up view of the HIV-1 RT binding site and **B** a 2D interaction diagram of the compound **2**–HIV-1 RT complex

To provide a structural rationale for the observed in vitro anti-HIV-1 RT activity of compound **2**, a molecular docking study was performed. To rationalize the binding of compound **2** to HIV-1 RT, the predicted binding profile of compound **2** was evaluated against the well-characterized allosteric binding pocket of NNRTIs. The model indicated that compound **2** binds to this site and interacts with several key residues (Fig. 5A). Notably, residues L100, K103, V106, V108, V179, Y181, Y188, F227, W229, and L234 play crucial roles in stabilizing the interaction, primarily through π-sigma, π-alkyl, and alkyl interactions (Fig. 5B). According to the literature, these specific residues are characterized as essential for the binding of FDA-approved NNRTIs such as nevirapine [35], etravirine [36], and rilpivirine [37]; therefore, their involvement suggests that compound **2** may serve as a promising NNRTI. Overall, these findings offer a plausible mechanism of action for compound **2** that is consistent with our experimental data, highlighting its potential as a promising candidate for further structural optimization to improve its potency and safety profiles.

## Conclusion

In summary, the present work described the previously undescribed polyprenylated xanthones, garcinuntones A‒C (**1**‒**3**) along with fifteen known compounds from the extracts of the leaves and twigs of *G. nuntasaenii*. The structures of the new compounds were assigned on the basis of their spectroscopic data as well as X-ray diffraction analysis. Garcinuntones A (**1**) displayed significant cytotoxic activity against a panel of cultured mammalian cancer cell lines (EC_50_ ranging from 2.22‒7.72 µM) while garcinuntones B (**2**) exhibited interesting anti-HIV-1 activity against reverse transcriptase (RT) with IC_50_ value of 23.61 µM.

## Experimental section

### General experimental procedures

The melting points (uncorrected) were measured on a digital Sanyo Gallenkamp and recorded in Celsius (°C). The major bands (ν_max_) of IR spectra recorded in wave number (cm^−1^) were obtained on a Perkin Elmer EX FT-IR system spectrometer or a Bruker FT-IR spectrometer, model ALPHA. The absorption bands (λ_max_) recorded as wavelengths (nm) and log *ε* in methanol or acetonitrile of UV absorption spectra were performed on a JASCO V-530 spectrometer. ECD spectra were recorded on a JASCO J-810 spectrometer. Optical rotations were measured on a JASCO DIP-370 digital polarimeter by using 10 mm microcell. High resolution mass spectra (HRMS) were recorded on a Bruker Micromass model VQ-TOF spectrometer. ^1^H NMR and ^13^C NMR spectra were recorded on a Bruker DPX-400 or a Bruker Avance-500 spectrometer in part per million (*δ*, ppm) using tetramethylsilane (TMS) or residual non-deuterated solvent peak as an internal reference. Chromatographic methods were performed by using silica gel 60 (Merck, 70–230 mesh) for column chromatography and silica gel plates (Merck, silica gel 60 PF254) for preparative TLC and Sephadex LH-20 (Merck) for gel filtration. Anisaldehyde reagent was used as spraying solution for monitoring spots on TLC after visualization under ultraviolet light either at λ_max_ 254 and/or λ_max_ 366 nm. All solvents used for extraction and isolation were distilled at their boiling point ranges prior to use.

### Plant material

The leaves and twigs of *Garcinia nuntasaenii* Ngern. & Suddee (Clusiaceae) were collected from Bung Khla District, Bueng Kan Province, Thailand (lat. 18° 16′ 630″ N, long. 103° 52′ 983″ E; altitude 125 m a.s.l.) in December 2004. The plant was identified by Narong Nuntasaen. A voucher specimen (BKF 188166) has been deposited at the Forest Herbarium, Department of National Parks, Wildlife and Plant Conservation, Bangkok, Thailand.

### Extraction and isolation

The air-dried leaves and twigs (4.8 kg) were crushed and sequentially percolated with hexanes (3 × 16 l, each three days), EtOAc (3 × 15 l, each three days) followed by MeOH (2 × 15 l, each three days) at room temperature﻿. Each solvent of the extracts was evaporated by a rotary evaporator followed by freeze-drying to provide crude hexanes extract (109.9 g), crude EtOAc extract (129.1 g), and crude MeOH extract (204.9 g).

### Separation of the crude hexanes extract

A portion of crude hexanes extract (100.0 g) was first fractionated by vacuum liquid chromatography (VLC) over silica gel on a sintered glass funnel eluted with hexanes-EtOAc gradient system (1:0 to 0:1 v/v), followed by MeOH to give eight fractions (A1‒A8). Fraction A2 (24.9 g) was further separated by silica gel column chromatography (CC) eluted by hexanes-EtOAc gradient system (1:0 to 0:1 v/v) to provide seven subfractions (B1‒B7). Compounds **13** (213.4 mg) and **18** (733.5 mg) were obtained from subfraction B3 (18.0 g) by crystallization from MeOH. Fraction A3 (26.5 g) was subjected to silica gel CC eluted with hexanes-EtOAc gradient (1:0 to 0:1 v/v) to afford eight subfractions (C1‒C8). Purification of subfractions C1 (332.3 mg) and C2 (3.65 g) by recrystallization with EtOAc-hexanes and C4 (972.6 mg) by silica gel CC eluting with 4% acetone-hexanes provided compounds **13** (50.4 mg), **14** (15.1 mg), and **18** (3.65 g), respectively. Fraction A4 (7.57 g) was separated on silica gel CC eluted with hexanes-EtOAc gradient (1:0 to 0:1 v/v) to yield **15** (10.1 mg) and **17** (161.1 mg).

### Separation of the crude EtOAc extract

A portion of crude EtOAc extract (125 g) was first subjected to a coarse separation by VLC over silica gel on a sintered glass funnel eluted with CH_2_Cl_2_-acetone gradient system (1:0 to 0:1 v/v), followed by MeOH to provide five fractions (A1‒A5). Fraction A1 (186.3 mg) gave compound **16** (77.2 mg) after silica gel CC (15% EtOAc-hexanes). Fraction A2 (11.3 g) was purified by silica gel CC eluted with hexanes-EtOAc gradient system (1:0 to 0:1 v/v) to give seven subfractions (B1‒B7). Subfraction B5 (2.47 g) was further repeatedly fractionated over Sephadex LH-20 CC eluted with CH_2_Cl_2_-MeOH (1:1 v/v) to provide eight subfractions (C1‒C8). Subfraction C3 (182.6 mg) was further purified by Sephadex LH-20 CC eluted with CH_2_Cl_2_-MeOH (1:1 v/v) to yield compounds **1** (50.7 mg), **3** (6.6 mg) and **6** (7.7 mg). Subfraction C4 (248.6 mg) was further purified by silica gel CC eluted with hexanes-EtOAc gradient system (4:1 to 3:2 v/v) provided **5** (2.5 mg). Subfraction B6 (3.78 g) was further purified by Sephadex LH-20 CC eluted with CH_2_Cl_2_-MeOH (1:1 v/v) followed by silica gel CC eluted with hexanes-CH_2_Cl_2_ gradient system (1:0 to 0:1 v/v) to afford six subfractions (D1‒D6). Subfraction D2 (47.5 mg) was purified by Sephadex LH-20 CC eluted with CH_2_Cl_2_-MeOH (1:1 v/v) give compound **2** (36.0 mg). Fraction A3 (48.4 g) was further purified by silica gel CC eluted with hexanes-EtOAc gradient system (1:0 to 0:1 v/v) followed by Sephadex LH-20 CC eluted with CH_2_Cl_2_-MeOH (1:1 v/v) to yield compound **12** (42.9 mg). After fractionation by Sephadex LH-20 CC eluted with CH_2_Cl_2_-MeOH (1:1 v/v) followed by silica gel CC eluted with 2% MeOH in hexanes-EtOAc (3:2 v/v), fraction A4 (28.2 g) provided seven subfractions (E1‒E7). Compound **11** (400.8 mg) was obtained from subfraction E5.

### Separation of the crude MeOH extract

The crude MeOH extract (204.9 g) was dissolved in MeOH-EtOAc (1:1 v/v, 1 l). After filtration, the insoluble portion (42.2 g) was obtained and was not further investigated. The crude MeOH-EtOAc (1:1 v/v) soluble fraction (162.1 g) was obtained after removal solvent from filtrate under reduce pressure. The fractionation of this fraction was performed by VLC over silica gel CC on sintered glass funnel eluted with hexanes-acetone gradient system (1:0 to 0:1 v/v). The collected fractions were combined on basis of their TLC behaviors to give five fractions (A1‒A5). Fraction A2 (2.73 g) was further fractionated on Sephadex LH-20 CC eluted with CH_2_Cl_2_-MeOH (1:1 v/v) to give eleven subfractions (B1‒B11). Compounds **6** (8.5 mg) and **8** (7.8 mg) were obtained after purification of the subfraction B2 (106.6 mg) on Sephadex LH-20 CC eluted with CH_2_Cl_2_-MeOH (1:1 v/v). Subfractions B5 (209.0 mg) was purified on Sephadex LH-20 CC eluted with CH_2_Cl_2_-MeOH (1:1 v/v) to yield compound **10** (1.3 mg). Subfractions B7 (242.2 mg) was purified by Sephadex LH-20 CC eluted with CH_2_Cl_2_-MeOH (1:1 v/v) to provide compounds **7** (87.6 mg) and **9** (45.5 mg). Fraction A3 (28.5 g) was repeatedly fractionated by silica gel CC eluted with EtOAc in MeOH-CH_2_Cl_2_ (1:25 v/v) to provide six subfractions (C1‒C6). Compound **4** (8.0 mg) was obtained after isolation of subfractions C3 (799.9 mg) by Sephadex LH-20 CC eluted with CH_2_Cl_2_-MeOH (1:1 v/v). Subfractions C4 (42.9 g) provided compound **11** (13.3 g) after silica gel CC eluted by EtOAc in MeOH-CH_2_Cl_2_ (1:25 v/v).

### Garcinuntone A (1)

Pale yellow powder from MeOH; mp 119.2–120.5 °C. $\left[ {\upalpha } \right]_{{\mathrm{D}}}^{{{27}}}$ +53.8 (*c* 1.0, MeOH). UV (MeOH) ${\uplambda}_{{{\mathrm{max}}}}$ nm (log *ε*): 236 (4.4), 283 (4.7), 337 (4.3). CD (0.43, mM) nm (Δ*ε*): 395 (+ 0.21), 372 (–0.10), 340 (+ 0.33), 294 (+ 0.74), 277 (+ 0.9), 244 (– 0.76), 224 (+ 0.75); FTIR (Neat) ${\upnu}_{{{\mathrm{max}}}}$ cm^−1^: 3536, 3479, 3331, 3050, 2967, 2913, 2855, 1651, 1610, 1580, 1558, 1528, 1454, 1428, 1370, 1328, 1283, 1264, 1200, 1178, 1152, 1121, 1078, 1152, 1121, 1078, 1037, 991, 957, 912, 889, 869, 857, 834, 797, 770, 723, 650, 622; ^1^H– and ^13^C–NMR data see Table 1. HRESI-MS *m/z* 485.1933 [M + Na]^+^ (Calcd for C_28_H_30_O_6_Na: 485.1935).

### Garcinuntone B (2)

Yellow needles from pyridine/MeOH. mp 172.6–173.9 °C. $\left[ {\upalpha } \right]_{{\mathrm{D}}}^{{{27}}}$ −80.5 (*c* 1.0, MeOH). UV (MeOH) ${\uplambda}_{{{\mathrm{max}}}}$ nm (log *ε*): 255 (4.7), 285 (4.1), 327 (4.4). FTIR (Neat) ${\upnu}_{{{\mathrm{max}}}}$ cm^−1^: 3656, 3548, 3077, 2935, 2912, 1657, 1630, 1606, 1580, 1545, 1475, 1418, 1325, 1299, 1285, 1215, 1204, 1189, 1166, 1128, 1100, 1085, 1056, 1030, 1015, 995, 971, 953, 906, 828, 818, 782, 739, 688, 665, 641, 619, 540, 506. ^1^H– and ^13^C–NMR data see Table 1. HRESI-MS *m/z* 417.1313 [M + Na]^+^ (Calcd for C_23_H_22_O_6_Na: 417.1309).

### Garcinuntone C (3)

Pale yellow powder from MeOH. mp 139.1–141.3 °C. $\left[ {\upalpha } \right]_{{\mathrm{D}}}^{{{27}}}$ −21.7 (*c* 1.0, MeOH). UV (MeOH) ${\uplambda}_{{{\mathrm{max}}}}$ nm (log *ε*): 253 (3.3), 285 (2.7), 328 (3.0). FTIR (Neat) ${\upnu}_{{{\mathrm{max}}}}$ cm^−1^ 3412, 3215, 2974, 2915, 2859, 1622, 1585, 1525, 1512, 1469, 1405, 1324, 1263, 1227, 1208, 1189, 1165, 1129, 1093, 2070, 1038, 1024, 982, 968, 937, 906, 831, 809, 783, 733, 706, 676, 661, 624, 609, 559. ^1^H– and ^13^C–NMR data see Table 1. HRESI-MS *m/z* 397.1643 [M + H]^+^ (Calcd for C_23_H_25_O_6_: 397.1646).

### X-ray crystal structure analysis

X-ray diffraction data were collected using graphite mono-chromated Mo Karadiation (λ = 0.71073 Å) on a Bruker-Nonius kappa CCD diffractometer or on a Bruker APEX2 diffractometer equipped with a graphite monochromated Cu Kα radiation source (λ = 1.54178 Å). Cell refinement and data reduction were performed with Bruker SAINT software. The structure was solved by direct methods using SIR97 [38] and then all atoms except hydrogen atoms were refined anisotropically by a full-matrix least-squares methods on F^2^ using SHELXL-2013 [39]. The absolute configurations were determined by refinement of the Flack parameter [40]. Crystallographic data of compound **2** have been deposited at the Cambridge Crystallographic Data Center under the reference number CCDC 2486312. Copies of the data can be obtained, free of charge via http://www.ccdc.cam.ac.uk/conts/retrieving.htmlor on application to CCDC, 12 Union Road, Cambridge, CB2 1EZ, UK (fax: + 44 1223336033 or e-mail: deposit@ccdc.cam.ac.uk).

### Crystal data of garcinuntone B (2)

The crystals of **2** were obtained from slow evaporation of pyridine/MeOH solution. C_23_H_22_O_6_, MW = 394.42, 0.35 × 0.30 × 0.30 mm, orthorhombic, space group *P*2_1_2_1_2_1_(No. 19). *a* = *9.846(2*) Å, *b* = *14.748(4)* Å, *c* = *16.771(3)* Å, α = *β* = γ = 90°, *V* = *2435.2(9)* Å^3^, *Z* = 4, *T* = 293(2) K, μ(Cu Kα) = 0.744 mm^−1^, *D*_*x*_ = 1.292 g/mm^3^, reflections collected/unique: 4183/3980, number of observations [I > 2σ(I)]: 3726, *R*1 = *0.0452*, w*R*2 = 0.1079 (all data), Flack parameter = 0.13(4). Crystallographic data of compound **2** has been deposited to the Cambridge Crystallographic Data Center (CCDC 2486312).

### Cytotoxic assay

The cytotoxic activity of crude extracts and compounds were performed by using the standard in vitro sulforhodamine B (SRB) assay in 96-well microtiter plates [41]. Ellipticine, a potent cytotoxic plant alkaloid, was used as a positive control. Seven cell lines were employed, including P-388 (murine lymphocytic leukemia), KB (human oral nasopharyngal carcinoma), HT-29 (human colorectal adenocarcinoma), MCF-7 (human breast cancer), A549 (human lung carcinoma), ASK (rat glioma cell), and HEK-293 (normal human kidney cell). The potency of cytotoxic activity is expressed as 50% inhibitory dose (IC_50_).

### Anti-HIV-1 reverse transcriptase assay

For crude extracts and fractions, tannins were removed by following the procedure described by Tan et al. [42]. Briefly, 100 μl of a 20 mg/ml stock solution in DMSO were treated with approximately 100 mg of polyvinylpyrolidone in a microcentrifuge tube. The mixture was shaken and centrifuged at 300 g for 5–10 min at 10 °C. Then, the supernatant was collected for testing. Pure compounds were dissolved in DMSO to obtain a final concentration of 0.2 mg/ml.

In 96-well plate, a standardized amount of HIV-1 reverse transcriptase (Amersham Pharmacia Biotech Asia Pacific Ltd., Hong Kong) and the sample (0.2 mg/mL) were added to the reaction mixture containing radiolabeled thymidine triphosphate ([^3^H]TTP) and polyadenylic acid (polyA) which served as a reverse transcriptase (RT) enzyme substrate and RNA template, respectively. HIV-1 RT activity was detected by measuring the incorporation of [^3^H]TTP to the polyA template. Positive controls included fagaronine chloride and nevirapine, known RT inhibitors, in place of the tested sample, whereas negative controls were wells that had no tested samples. RT enzyme was standardized with fagaronine chloride. The experiments were done in three independent experiments. The data were averaged, and percent inhibition compared with the negative control was calculated. For the samples with greater than 70% inhibition, their 50% inhibitory concentrations (IC_50_) were further determined by testing RT activities in the presence of various concentrations of the samples. The IC_50_ values were then calculated using non-linear regression analysis of the dose–response curves in GraphPad Prism software.

### Syncytium inhibition assay

The syncytium inhibition assay was carried out using the ^ΔTat/Rev^MC99virus and 1A2 cell line system obtained from Dr. D. J. Clanton [43]. Briefly, 1A2 cells prepared in 96-well plates were infected with the ^ΔTat/Rev^MC99 virus in the presence of various concentrations of compounds, ranging from 3.9 to 250 μg/mL. The syncytia formed were counted after incubation at 37 °C for three days. Each concentration was evaluated in triplicate within a single experimental run. The results are expressed as 50% effective concentration (EC_50_), or concentration that inhibited syncytia formation by 50% using non-linear regression. Controls included cells-only, no-compound, and no-virus groups. A known HIV-1 replication inhibitor, zidovudine (or azidothymidine, AZT), was used as a positive control. By using a colorimetric XTT assay, cytotoxicity of the compound was also carried out in parallel in duplicate. The result of cytotoxicity is expressed as the concentration that inhibited formazan formation in uninfected cells by 50% (IC_50_). Data are reported as the mean of replicates. The selectivity index (SI) was calculated using equation SI = IC_50_/EC_50_.

### Molecular docking study

The three-dimensional (3D) structure of wild-type HIV-1 RT was obtained from the Protein Data Bank (PDB ID: 3MEC). The 3D structure of garcinuntone B (**2**) was extracted from our X-ray crystallographic data and subjected to energy minimization using the Molecular Mechanics 2 (MM2) force field in Chem3D software. The protein structure and minimized ligand were then converted into the PDBQT file format using the AutoDockFR 1.0 software suite [44]. Molecular docking was performed with AutoDock Vina 1.2 [45]. The docking center was defined based on the centroid of the co-crystallized ligand in the 3MEC structure. The grid box dimensions were set to 20 × 20 × 20 Å, with center coordinates at x = 9.57, y = 12.62, and z = 16.98. The best docked pose, determined by the lowest binding energy, was selected for further analysis. The 2D interaction diagram was generated using Discovery Studio Visualizer (BIOVIA, San Diego, CA, USA), while the 3D visualization of the protein–ligand binding mode was performed using UCSF ChimeraX [46].

## Supplementary Information

Below is the link to the electronic supplementary material.

Supplementary file1 (PDF 8173 KB)
