# Supplementary material for: Garcinuntones A‒C, three new polyprenylated xanthones with cytotoxic and anti-HIV-1 activities from Garcinia nuntasaenii
Source: J Nat Med. 2026 Jul 1;80(5):1433–45. doi: 10.1007/s11418-026-02060-3 (PMC13547166; doi:10.1007/s11418-026-02060-3)
Supplement: Supplementary file 1 [file 11418_2026_2060_MOESM1_ESM.pdf]

Garcinuntones A–C, three new polyprenylated xanthones with cytotoxic and anti-HIV-1 activities from *Garcinia nuntasaenii*

Suppisak Chaturonrutsamee[1,2,3], Chutima Kuhakarn[1,2], Sariyarach Thanasansurapoung[2], Samran Prabpai[2], Arthit Chairongdua[4], Radeekorn Akkarawongsapat[5], Narong Nuntasaen[2], Sakchai Hongthong[6], Bodee Nutho[7], Vichai Reutrakul[1,2,*]

[1]*Department of Chemistry, Faculty of Science, Mahidol University, Rama VI Road, Bangkok 10400, Thailand*

[2]*Center of Excellence for Innovation in Chemistry (PERCH-CIC), Faculty of Science, Mahidol University, Rama VI Road, Bangkok 10400, Thailand*

[3]*Research and Innovation Department, International Laboratories Corp., Ltd., Bang Phli, Samut Prakan 10540, Thailand*

[4]*Department of Physiology, Faculty of Science, Mahidol University, Rama VI Road, Bangkok 10400, Thailand*

[5]*Department of Microbiology, Faculty of Science, Mahidol University, Rama VI Road, Bangkok 10400, Thailand*

[6]*Science and Innovation for Development Program, and Division of Chemistry, Faculty of Science and Technology, Rajabhat Rajanagarindra University, Chachoengsao 24000, Thailand*

[7]*Department of Pharmacology, Faculty of Science, Mahidol University, Bangkok 10400, Thailand*

Correspondence

Prof. Vichai Reutrakul

Department of Chemistry, and Center of Excellence for Innovation in Chemistry (PERCH-CIC), Faculty of Science Mahidol University Bangkok 10400, Thailand

Phone: + 66 (2) 201 5152

vichai.reu@mahidol.ac.th; ORCID: 0000-0002-0743-1300

Content

Figure S1. 500 MHz ^{1}H NMR spectrum of compound **1** in acetone-d_6 5
Figure S2. 125 MHz ^{13}C NMR spectrum of compound **1** in acetone-d_6 5
Figure S3. ^{1}H-^{1}H COSY spectrum of compound **1** in acetone-d_6 6
Figure S4. HMQC spectrum of compound **1** in acetone-d_6 6
Figure S5. HMBC spectrum of compound **1** in acetone-d_6 7
Figure S6. NOESY spectrum of compound **1** in acetone-d_6 7
Figure S7. HRMS spectrum of compound **1** 8
Figure S8. 500 MHz ^{1}H NMR spectrum of compound **2** in pyridine-d_5 9
Figure S9. 125 MHz ^{13}C NMR spectrum of compound **2** in pyridine-d_5 9
Figure S10. ^{1}H-^{1}H COSY spectrum of compound **2** in pyridine-d_5 10
Figure S11. HMQC spectrum of compound **2** in pyridine-d_5 10
Figure S12. HMBC spectrum of compound **2** in pyridine-d_5 11
Figure S13. NOESY spectrum of compound **2** in pyridine-d_5 11
Figure S14. 500 MHz ^{1}H NMR spectrum of compound **2** in acetone-d_6 12
Figure S15. 125 MHz ^{13}C NMR spectrum of compound **2** in acetone-d_6 12
Figure S16. ^{1}H-^{1}H COSY spectrum of compound **2** in acetone-d_6 13
Figure S17. HMQC spectrum of compound **2** in acetone-d_6 13
Figure S18. HMBC spectrum of compound **2** in acetone-d_6 14
Figure S19. NOESY spectrum of compound **2** in acetone-d_6 14
Figure S20. HRMS spectrum of compound **2** 15
Figure S21. 500 MHz ^{1}H NMR spectrum of compound **3** in pyridine-d_5 16
Figure S22. 125 MHz ^{13}C NMR spectrum of compound **3** in pyridine-d_5 16
Figure S23. ^{1}H-^{1}H COSY spectrum of compound **3** in pyridine-d_5 17
Figure S24. HMQC spectrum of compound **3** in pyridine-d_5 17
Figure S25. HMBC spectrum of compound **3** in pyridine-d_5 18
Figure S26. NOESY spectrum of compound **3** in pyridine-d_5 18
Figure S27. HRMS spectrum of compound **3** 19
Figure S28. 500 MHz ^{1}H NMR spectrum of compound **4** in DMSO-d_6 20
Figure S29. 125 MHz ^{13}C NMR spectrum of compound **4** in DMSO-d_6 20
Figure S30. HRMS spectrum of compound **4** 21
Figure S31. 400 MHz ^{1}H NMR spectrum of compound **5** in acetone-d_6 22
Figure S32. 100 MHz ^{13}C NMR spectrum of compound **5** in acetone-d_6 22
Figure S33. HRMS spectrum of compound **5** 23
Figure S34. 500 MHz ^{1}H NMR spectrum of compound **6** in DMSO-d_6 24
Figure S35. 125 MHz ^{13}C NMR spectrum of compound **6** in DMSO-d_6 24
Figure S36. HRMS spectrum of compound **6** 25

Figure S37. 500 MHz ^{1}H NMR spectrum of compound **7** in acetone-d_6 26
Figure S38. 125 MHz ^{13}C NMR spectrum of compound **7** in acetone-d_6 26
Figure S39. HRMS spectrum of compound **7** 27
Figure S40. 500 MHz ^{1}H NMR spectrum of compound **8** in acetone-d_6 28
Figure S41. 125 MHz ^{13}C NMR spectrum of compound **8** in acetone-d_6 28
Figure S42. HRMS spectrum of compound **8** 29
Figure S43. 500 MHz ^{1}H NMR spectrum of compound **9** in CD_3OD 30
Figure S44. 125 MHz ^{13}C NMR spectrum of compound **9** in CD_3OD 30
Figure S45. HRMS spectrum of compound **9** 31
Figure S46. 500 MHz ^{1}H NMR spectrum of compound **10** in acetone-d_6 32
Figure S47. 125 MHz ^{13}C NMR spectrum of compound **10** in acetone-d_6 32
Figure S48. HRMS spectrum of compound **10** 33
Figure S49. 500 MHz ^{1}H NMR spectrum of compound **11** in DMSO-d_6 34
Figure S50. 125 MHz ^{13}C NMR spectrum of compound **11** in DMSO-d_6 34
Figure S51. HRMS spectrum of compound **11** 35
Figure S52. 500 MHz ^{1}H NMR spectrum of compound **12** in DMSO-d_6 36
Figure S53. 125 MHz ^{13}C NMR spectrum of compound **12** in DMSO-d_6 36
Figure S54. HRMS spectrum of compound **12** 37
Figure S55. 500 MHz ^{1}H NMR spectrum of compound **13** in $CDCl_3$ 38
Figure S56. 125 MHz ^{13}C NMR spectrum of compound **13** in $CDCl_3$ 38
Figure S57. HRMS spectrum of compound **13** 39
Figure S58. 500 MHz ^{1}H NMR spectrum of compound **14** in $CDCl_3$ 40
Figure S59. 125 MHz ^{13}C NMR spectrum of compound **14** in $CDCl_3$ 40
Figure S60. HRMS spectrum of compound **14** 41
Figure S61. 500 MHz ^{1}H NMR spectrum of compound **15** in $CDCl_3$ 42
Figure S62. 125 MHz ^{13}C NMR spectrum of compound **15** in $CDCl_3$ 42
Figure S63. HRMS spectrum of compound **15** 43
Figure S64. 500 MHz ^{1}H NMR spectrum of compound **16** in $CDCl_3$ 44
Figure S65. 125 MHz ^{13}C NMR spectrum of compound **16** in $CDCl_3$ 44
Figure S66. HRMS spectrum of compound **16** 45
Figure S67. 500 MHz ^{1}H NMR spectrum of compound **17** in $CDCl_3$ 46
Figure S68. 125 MHz ^{13}C NMR spectrum of compound **17** in $CDCl_3$ 46
Figure S69. HRMS spectrum of compound **17** 47
Figure S70. 500 MHz ^{1}H NMR spectrum of compound **18** in $CDCl_3$ 48
Figure S71. 125 MHz ^{13}C NMR spectrum of compound **18** in $CDCl_3$ 48
Figure S72. HRMS spectrum of compound **18** 49
Table S1. Preliminary cytotoxicity screening of crude extracts 50

Table S2. Preliminary anti-HIV-1 activity screening of crude extracts 50

Figure S73. Dose-dependent anti-HIV-1 activities of isolated compounds. 50

Figure S1. 500 MHz ^{1}H NMR spectrum of compound **1** in acetone-d_6

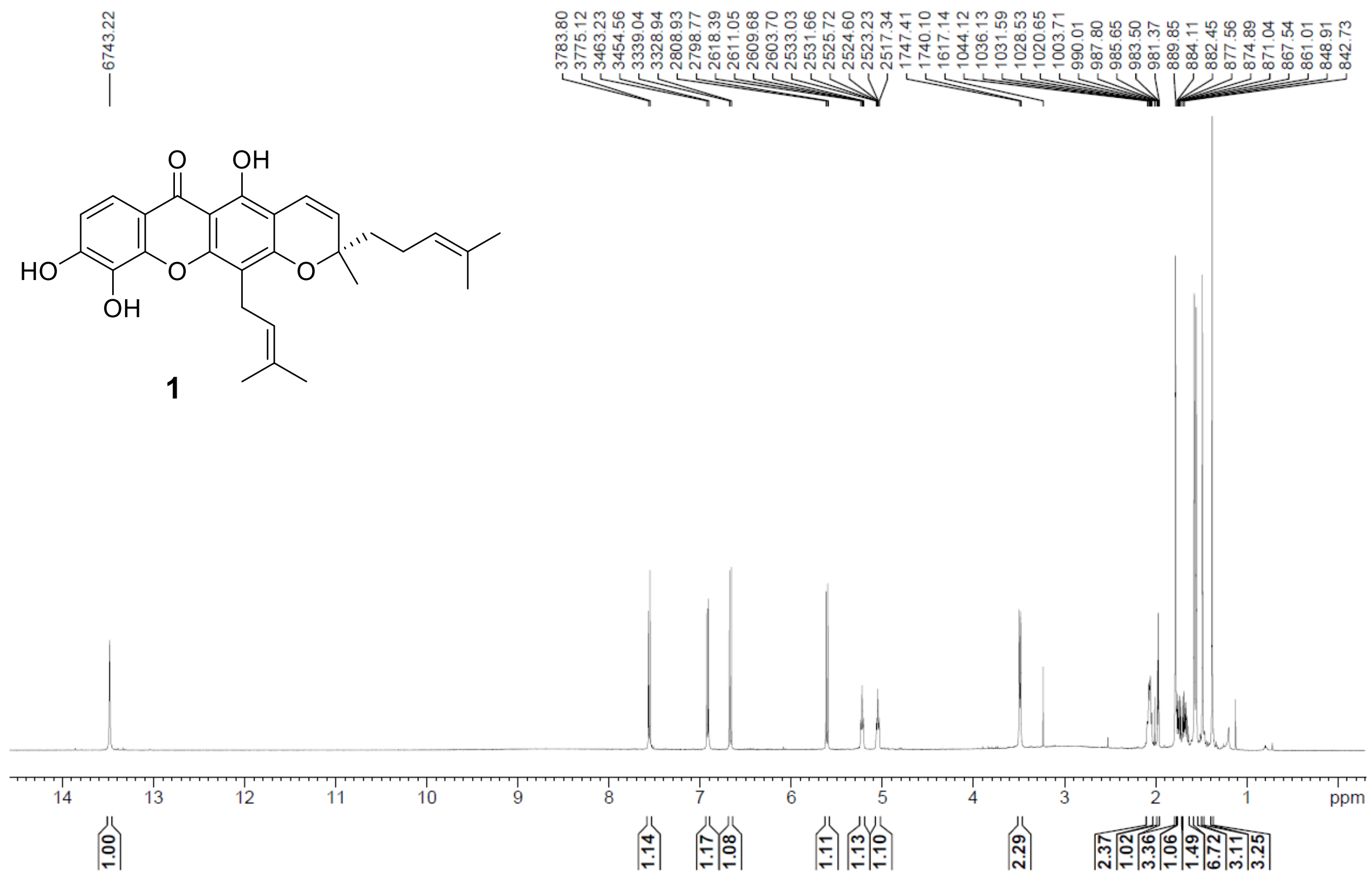

Figure S2. 125 MHz ^{13}C NMR spectrum of compound **1** in acetone-d_6

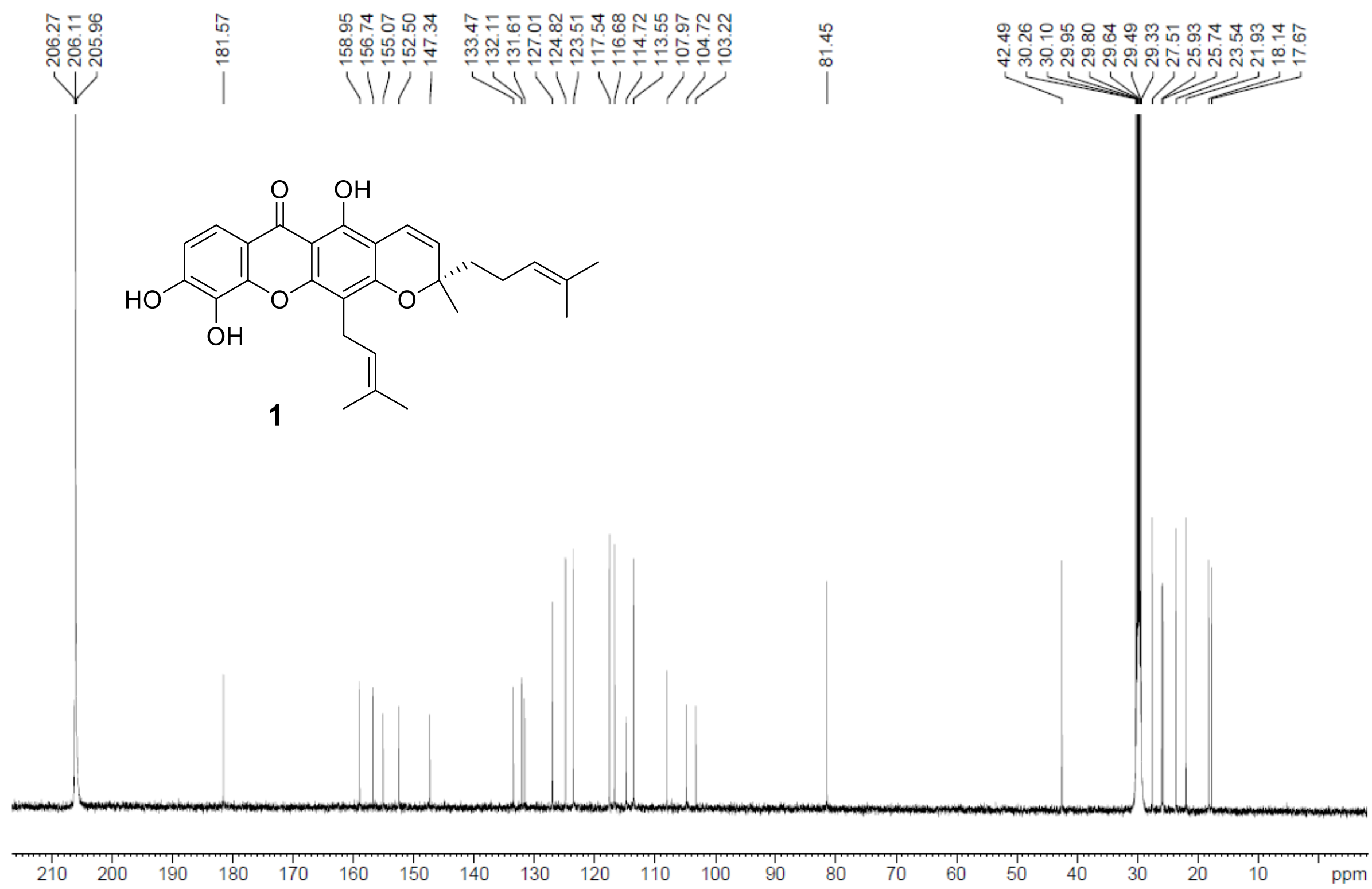

Figure S3. ^{1}H-^{1}H COSY spectrum of compound **1** in acetone-d_6

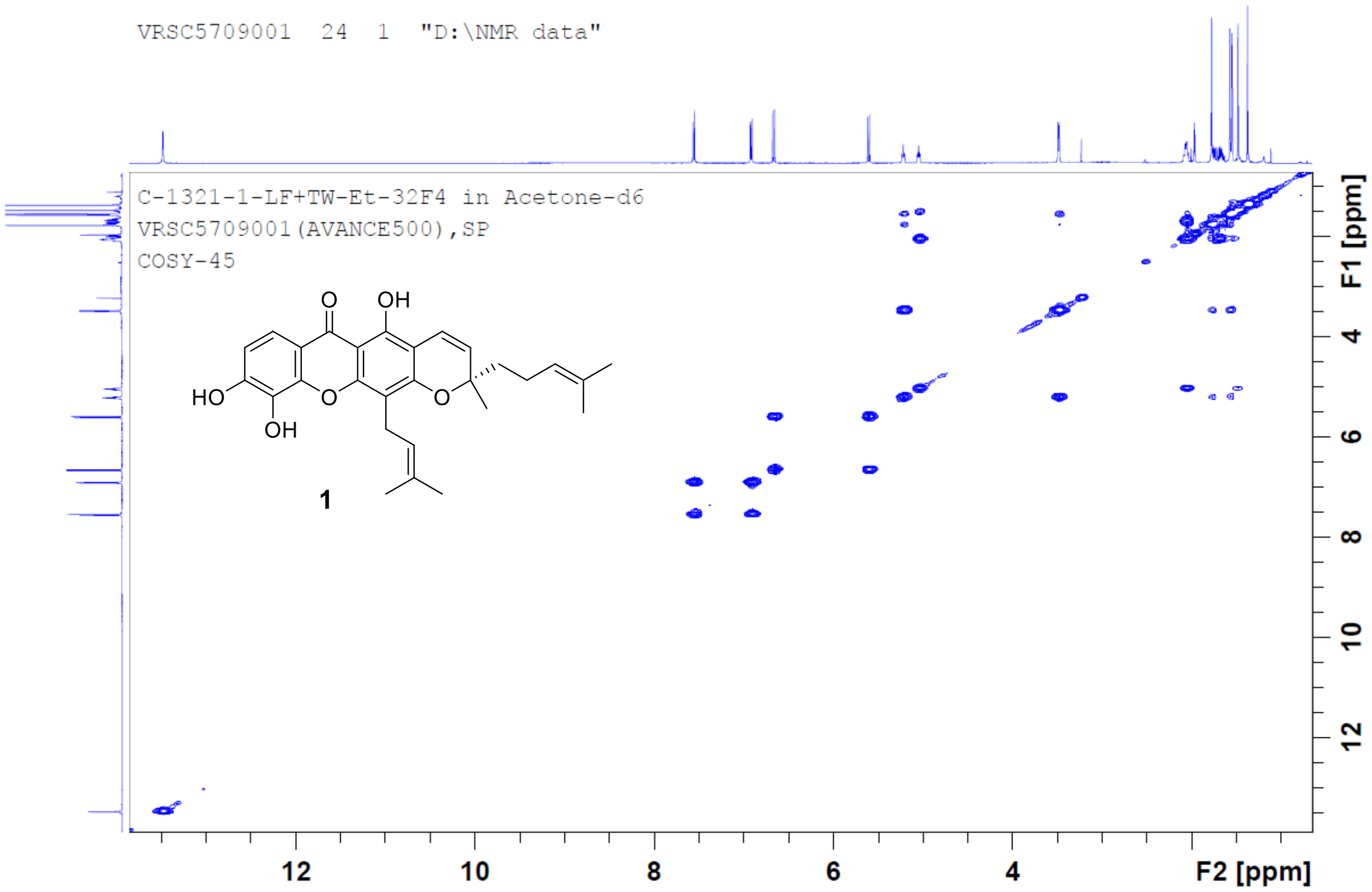

Figure S4. HMQC spectrum of compound **1** in acetone-d_6

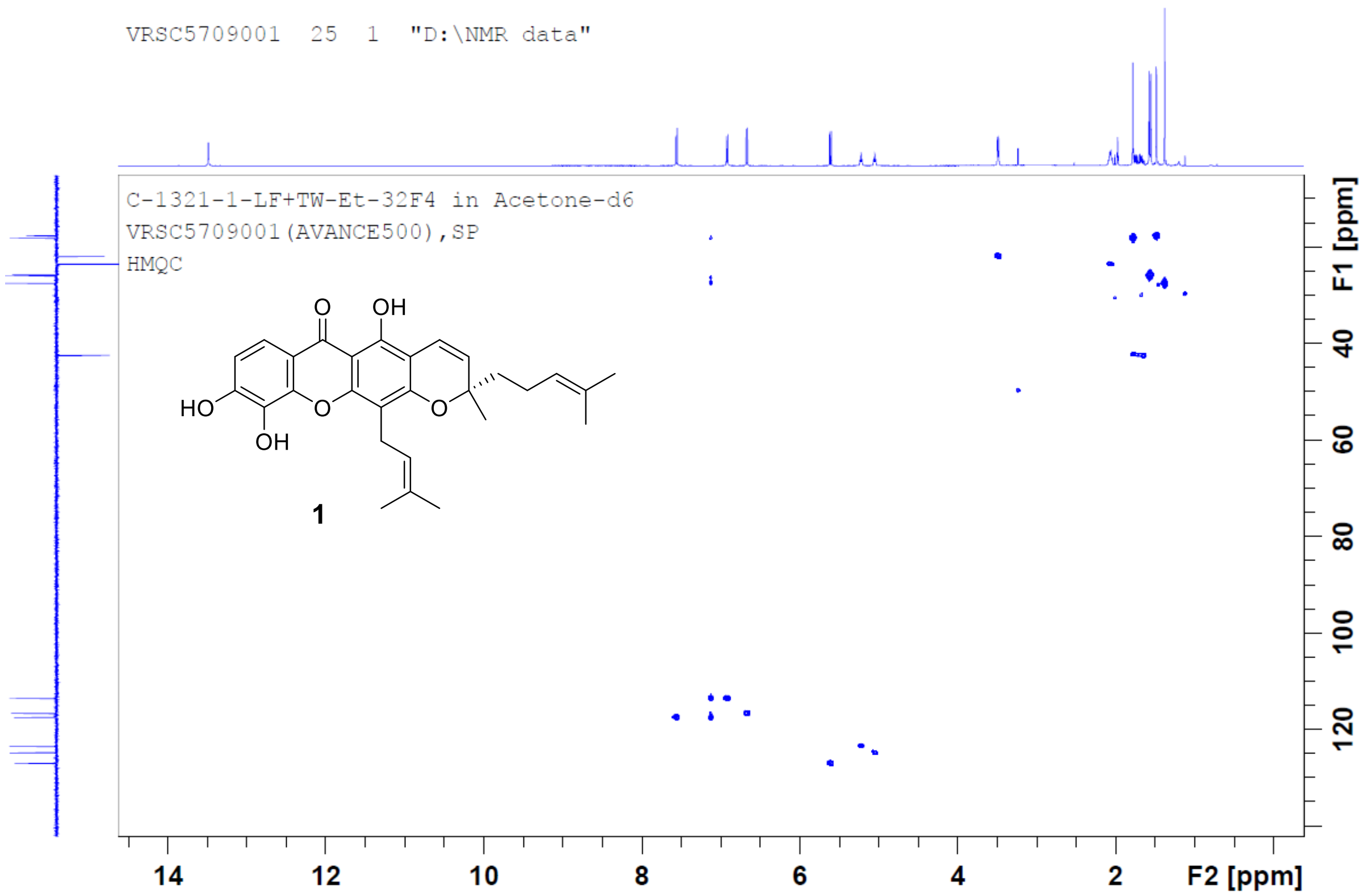

Figure S5. HMBC spectrum of compound **1** in acetone-d_6

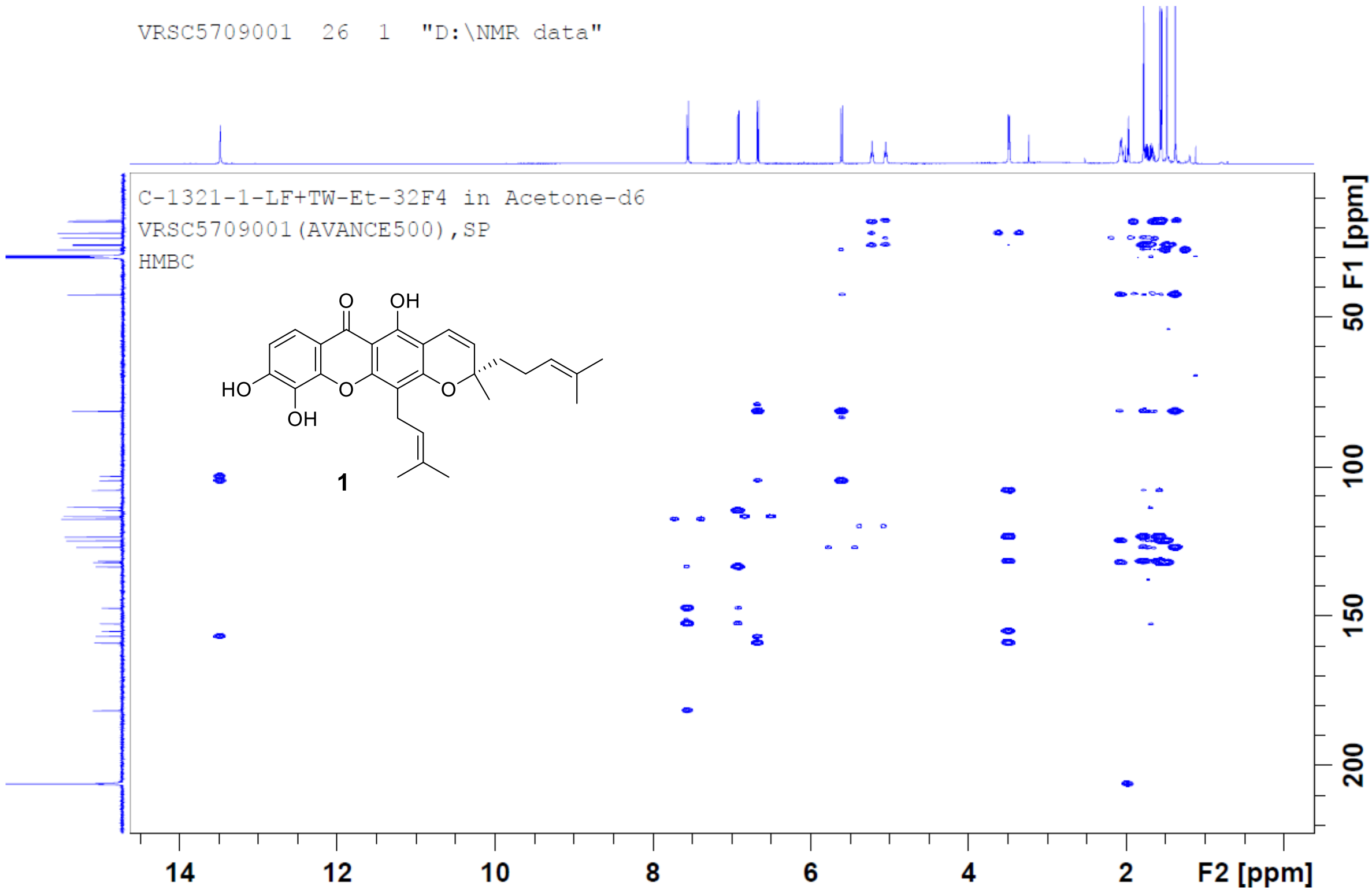

Figure S6. NOESY spectrum of compound **1** in acetone-d_6

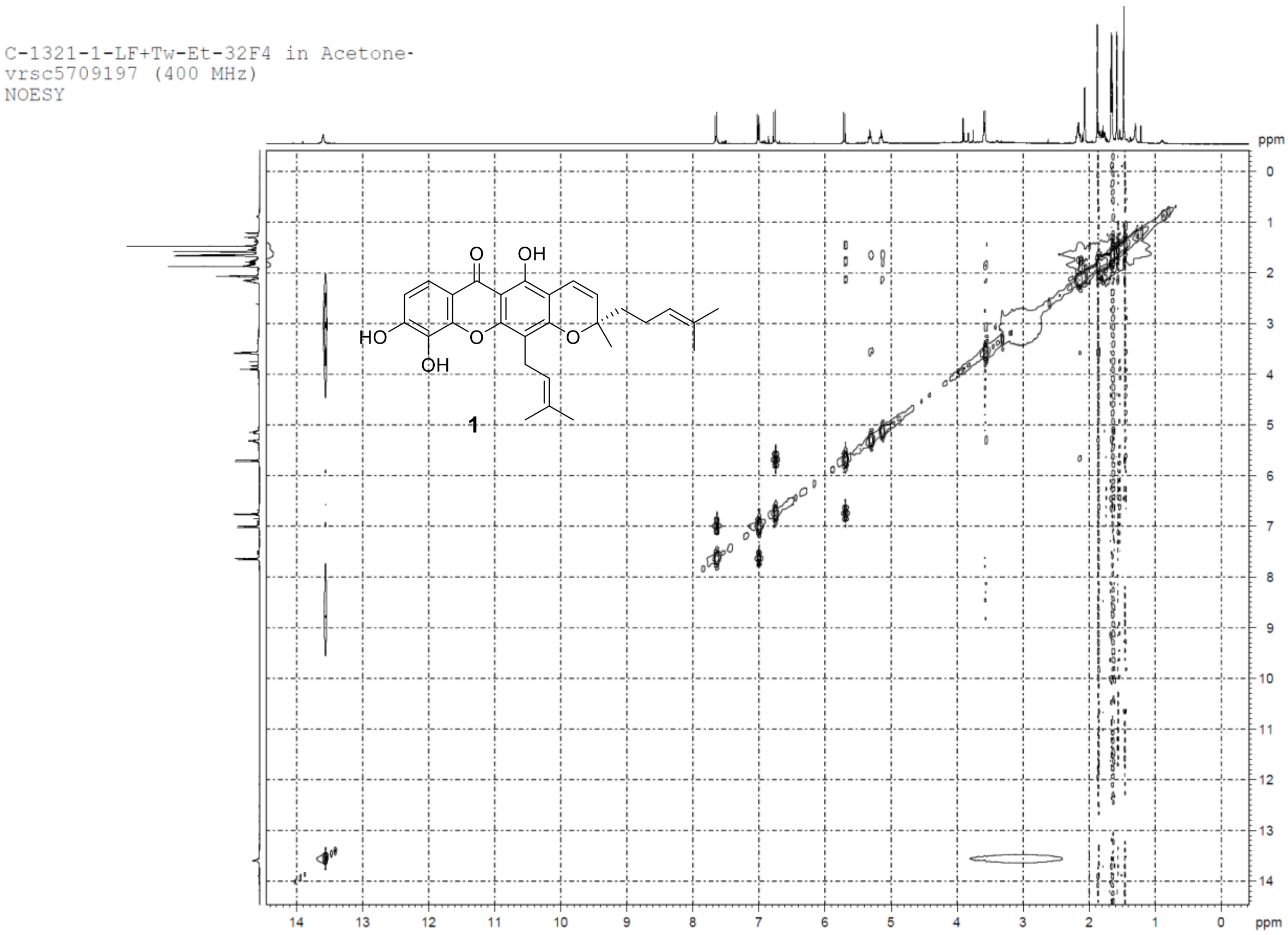

Figure S7. HRMS spectrum of compound **1**

Mass Spectrum List Report

Analysis Info

Analysis Name	VRSC571027001.d	Acquisition Date	10/27/2014 12:23:42 PM
Method	MKE_tune_wide_20130204.m	Operator	Administrator
Sample Name	C-1321-1-LF+TW-Et-32F4	Instrument	micrOTOF 72
	C-1321-1-LF+TW-Et-32F4		

Acquisition Parameter

				Set Corrector Fill	79 V
Source Type	ESI	Ion Polarity	Positive	Set Pulsar Pull	406 V
Scan Range	n/a	Capillary Exit	230.0 V	Set Pulsar Push	388 V
Scan Begin	50 m/z	Hexapole RF	400.0 V	Set Reflector	1300 V
Scan End	3000 m/z	Skimmer 1	45.0 V	Set Flight Tube	9000 V
		Hexapole 1	25.0 V	Set Detector TOF	1910 V

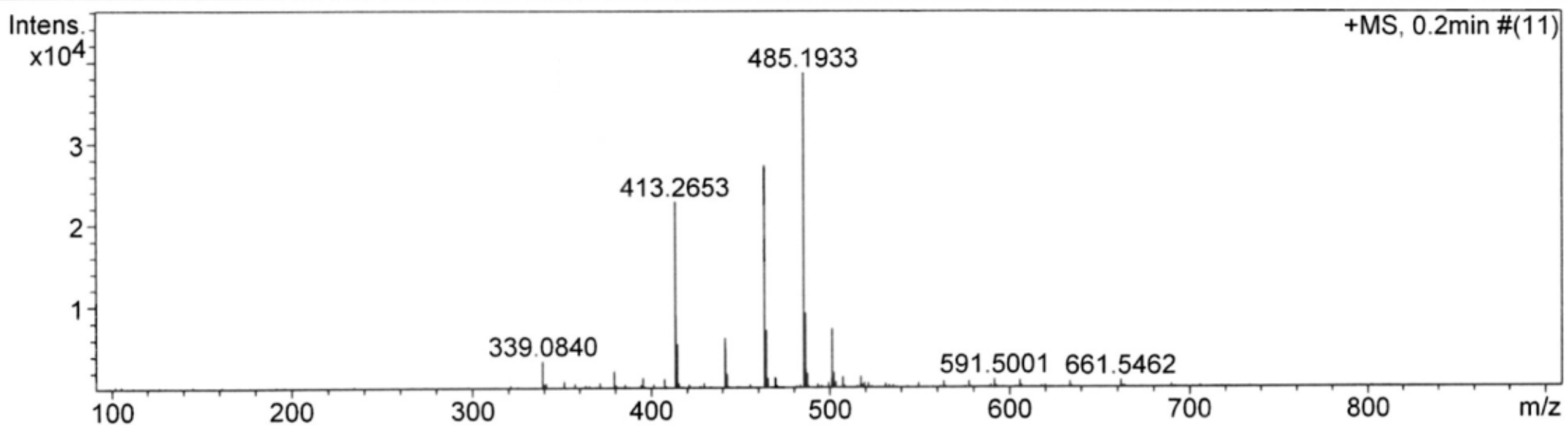

Figure S8. 500 MHz ^{1}H NMR spectrum of compound **2** in pyridine-d_5

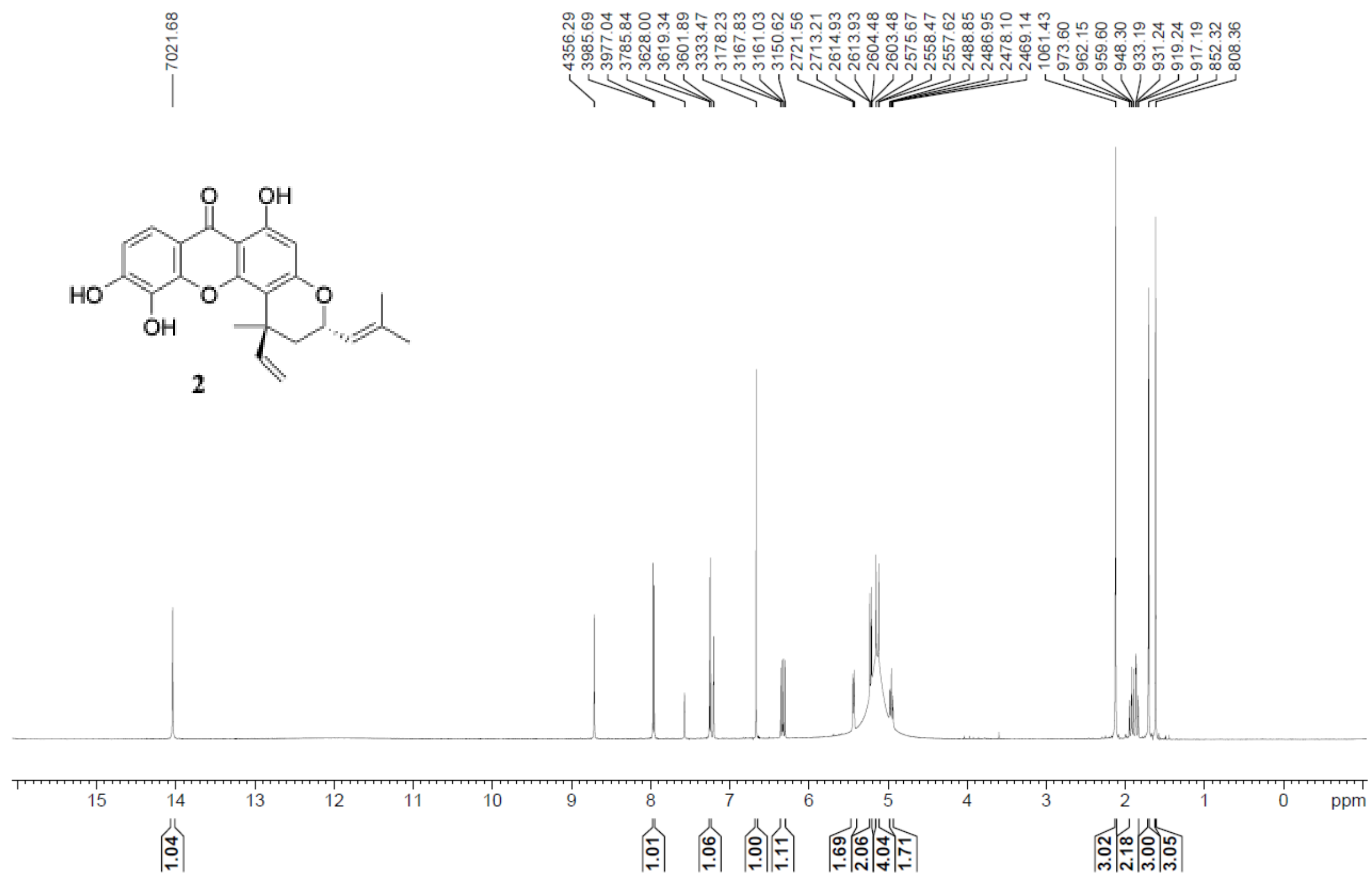

Figure S9. 125 MHz ^{13}C NMR spectrum of compound **2** in pyridine-d_5

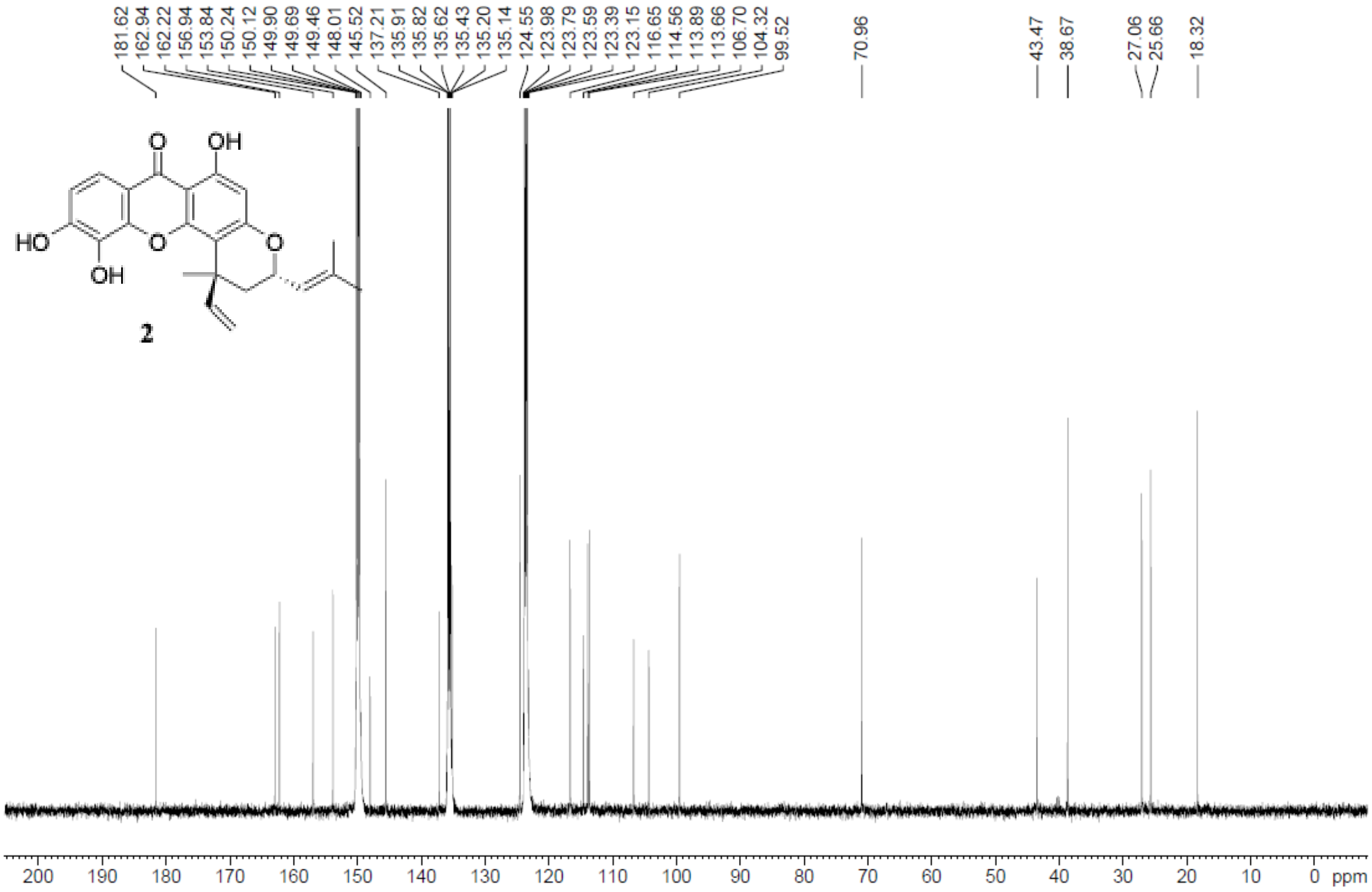

Figure S10. ^{1}H-^{1}H COSY spectrum of compound **2** in pyridine-d_5

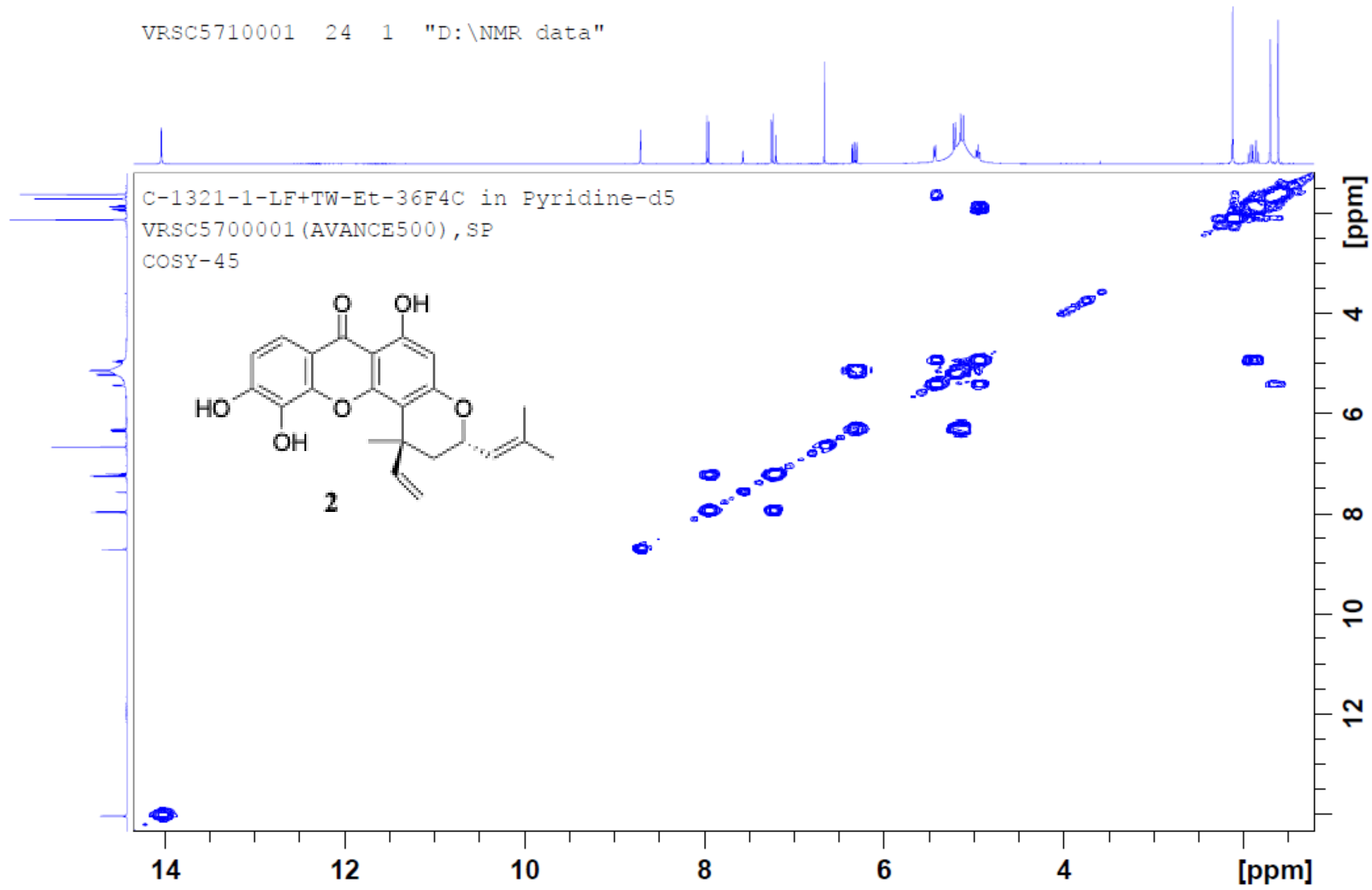

Figure S11. HMQC spectrum of compound **2** in pyridine-d_5

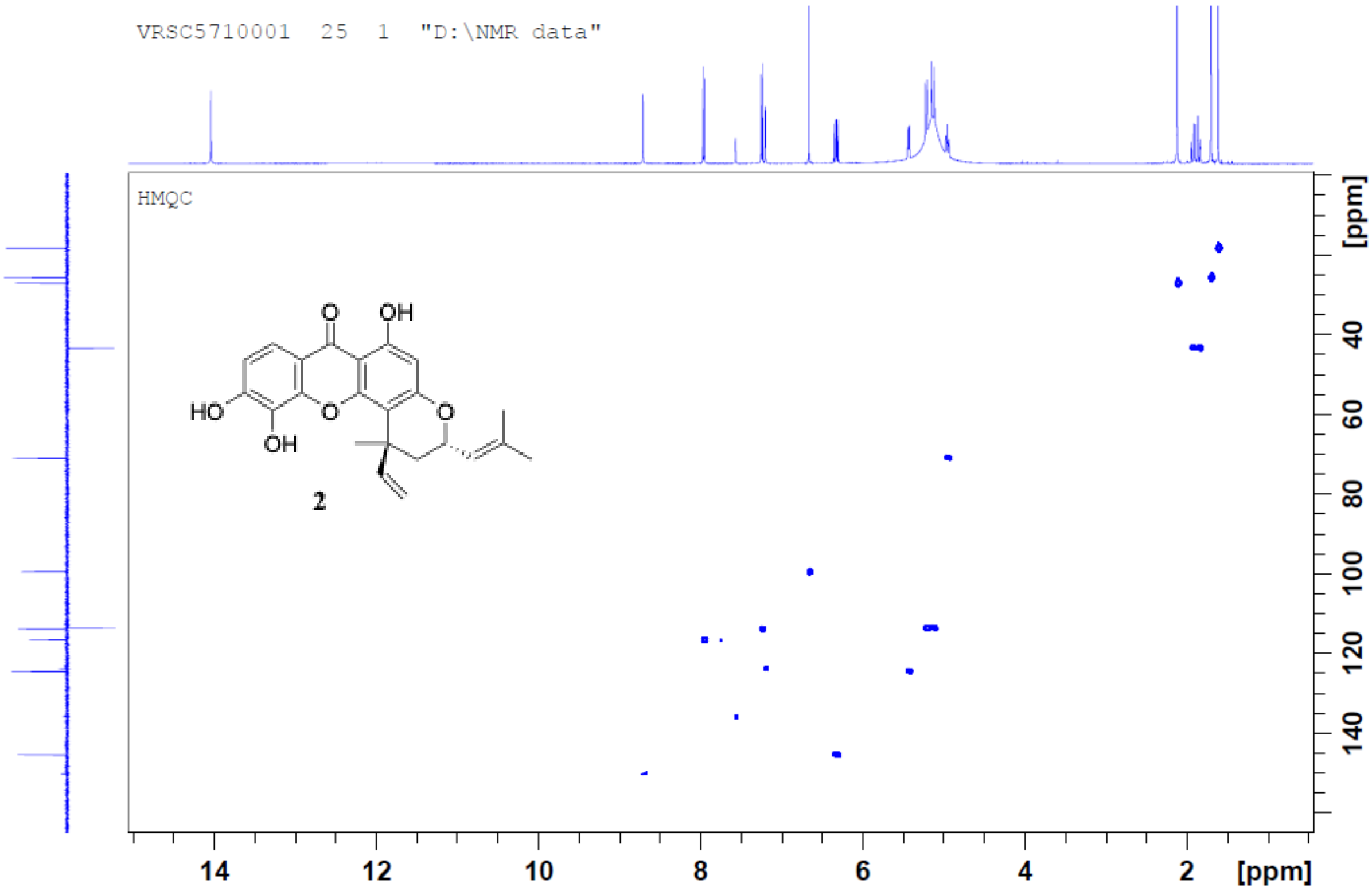

Figure S12. HMBC spectrum of compound **2** in pyridine-d_5

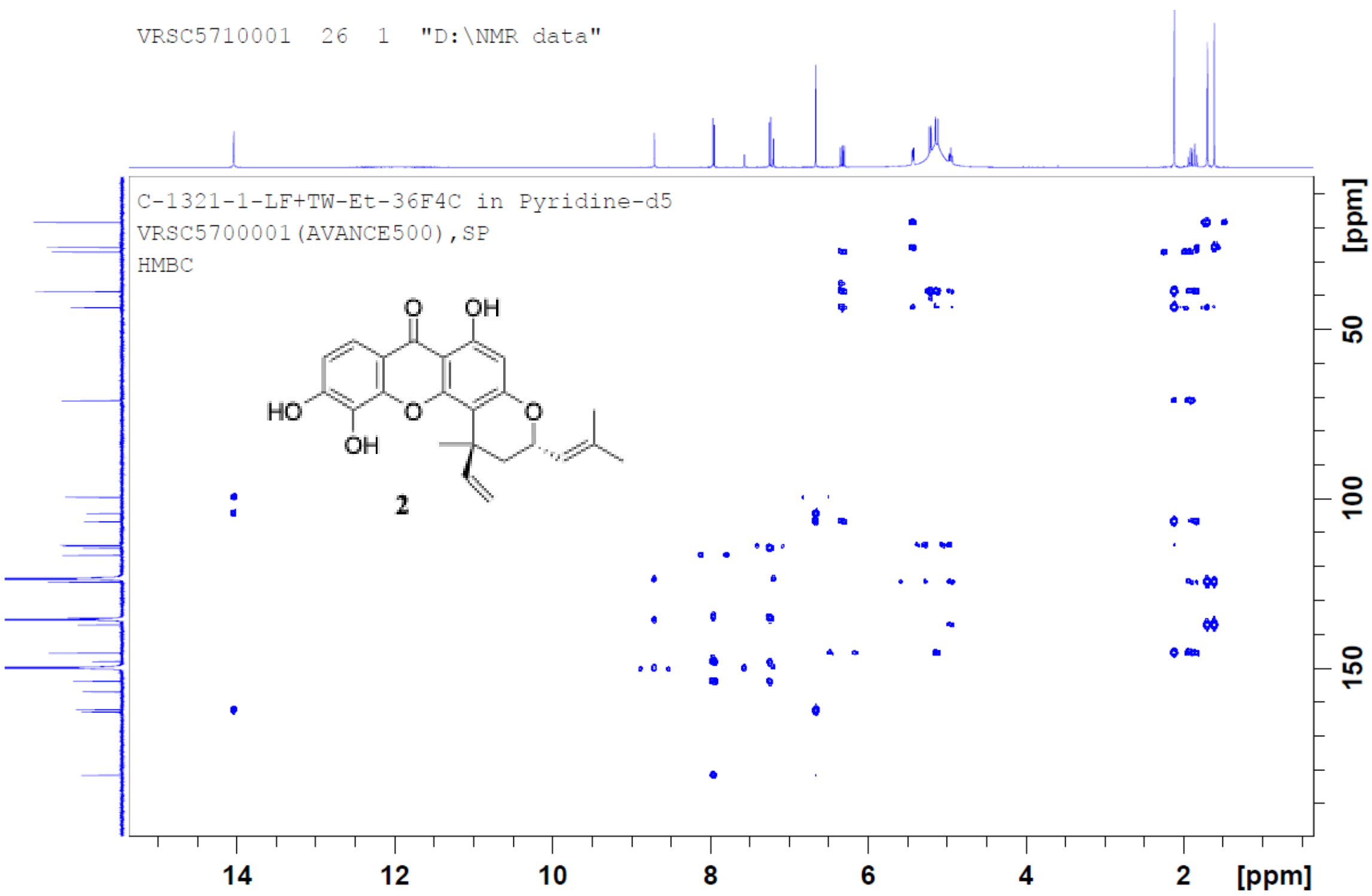

Figure S13. NOESY spectrum of compound **2** in pyridine-d_5

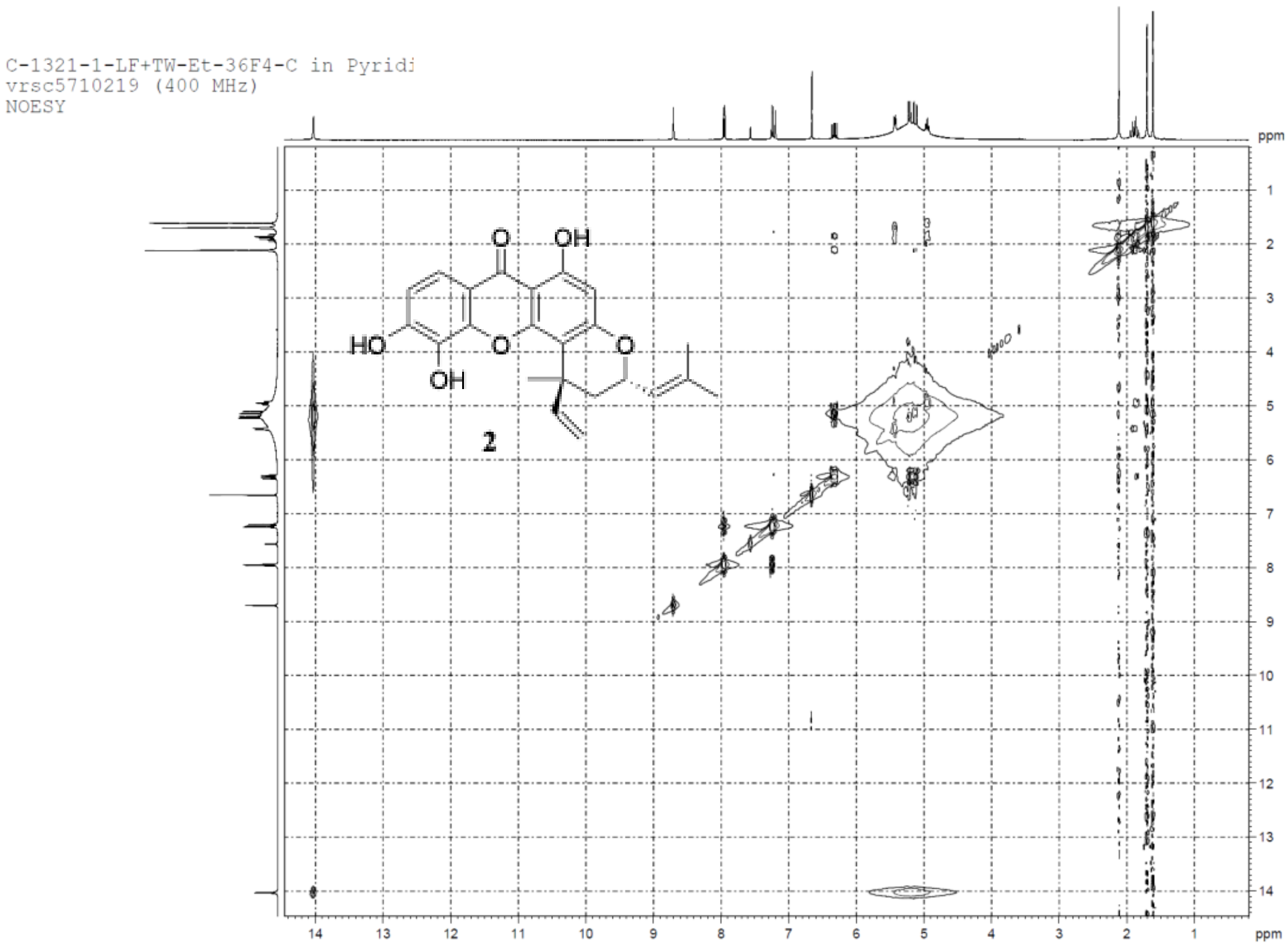

Figure S14. 500 MHz ^{1}H NMR spectrum of compound **2** in acetone-d_6

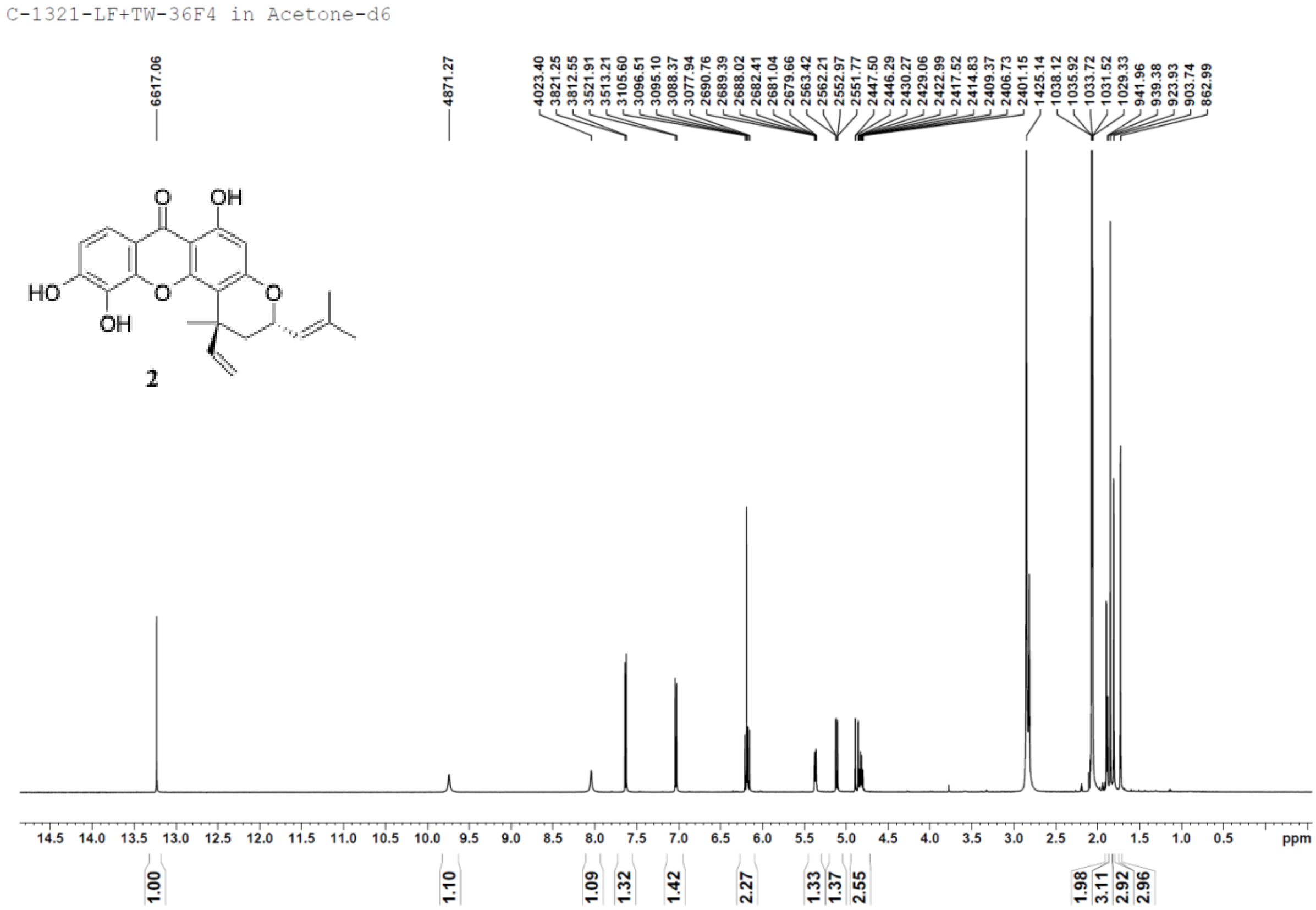

Figure S15. 125 MHz ^{13}C NMR spectrum of compound **2** in acetone-d_6

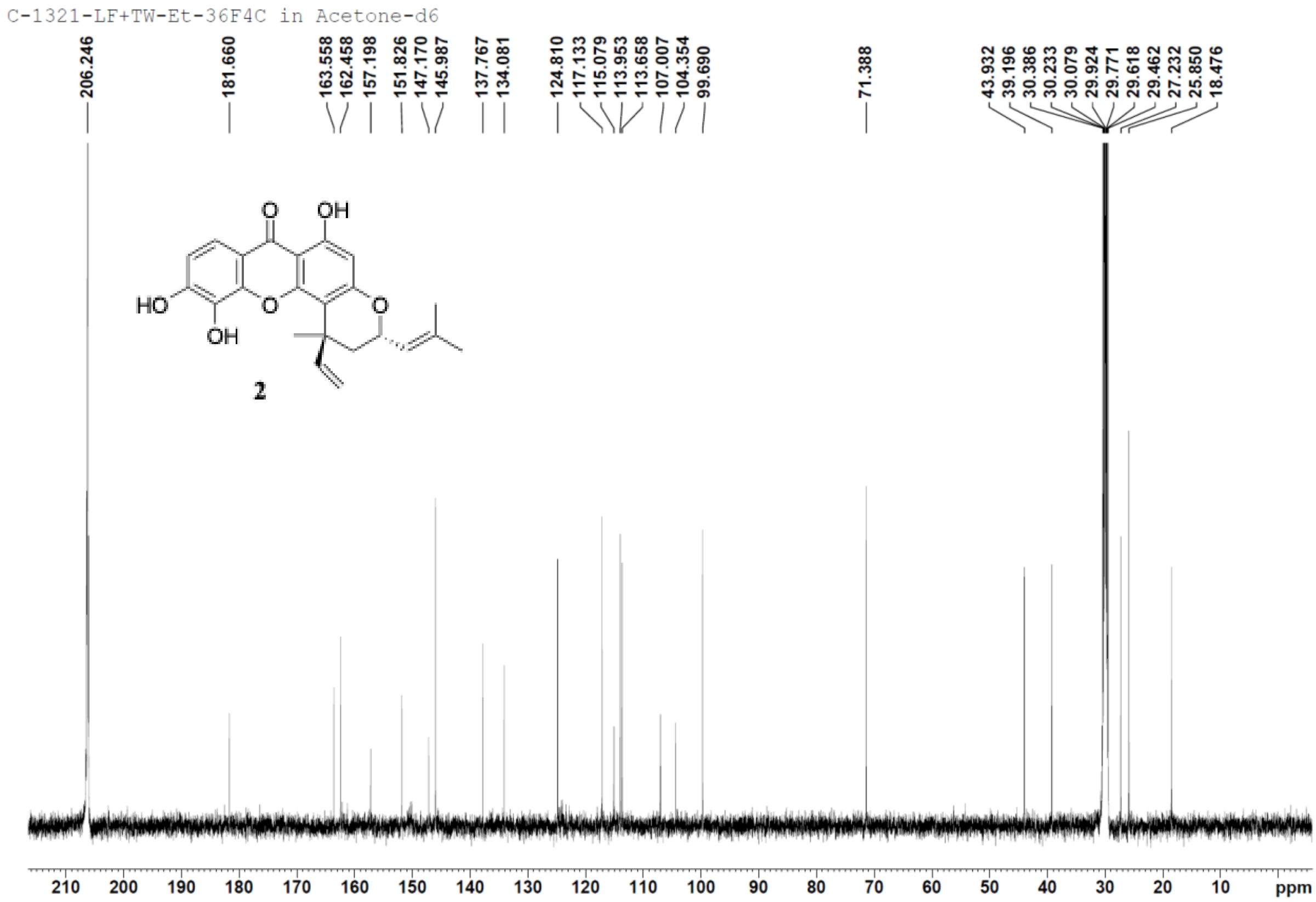

Figure S16. ^{1}H-^{1}H COSY spectrum of compound **2** in acetone-d_6

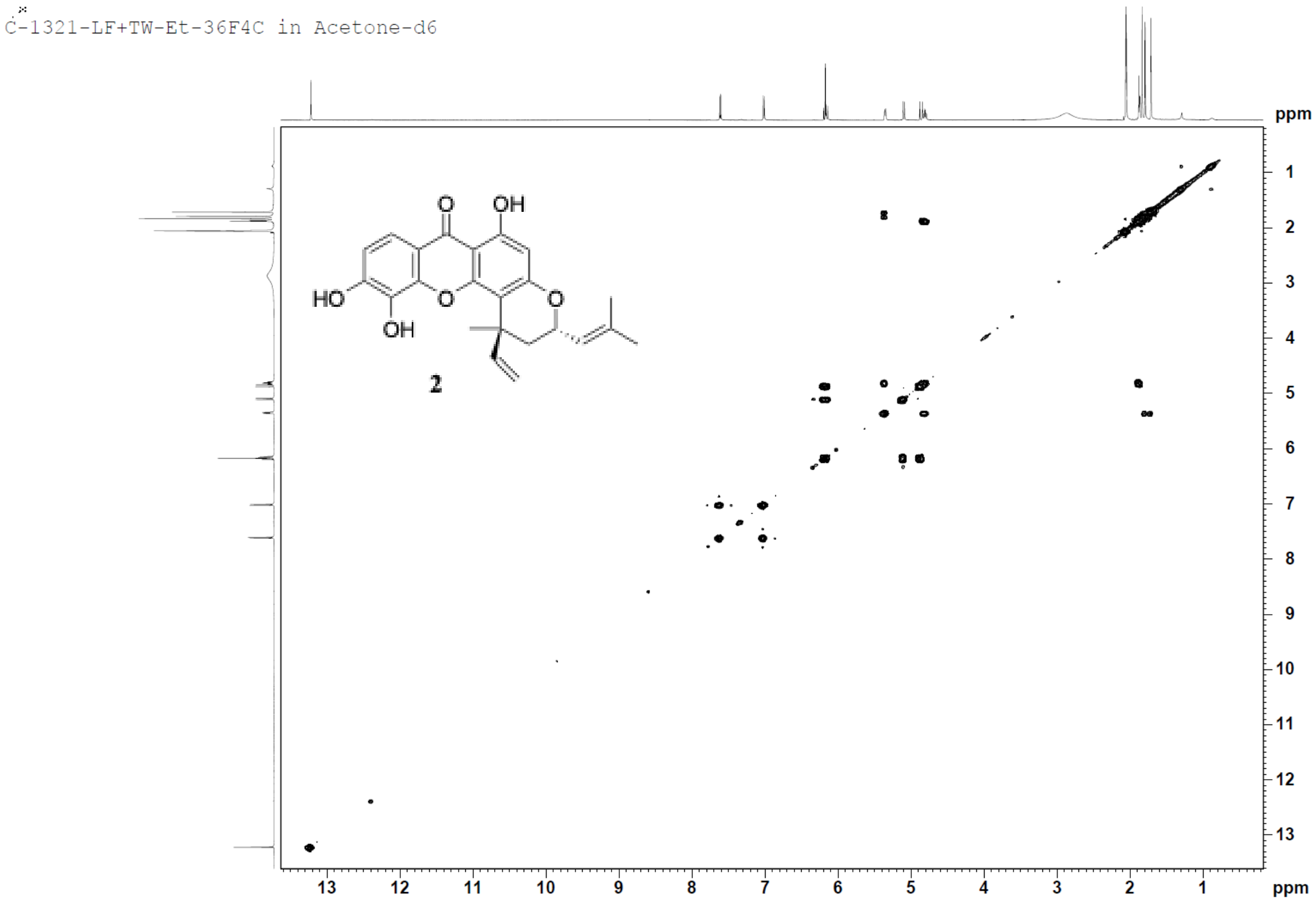

Figure S17. HMQC spectrum of compound **2** in acetone-d_6

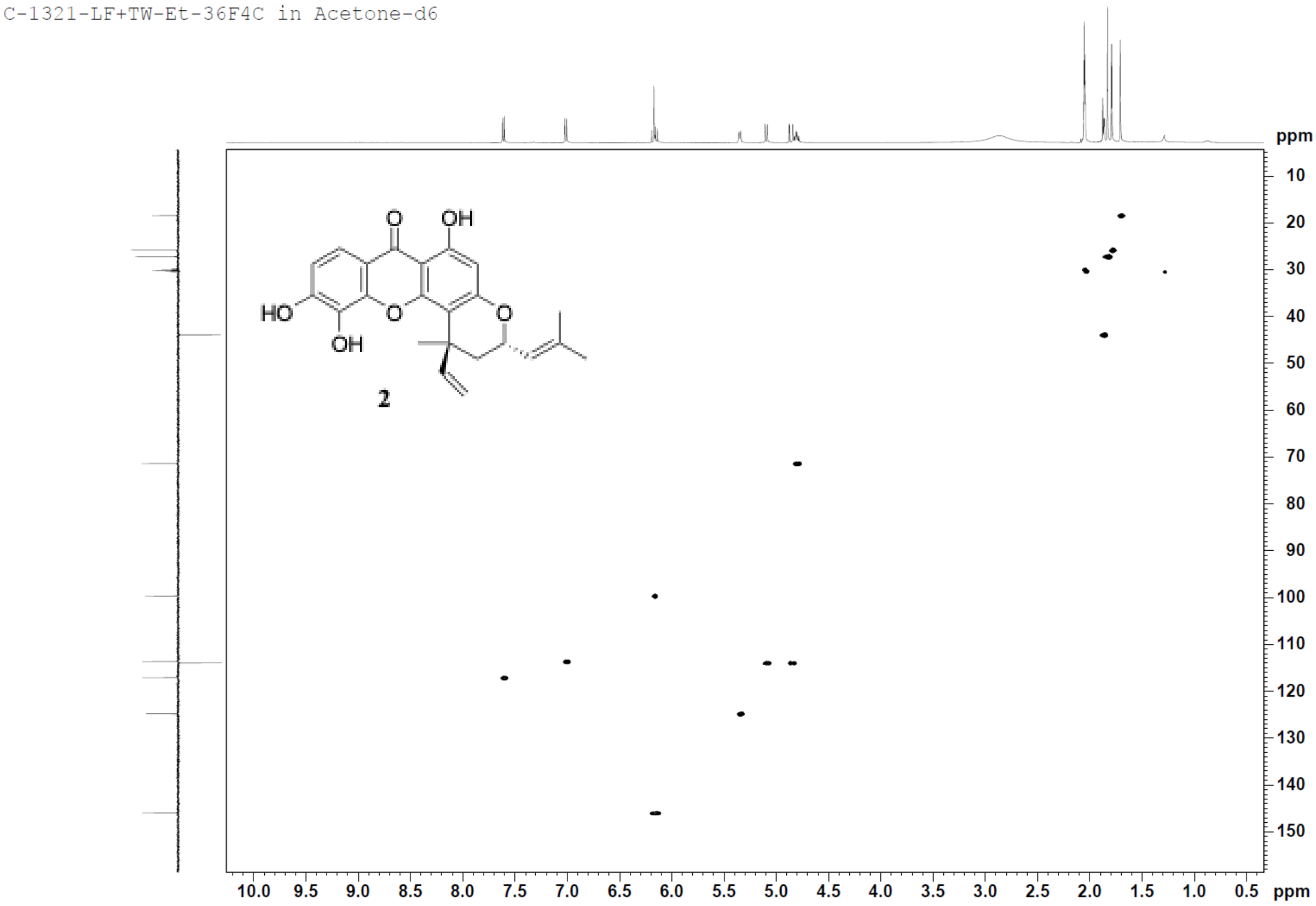

Figure S18. HMBC spectrum of compound **2** in acetone-d_6

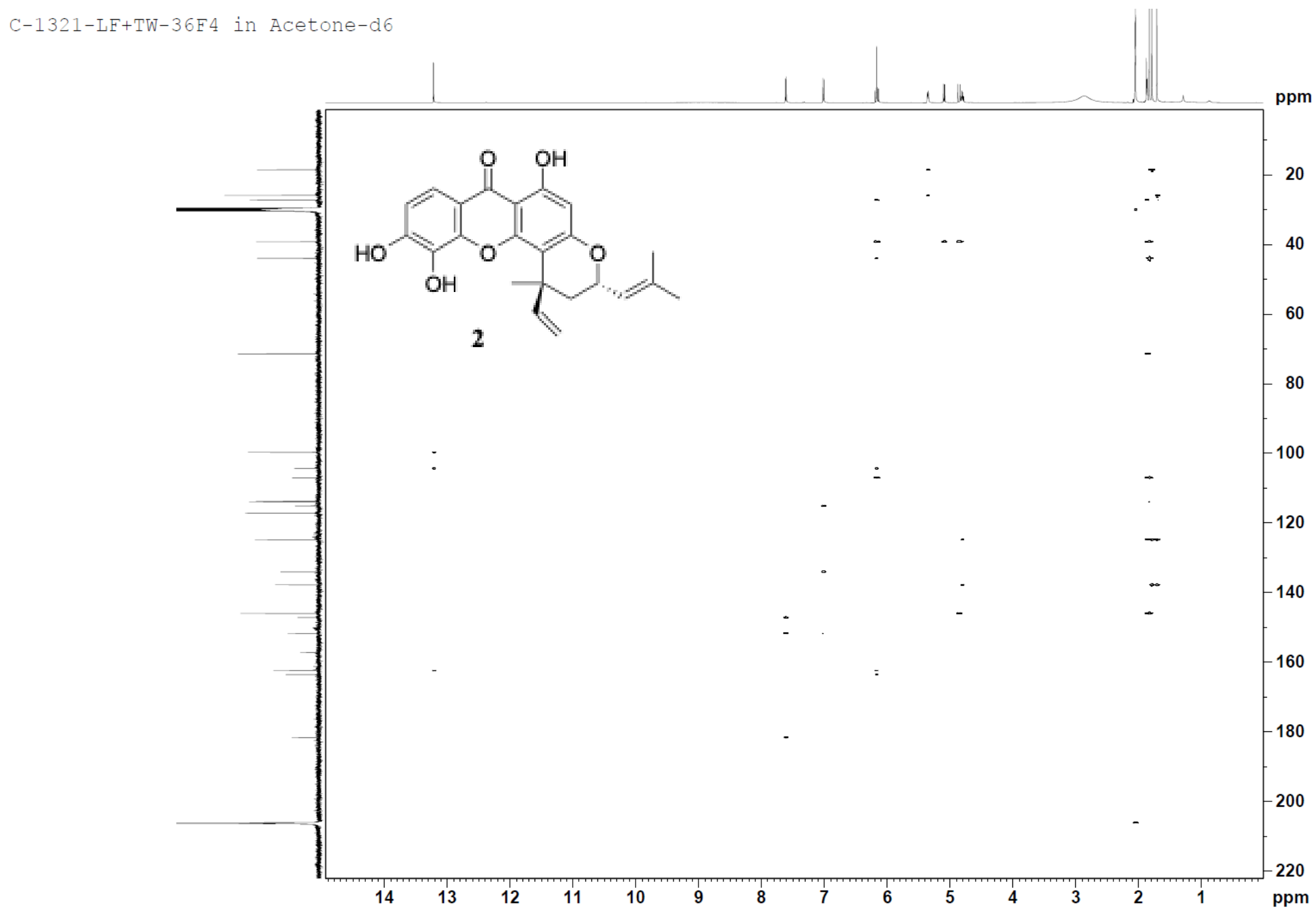

Figure S19. NOESY spectrum of compound **2** in acetone-d_6

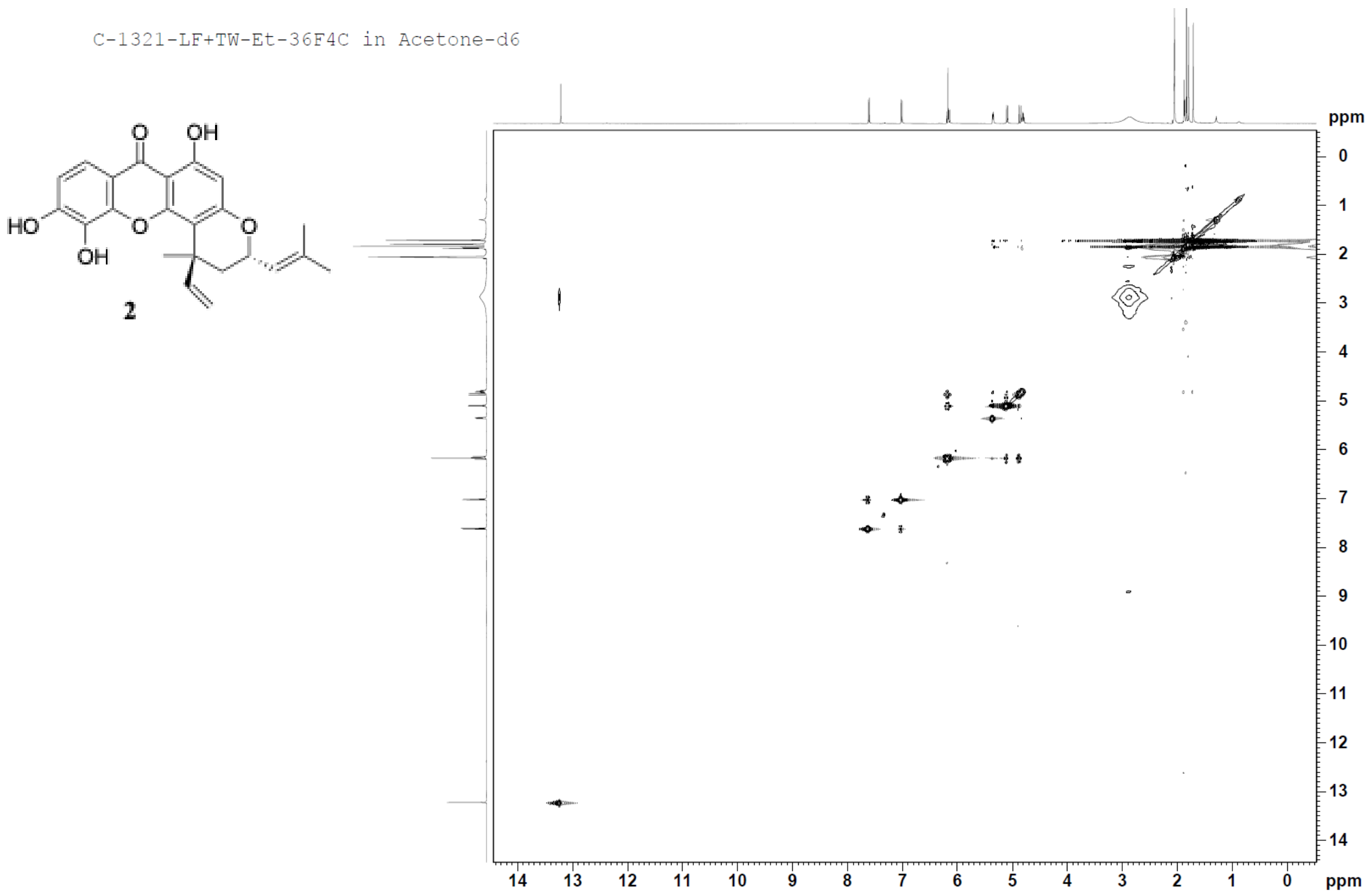

Figure S20. HRMS spectrum of compound **2**

Mass Spectrum List Report

Analysis Info

Analysis Name	VRSC570924001.d	Acquisition Date	9/24/2014 12:09:35 PM
Method	MKE_tune_wide_20130204.m	Operator	Administrator
Sample Name	C-1321-1-LF+TW-Et-36F4	Instrument	micrOTOF 72
	C-1321-1-LF+TW-Et-36F4		

Acquisition Parameter

Source Type	ESI	Ion Polarity	Positive	Set Corrector Fill	79 V
Scan Range	n/a	Capillary Exit	200.0 V	Set Pulsar Pull	406 V
Scan Begin	50 m/z	Hexapole RF	400.0 V	Set Pulsar Push	388 V
Scan End	3000 m/z	Skimmer 1	45.0 V	Set Reflector	1300 V
		Hexapole 1	25.0 V	Set Flight Tube	9000 V
				Set Detector TOF	1910 V

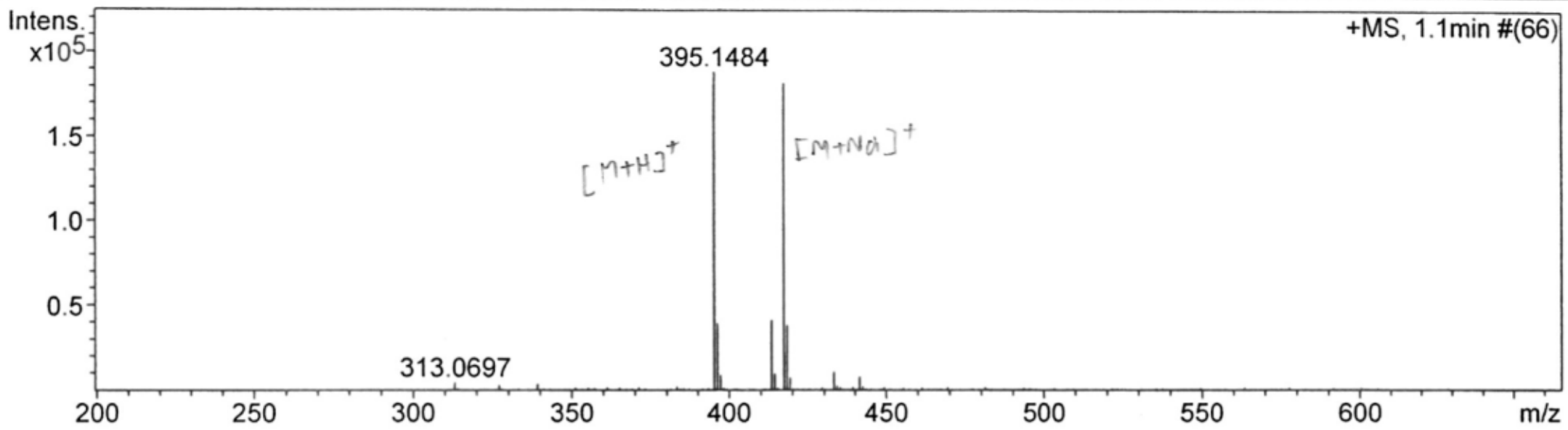

Figure S21. 500 MHz ^{1}H NMR spectrum of compound **3** in pyridine-d_5

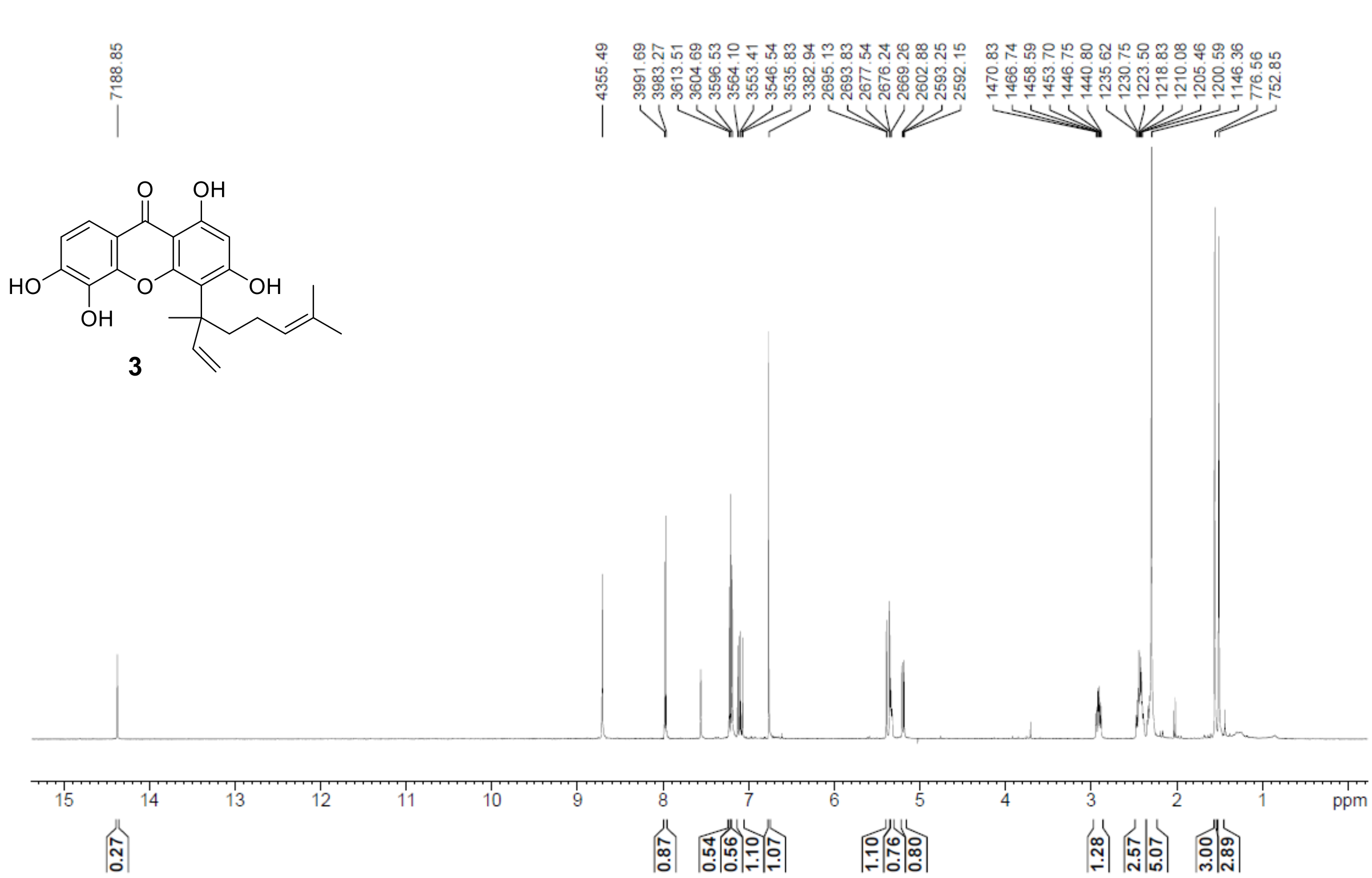

Figure S22. 125 MHz ^{13}C NMR spectrum of compound **3** in pyridine-d_5

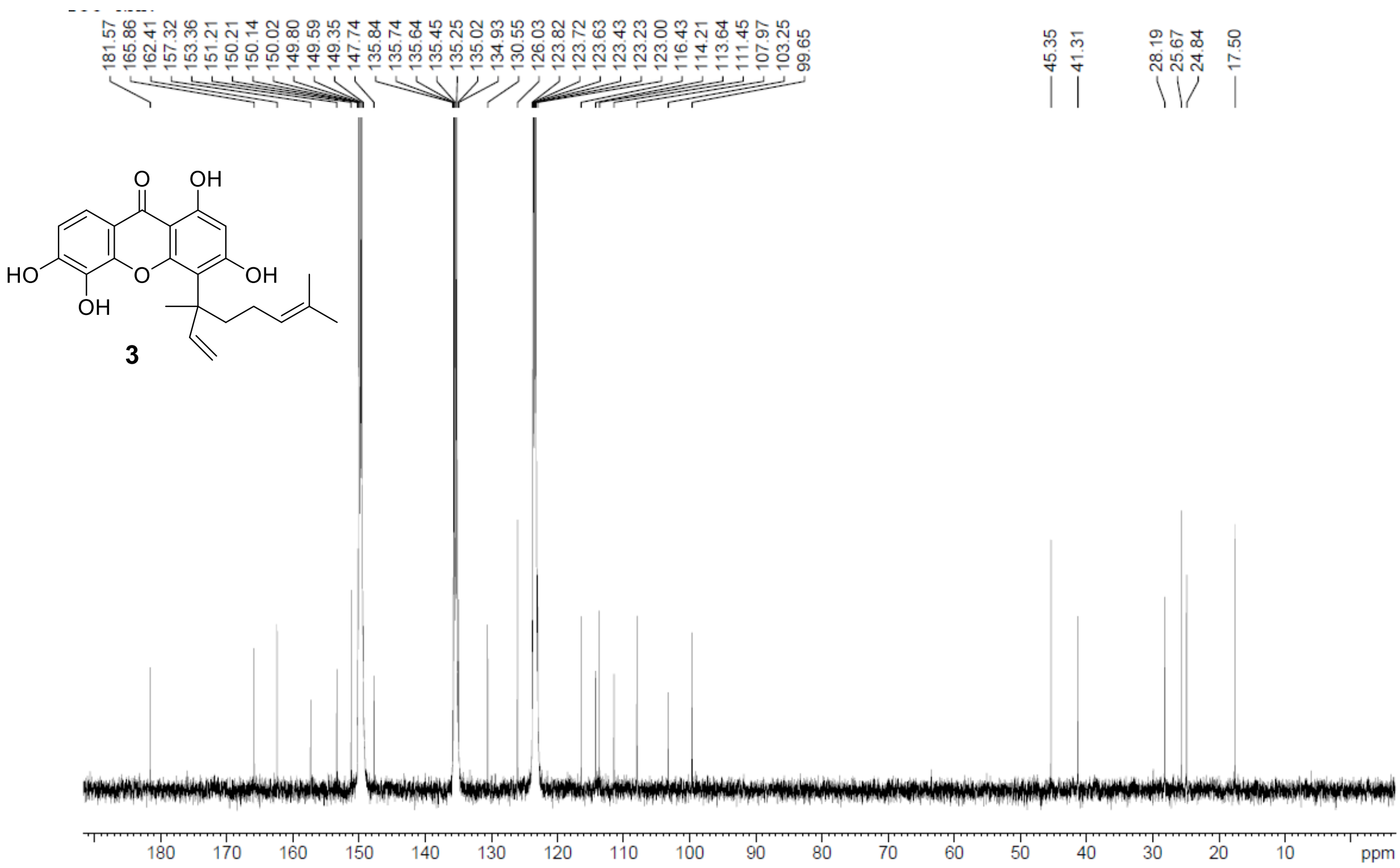

Figure S23. ^{1}H-^{1}H COSY spectrum of compound **3** in pyridine-d_5

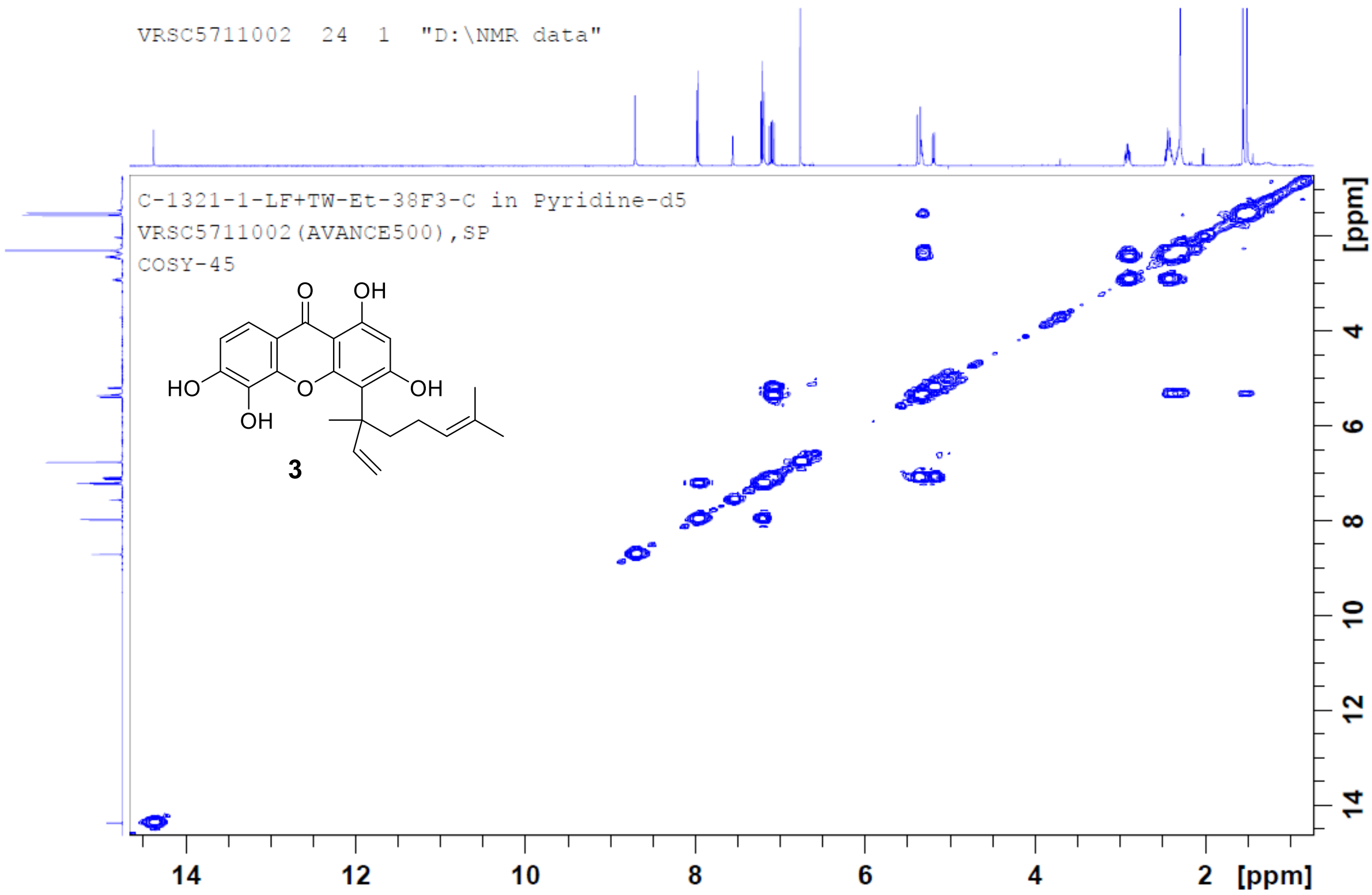

Figure S24. HMQC spectrum of compound **3** in pyridine-d_5

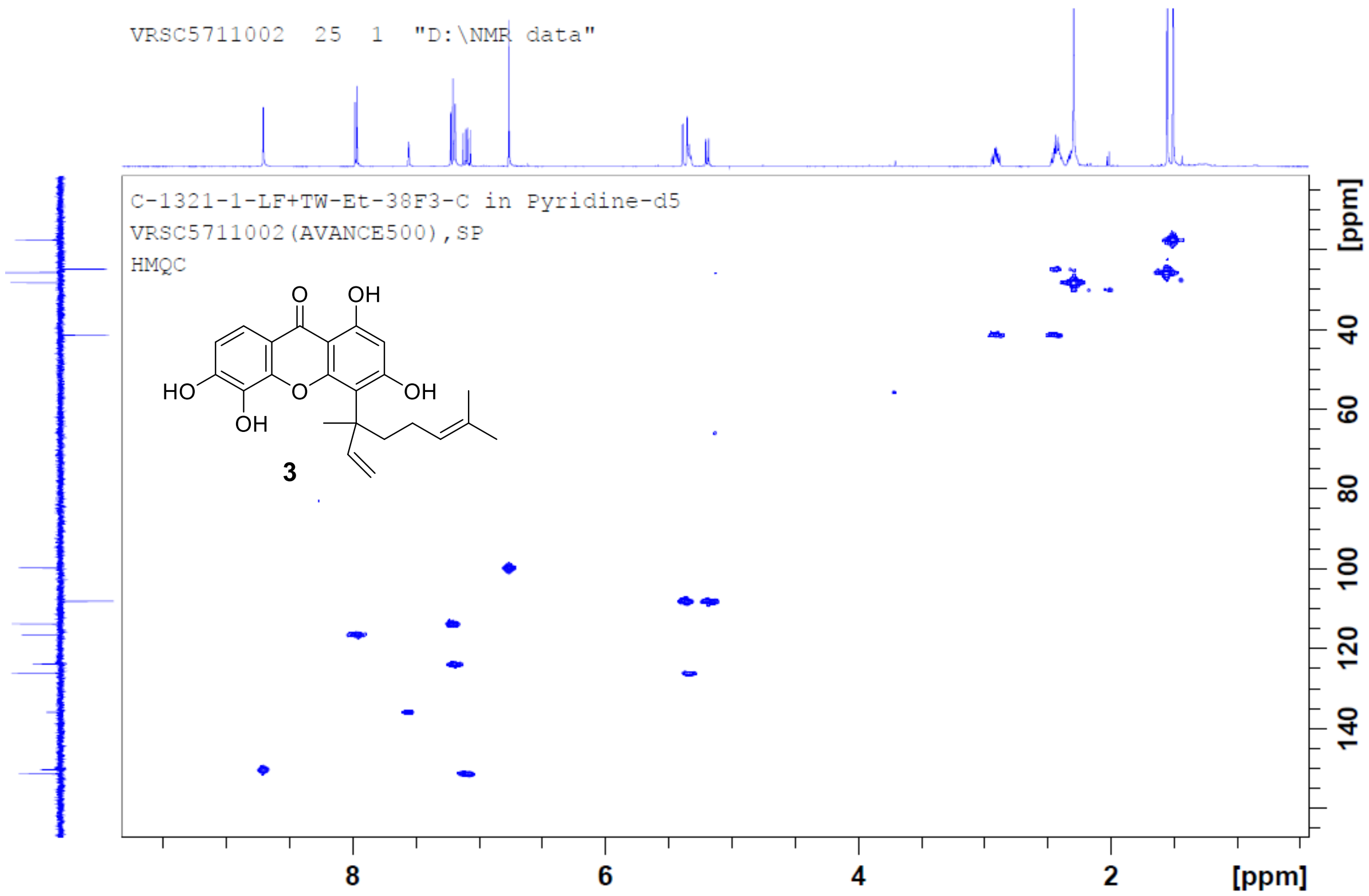

Figure S25. HMBC spectrum of compound **3** in pyridine-d_5

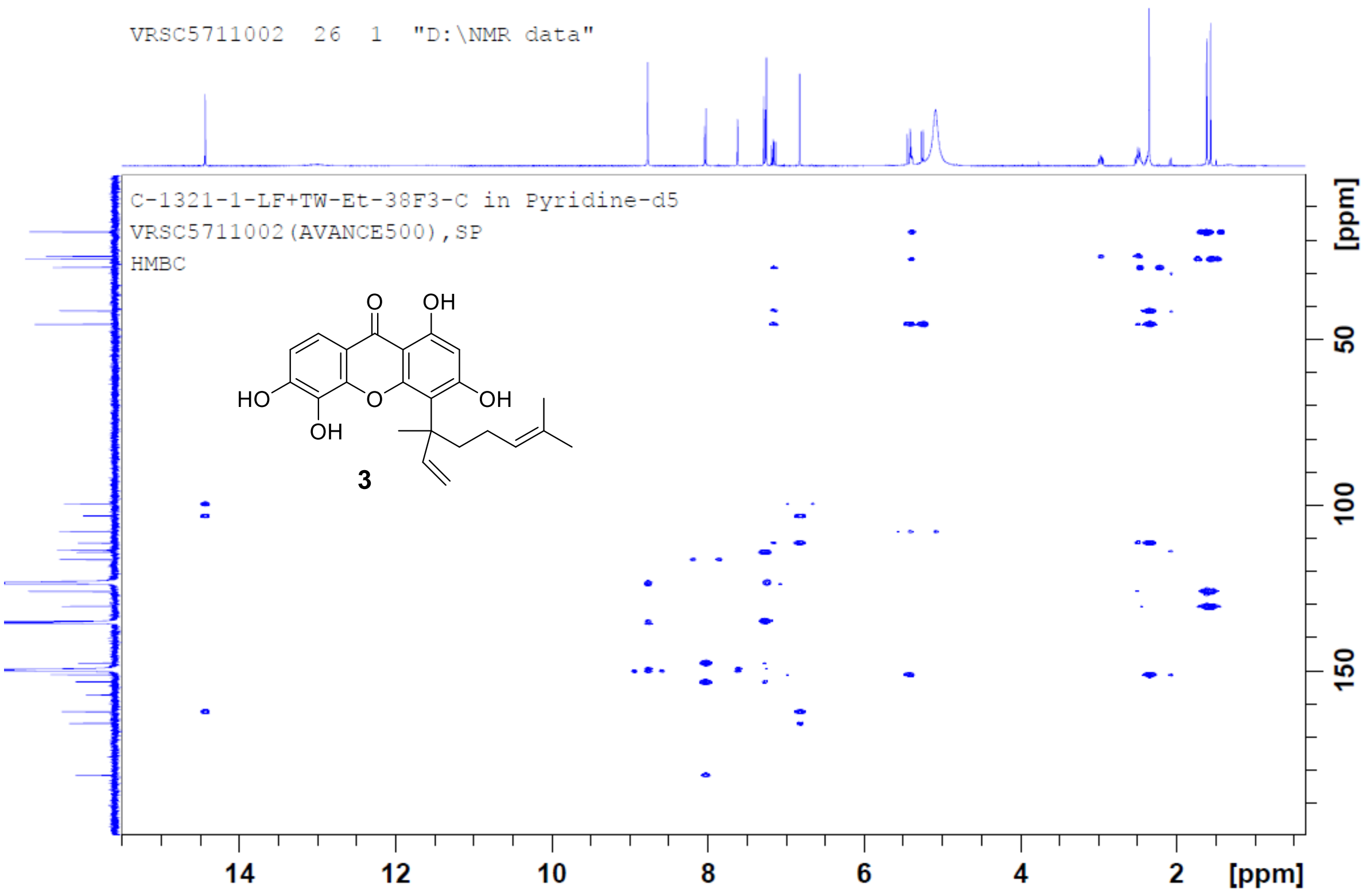

Figure S26. NOESY spectrum of compound **3** in pyridine-d_5

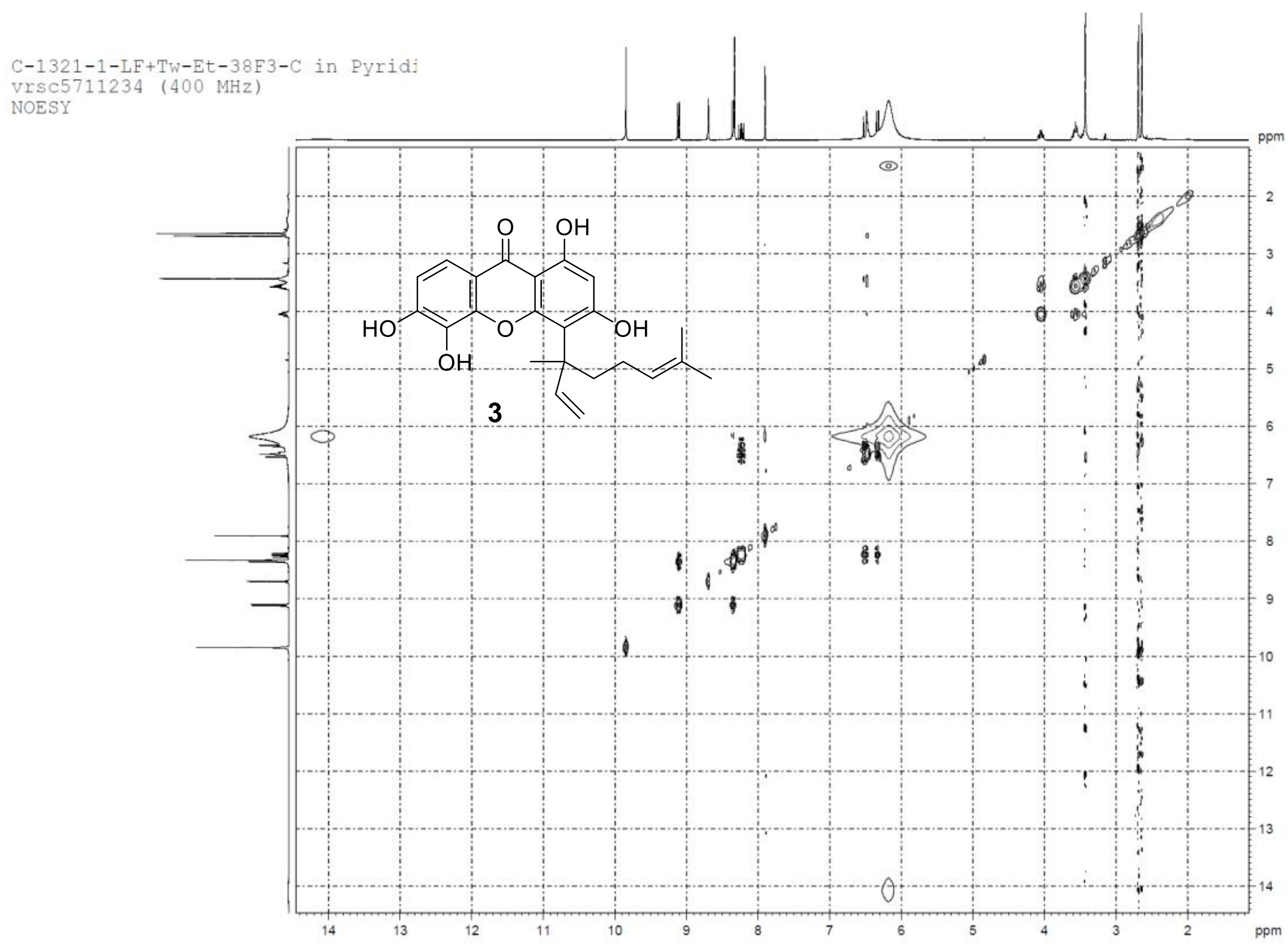

Figure S27. HRMS spectrum of compound **3**

Mass Spectrum List Report

Analysis Info

Analysis Name	VRSC571027002.d	Acquisition Date	10/27/2014 12:25:38 PM
Method	MKE_tune_wide_20130204.m	Operator	Administrator
Sample Name	C-1321-1-LF+TW-Et-38F3C	Instrument	micrOTOF 72
	C-1321-1-LF+TW-Et-38F3C		

Acquisition Parameter

Source Type	ESI	Ion Polarity	Positive	Set Corrector Fill	79 V
Scan Range	n/a	Capillary Exit	230.0 V	Set Pulsar Pull	406 V
Scan Begin	50 m/z	Hexapole RF	400.0 V	Set Pulsar Push	388 V
Scan End	3000 m/z	Skimmer 1	45.0 V	Set Reflector	1300 V
		Hexapole 1	25.0 V	Set Flight Tube	9000 V
				Set Detector TOF	1910 V

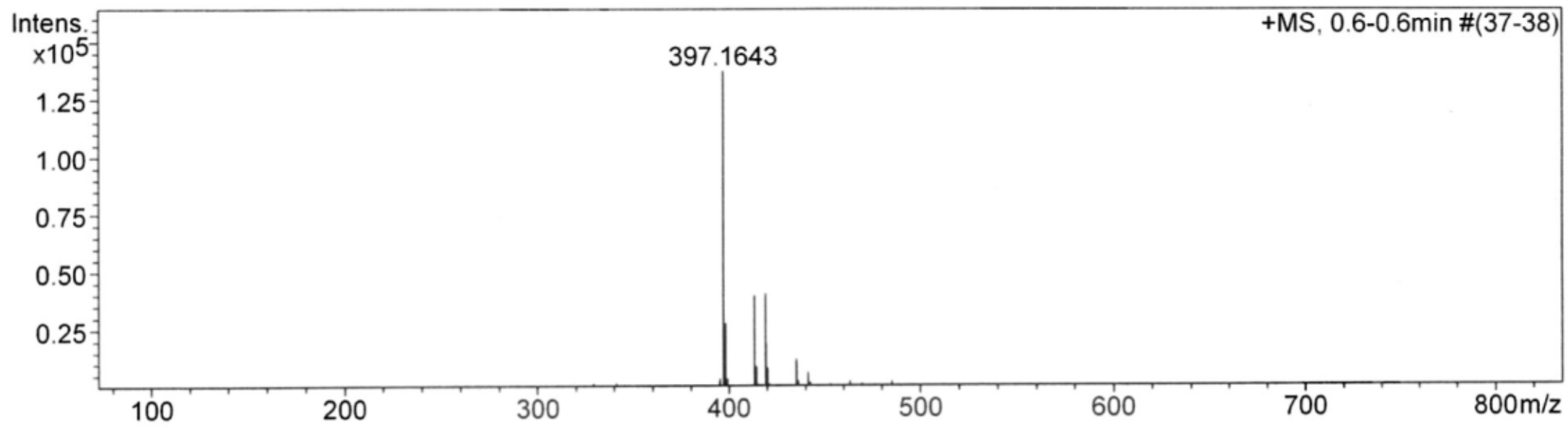

Figure S28. 500 MHz ^{1}H NMR spectrum of compound **4** in DMSO-d_6

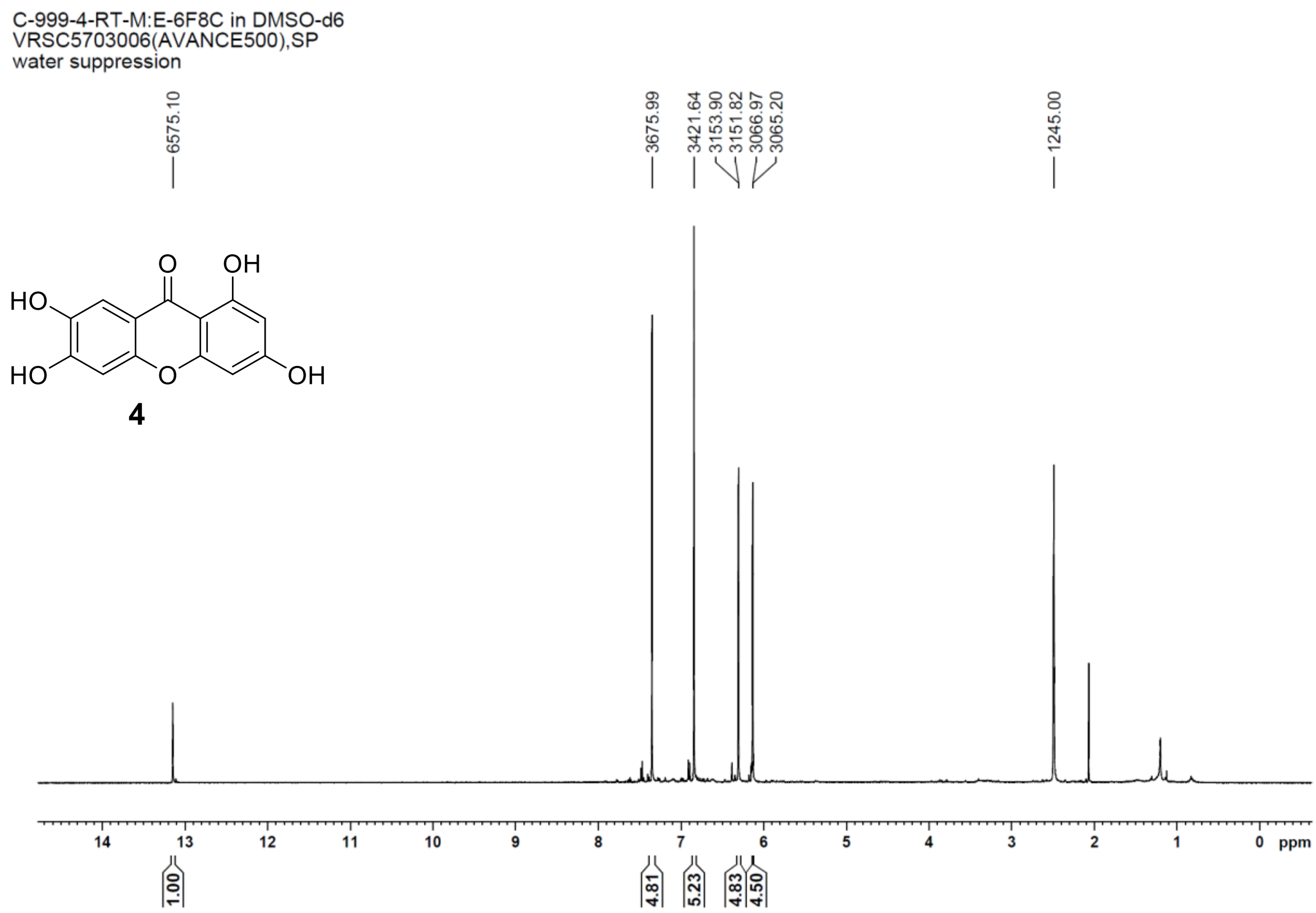

Figure S29. 125 MHz ^{13}C NMR spectrum of compound **4** in DMSO-d_6

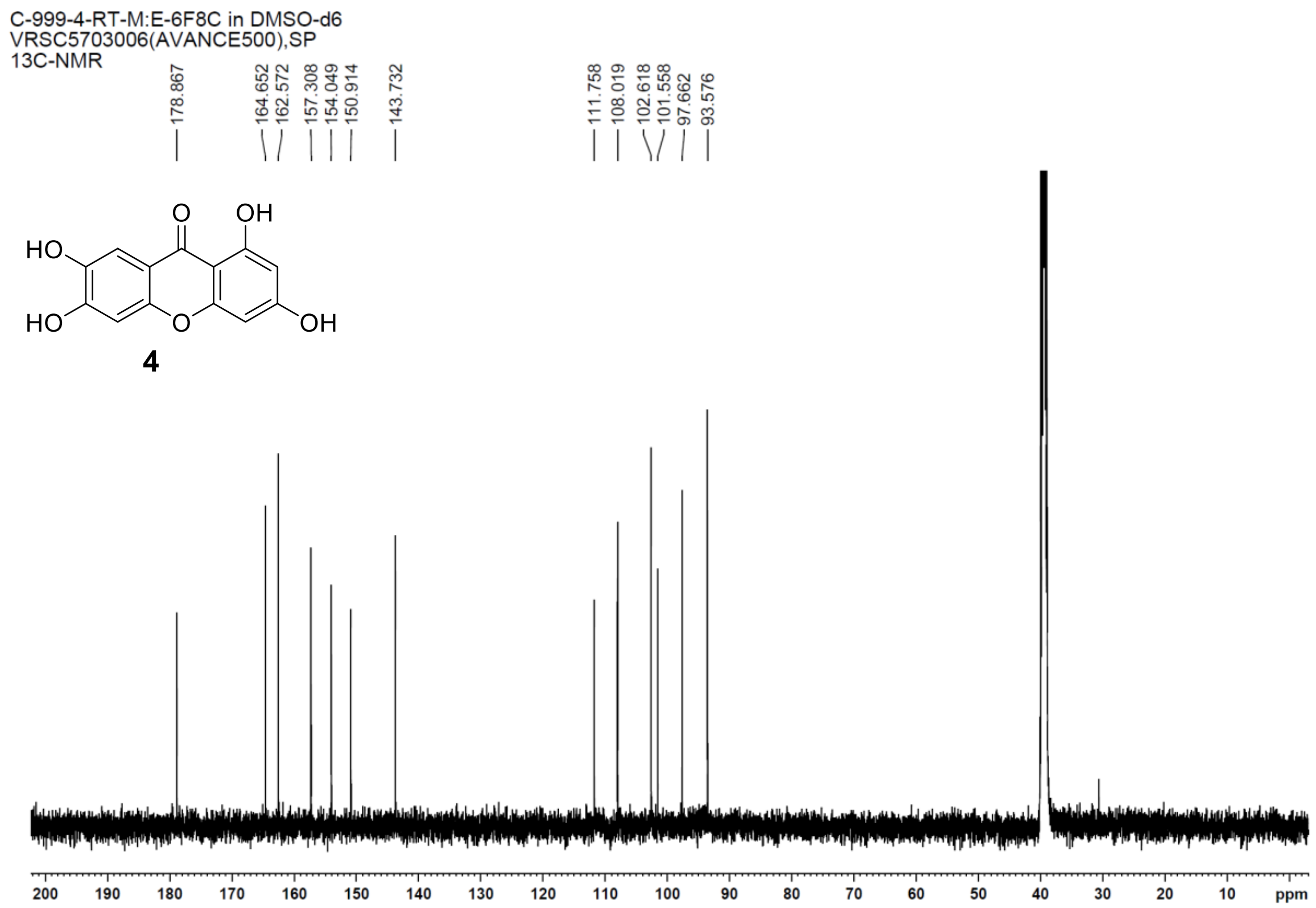

Figure S30. HRMS spectrum of compound **4**

Mass Spectrum List Report

Analysis Info

Analysis Name	VRSC570401002.d	Acquisition Date	4/1/2014 6:19:14 PM
Method	MKE_tune_low_positive_20130204.m	Operator	Administrator
Sample Name	C-999-4-RT-M:E-6F8-C	Instrument	micrOTOF 72
	C-999-4-RT-M:E-6F8-C		

Acquisition Parameter

				Set Corrector Fill	79 V
Source Type	ESI	Ion Polarity	Positive	Set Pulsar Pull	406 V
Scan Range	n/a	Capillary Exit	110.0 V	Set Pulsar Push	388 V
Scan Begin	50 m/z	Hexapole RF	90.0 V	Set Reflector	1300 V
Scan End	3000 m/z	Skimmer 1	45.5 V	Set Flight Tube	9000 V
		Hexapole 1	25.0 V	Set Detector TOF	1910 V

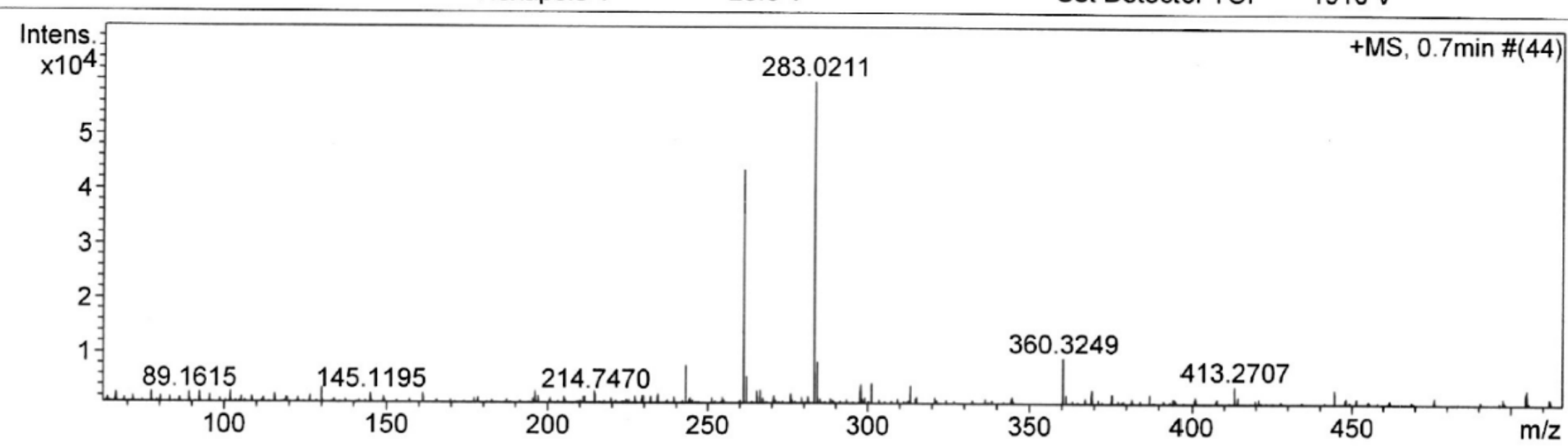

Figure S31. 400 MHz ^{1}H NMR spectrum of compound **5** in acetone-d_6

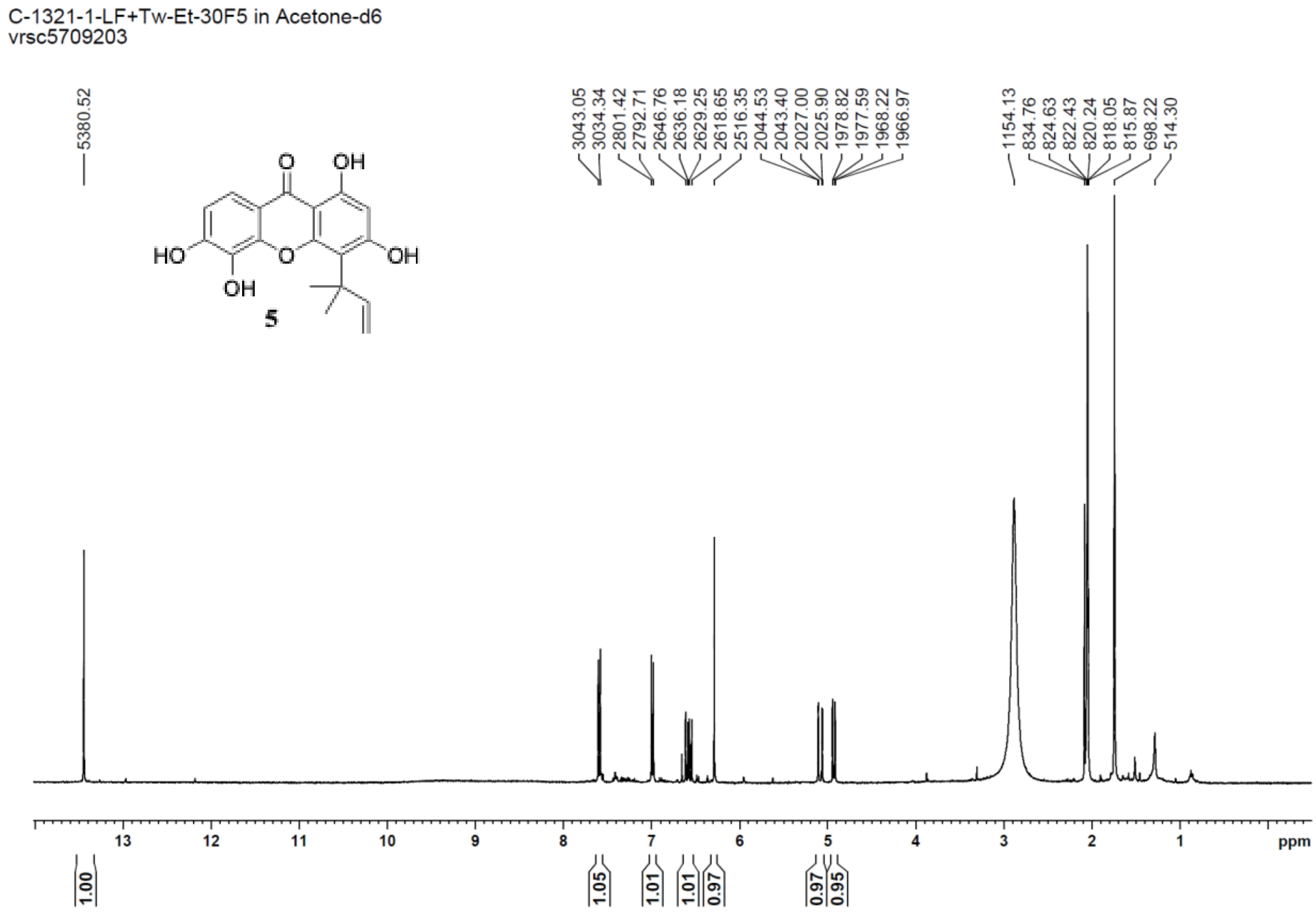

Figure S32. 100 MHz ^{13}C NMR spectrum of compound **5** in acetone-d_6

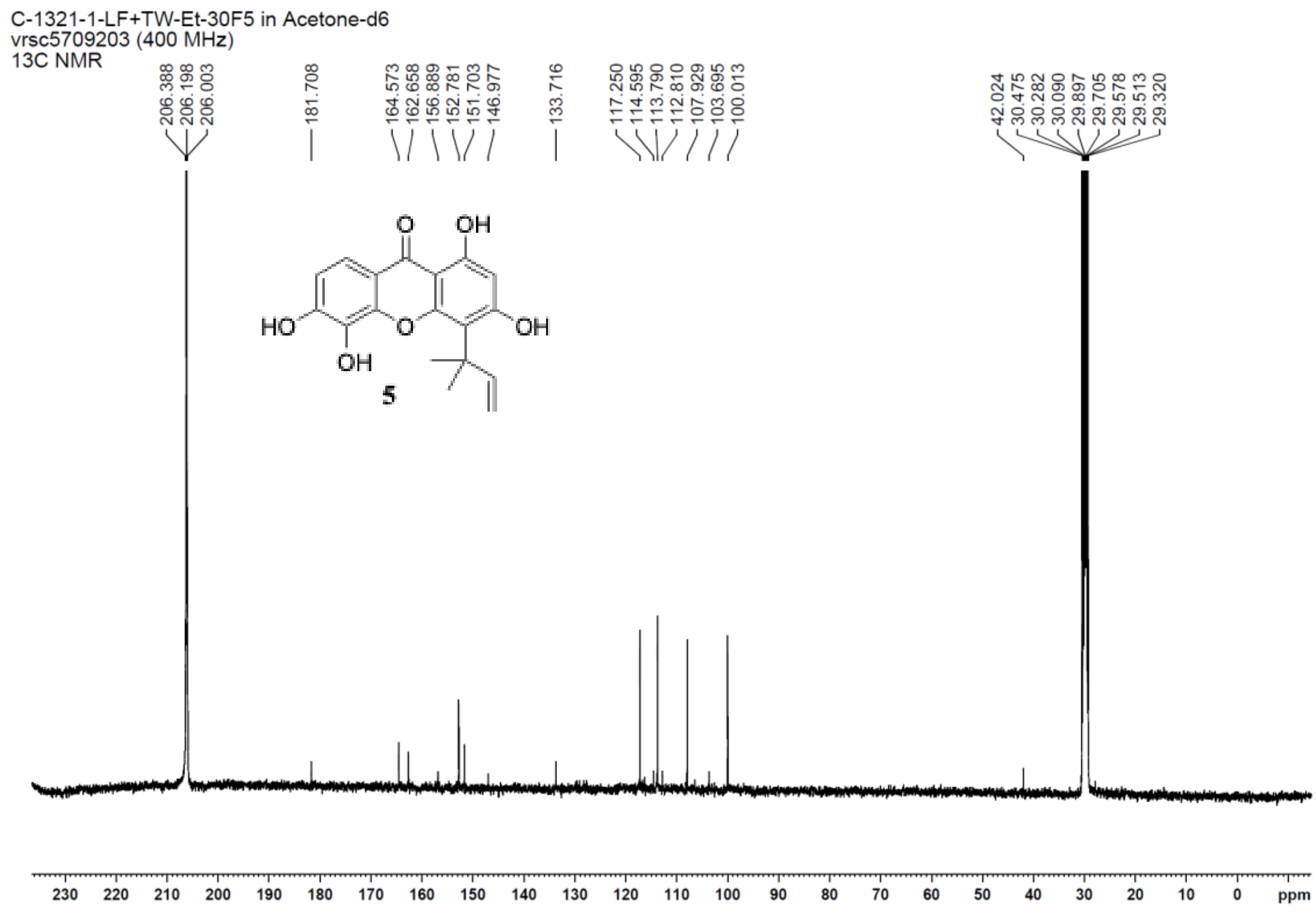

Figure S33. HRMS spectrum of compound **5**

Mass Spectrum List Report

Analysis Info

Analysis Name	VRSC571015002.d	Acquisition Date	10/15/2014 9:19:45 AM
Method	Tune_wide_POS_Natee20130403.m	Operator	Administrator
Sample Name	C-1321-1-LF+TW-Et-30F5	Instrument	micrOTOF 72
	C-1321-1-LF+TW-Et-30F5		

Acquisition Parameter

Source Type	ESI	Ion Polarity	Positive	Set Corrector Fill	79 V
Scan Range	n/a	Capillary Exit	120.0 V	Set Pulsar Pull	406 V
Scan Begin	50 m/z	Hexapole RF	200.0 V	Set Pulsar Push	388 V
Scan End	3000 m/z	Skimmer 1	45.0 V	Set Reflector	1300 V
		Hexapole 1	24.3 V	Set Flight Tube	9000 V
				Set Detector TOF	1910 V

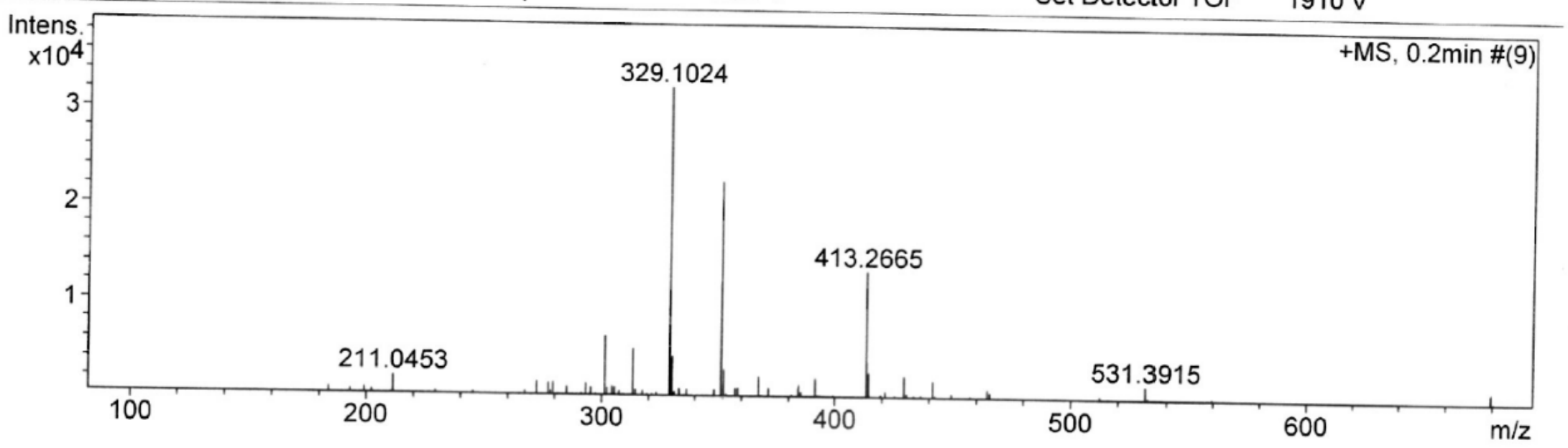

Figure S34. 500 MHz ^{1}H NMR spectrum of compound **6** in DMSO-d_6

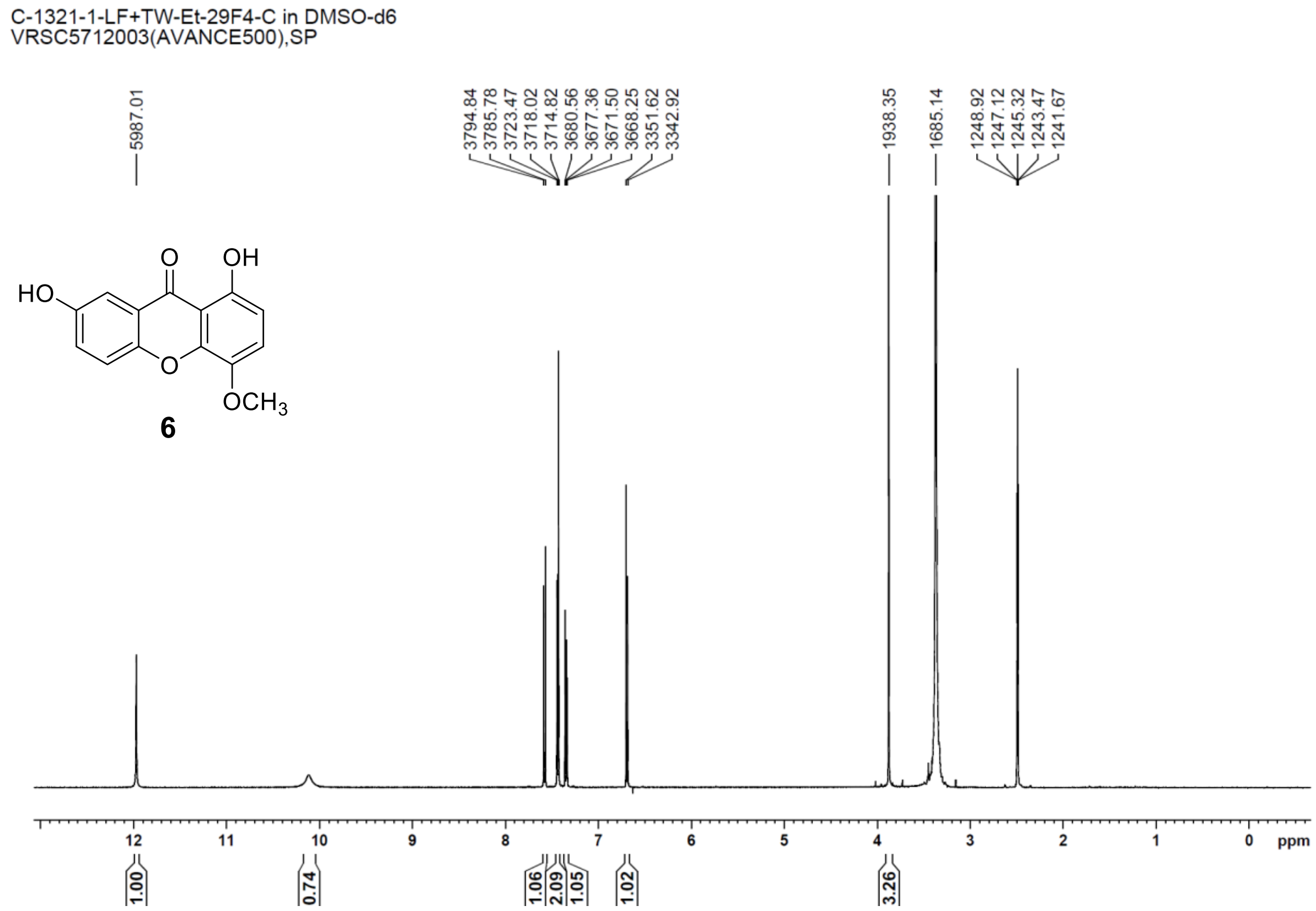

Figure S35. 125 MHz ^{13}C NMR spectrum of compound **6** in DMSO-d_6

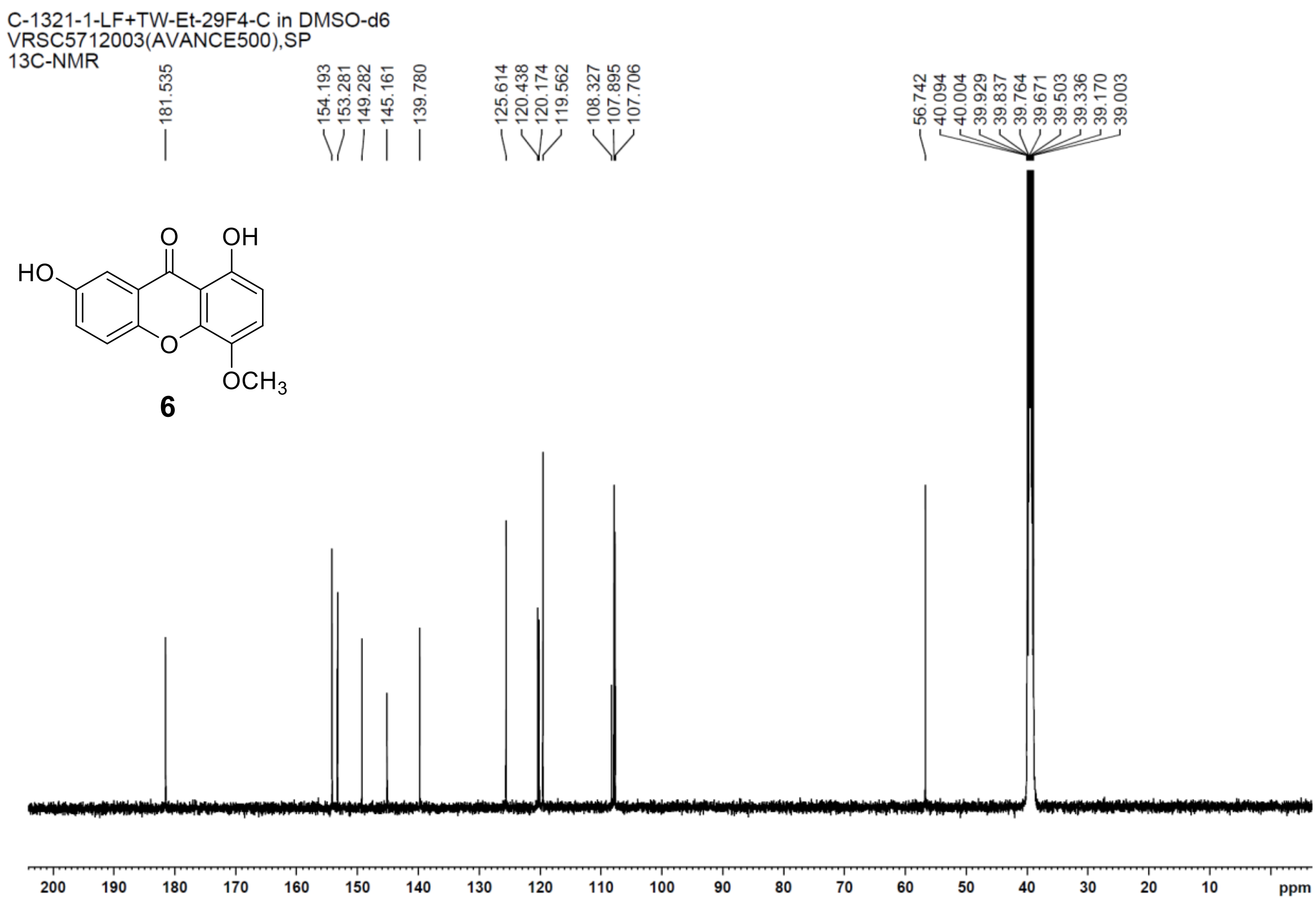

Figure S36. HRMS spectrum of compound **6**

Mass Spectrum List Report

Analysis Info

Analysis Name	VRSC5710150011.d	Acquisition Date	10/15/2014 9:15:29 AM
Method	MKE_tune_low_positive_20130204.m	Operator	Administrator
Sample Name	C-1321-1-LF+TW-Et-29F4-C	Instrument	micrOTOF 72
	C-1321-1-LF+TW-Et-29F4-C		

Acquisition Parameter

Source Type	ESI	Ion Polarity	Positive	Set Corrector Fill	79 V
Scan Range	n/a	Capillary Exit	90.0 V	Set Pulsar Pull	406 V
Scan Begin	50 m/z	Hexapole RF	90.0 V	Set Pulsar Push	388 V
Scan End	3000 m/z	Skimmer 1	45.5 V	Set Reflector	1300 V
		Hexapole 1	25.0 V	Set Flight Tube	9000 V
				Set Detector TOF	1910 V

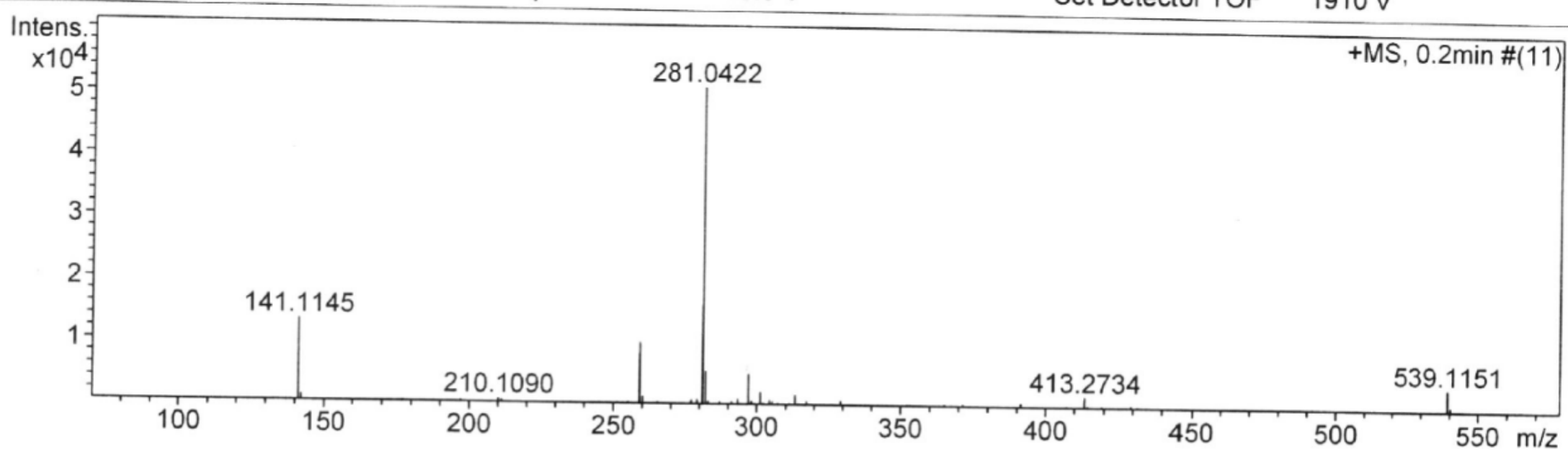

Figure S37. 500 MHz ^{1}H NMR spectrum of compound **7** in acetone-d_6

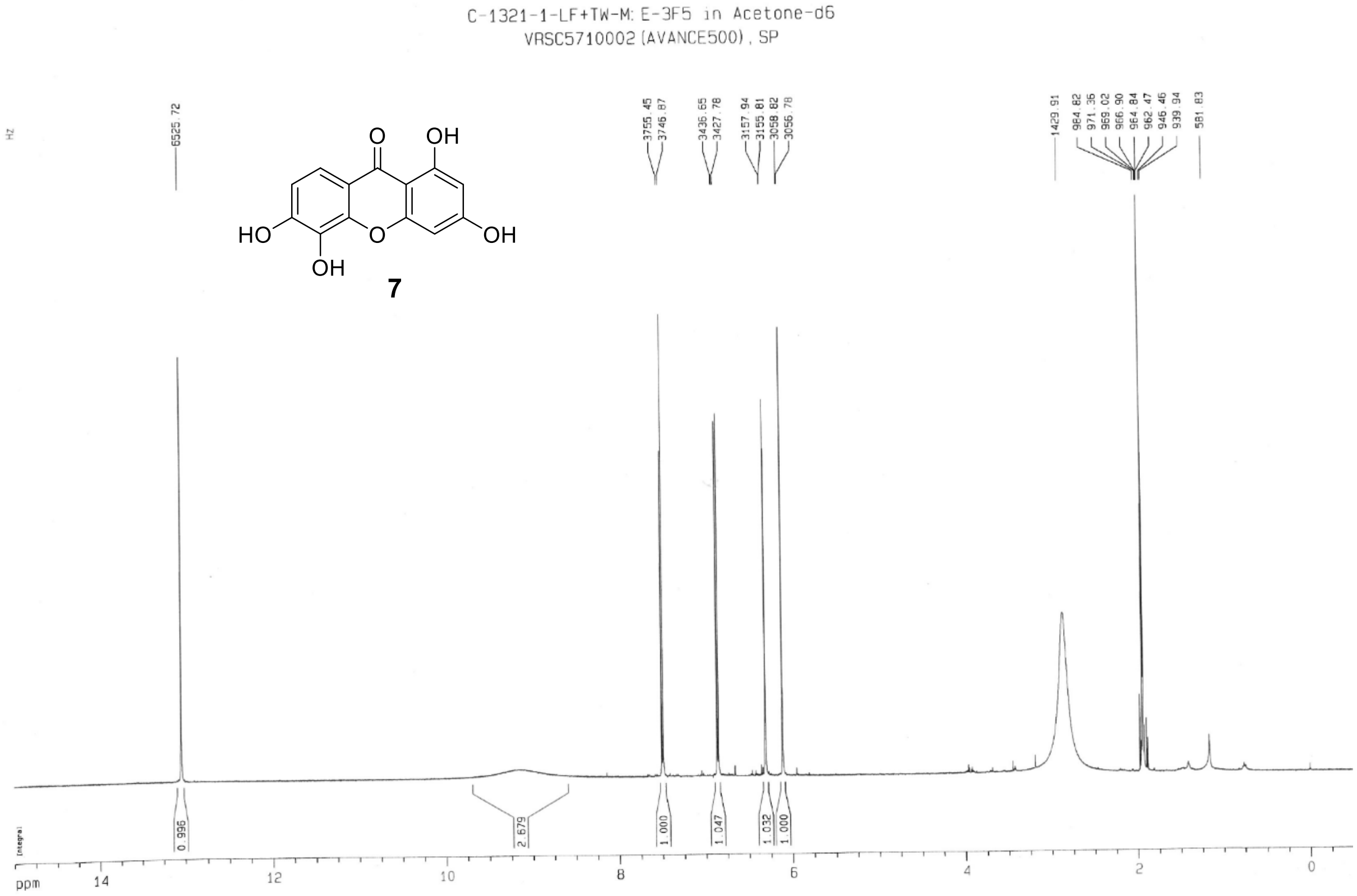

Figure S38. 125 MHz ^{13}C NMR spectrum of compound **7** in acetone-d_6

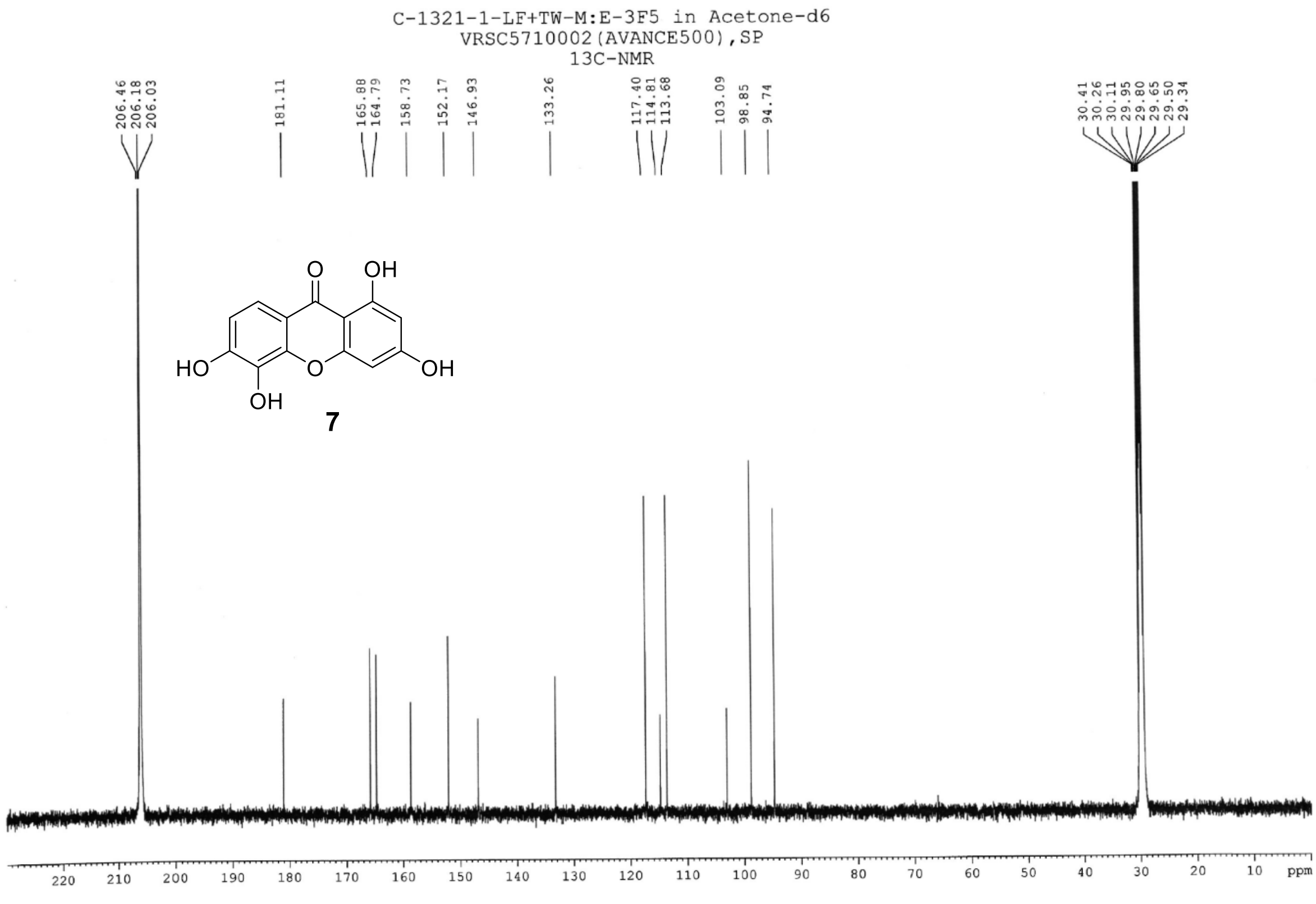

Figure S39. HRMS spectrum of compound **7**

Mass Spectrum List Report

Analysis Info

Analysis Name	VRSC571015006.d	Acquisition Date	10/15/2014 9:34:14 AM
Method	MKE_tune_low_positive_20130204.m	Operator	Administrator
Sample Name	C-1321-1-LF+TW-M:E-3F5	Instrument	micrOTOF 72
	C-1321-1-LF+TW-M:E-3F5		

Acquisition Parameter

Source Type	ESI	Ion Polarity	Positive	Set Corrector Fill	79 V
Scan Range	n/a	Capillary Exit	120.0 V	Set Pulsar Pull	406 V
Scan Begin	50 m/z	Hexapole RF	90.0 V	Set Pulsar Push	388 V
Scan End	3000 m/z	Skimmer 1	45.5 V	Set Reflector	1300 V
		Hexapole 1	25.0 V	Set Flight Tube	9000 V
				Set Detector TOF	1910 V

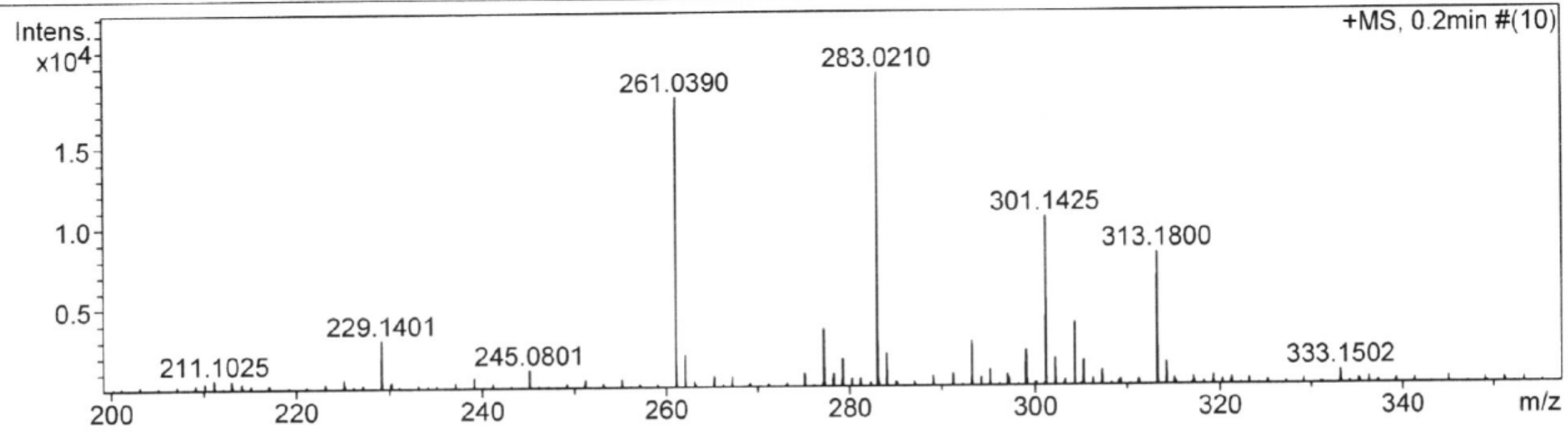

Figure S40. 500 MHz ^{1}H NMR spectrum of compound **8** in acetone-d_6

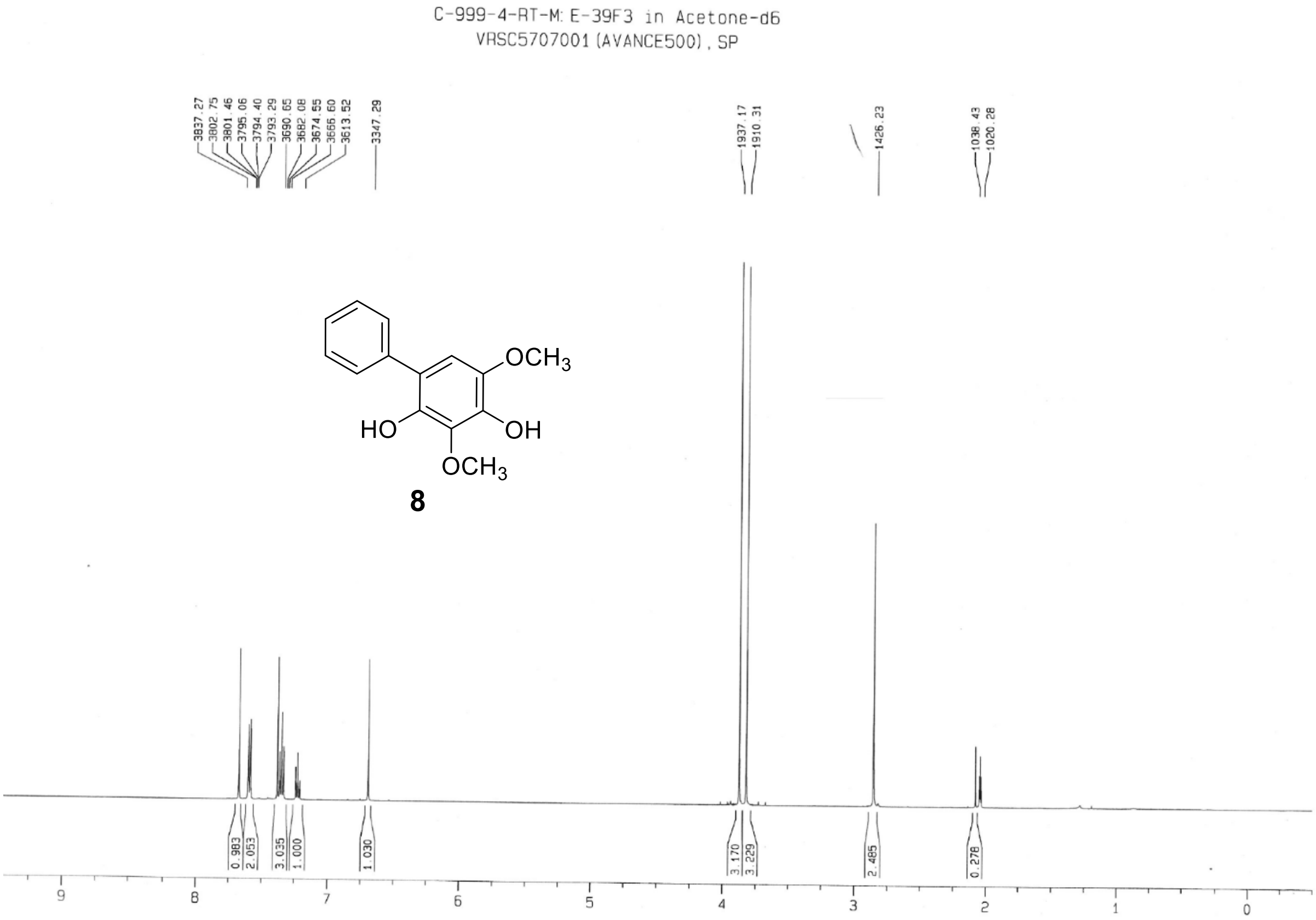

Figure S41. 125 MHz ^{13}C NMR spectrum of compound **8** in acetone-d_6

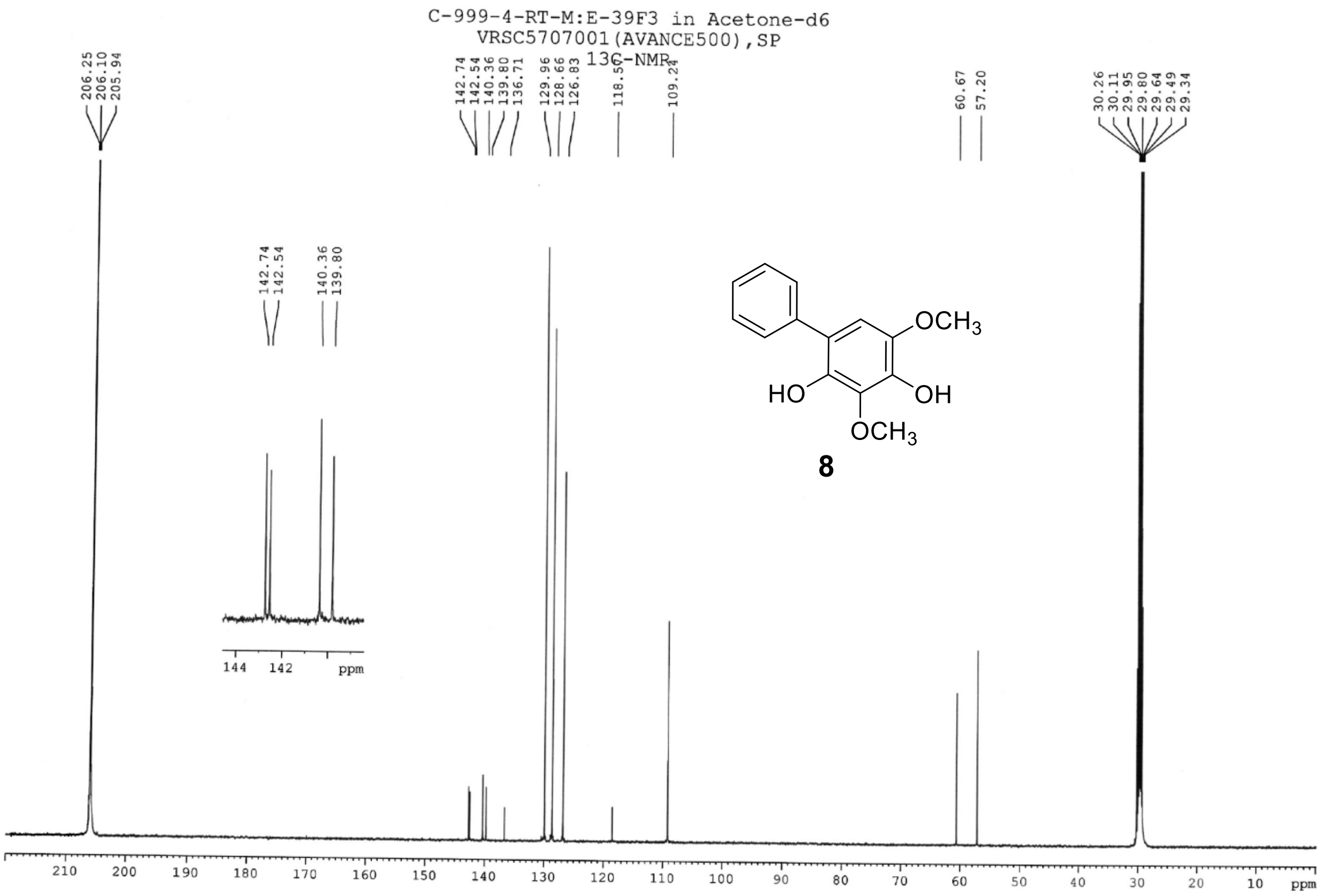

Figure S42. HRMS spectrum of compound **8**

Mass Spectrum List Report

Analysis Info

Analysis Name	VRSC570727002.d	Acquisition Date	7/27/2014 3:20:16 PM
Method	MKE_tune_low_positive_20130204.m	Operator	Administrator
Sample Name	C-999-4-RT-M:E-39F3	Instrument	micrOTOF 72
	C-999-4-RT-M:E-39F3		

Acquisition Parameter

Source Type	ESI	Ion Polarity	Positive	Set Corrector Fill	79 V
Scan Range	n/a	Capillary Exit	150.0 V	Set Pulsar Pull	406 V
Scan Begin	50 m/z	Hexapole RF	90.0 V	Set Pulsar Push	388 V
Scan End	3000 m/z	Skimmer 1	45.5 V	Set Reflector	1300 V
		Hexapole 1	25.0 V	Set Flight Tube	9000 V
				Set Detector TOF	1910 V

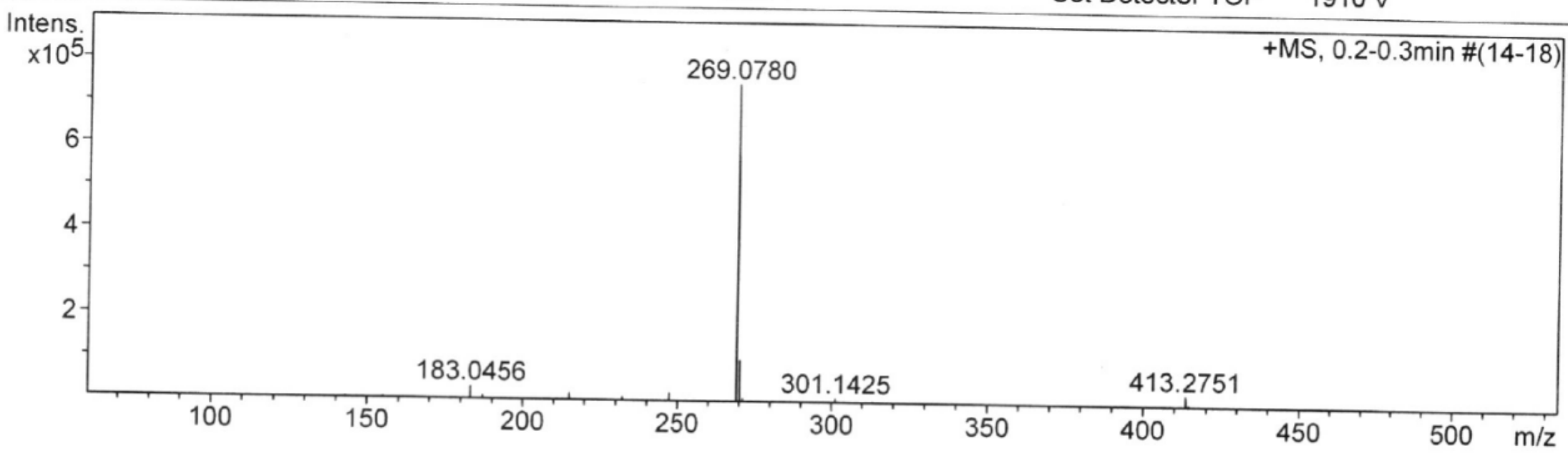

Figure S43. 500 MHz ^{1}H NMR spectrum of compound **9** in CD_3OD

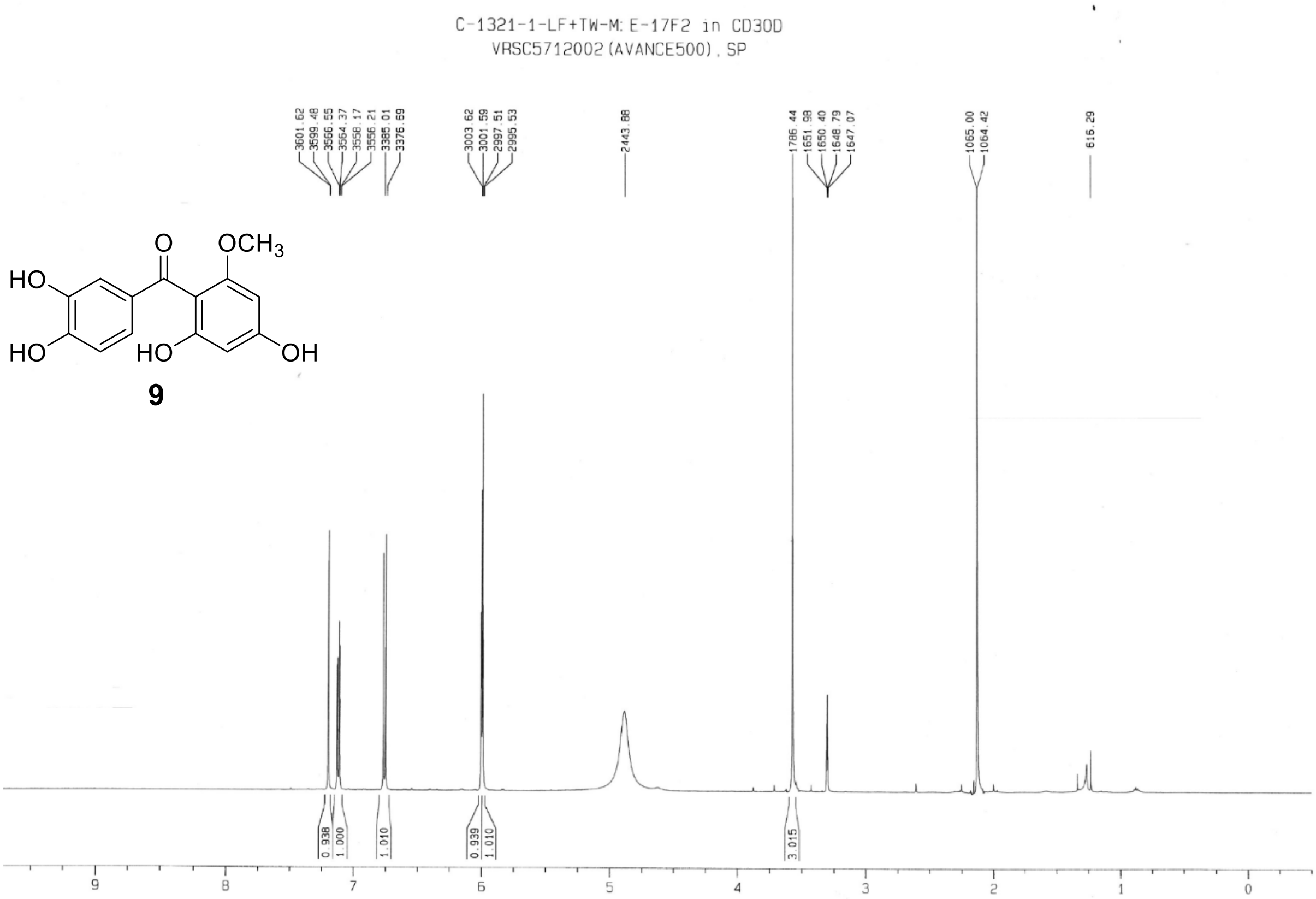

Figure S44. 125 MHz ^{13}C NMR spectrum of compound **9** in CD_3OD

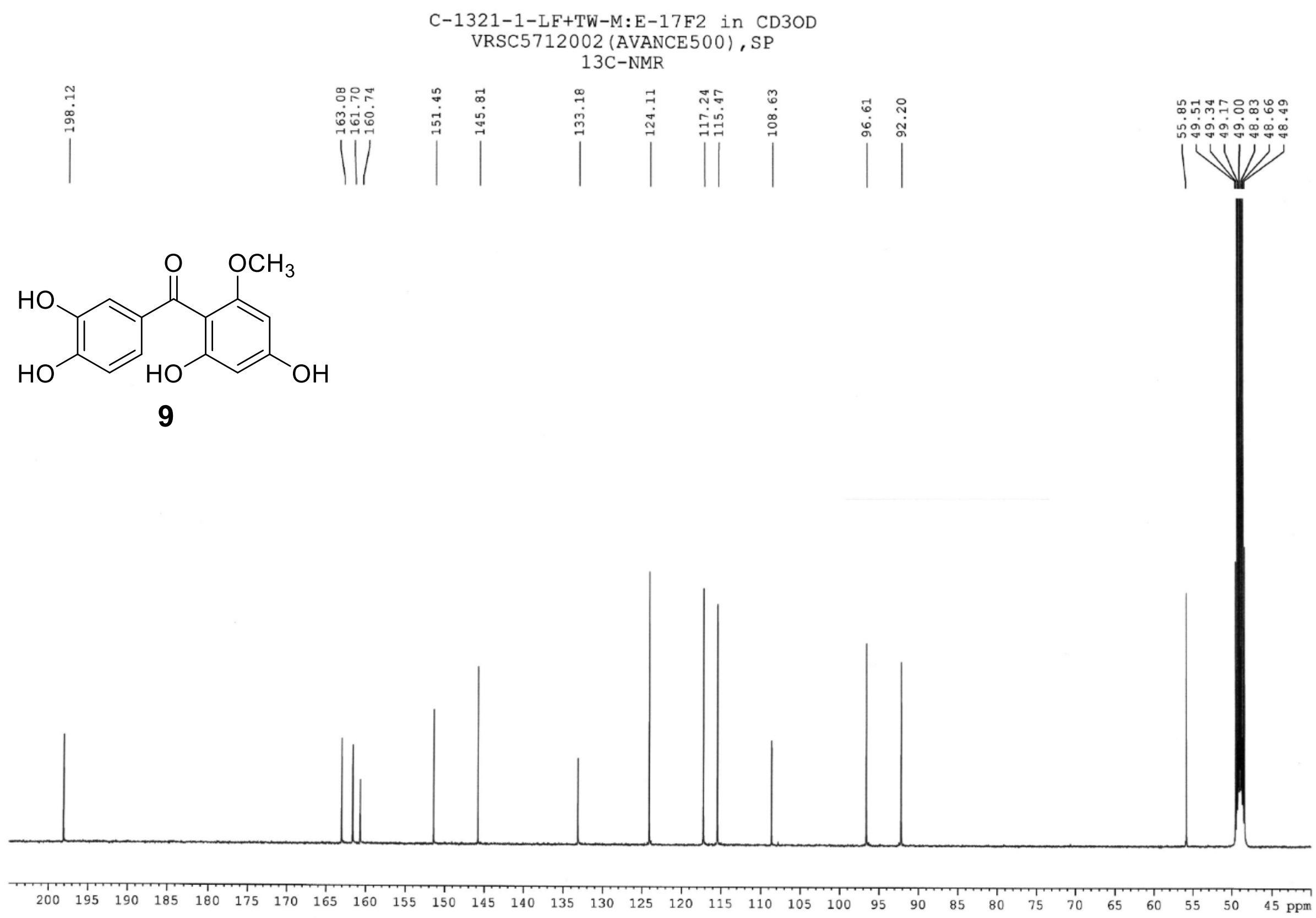

Figure S45. HRMS spectrum of compound **9**

Mass Spectrum List Report

Analysis Info

Analysis Name	VRSC571015004.d	Acquisition Date	10/15/2014 9:25:28 AM
Method	Tune_wide_POS_Natee20130403.m	Operator	Administrator
Sample Name	C-1321-1-LF+TW-M:E-3F4	Instrument	micrOTOF 72
	C-1321-1-LF+TW-M:E-3F4		

Acquisition Parameter

Source Type	ESI	Ion Polarity	Positive	Set Corrector Fill	79 V
Scan Range	n/a	Capillary Exit	120.0 V	Set Pulsar Pull	406 V
Scan Begin	50 m/z	Hexapole RF	200.0 V	Set Pulsar Push	388 V
Scan End	3000 m/z	Skimmer 1	45.0 V	Set Reflector	1300 V
		Hexapole 1	24.3 V	Set Flight Tube	9000 V
				Set Detector TOF	1910 V

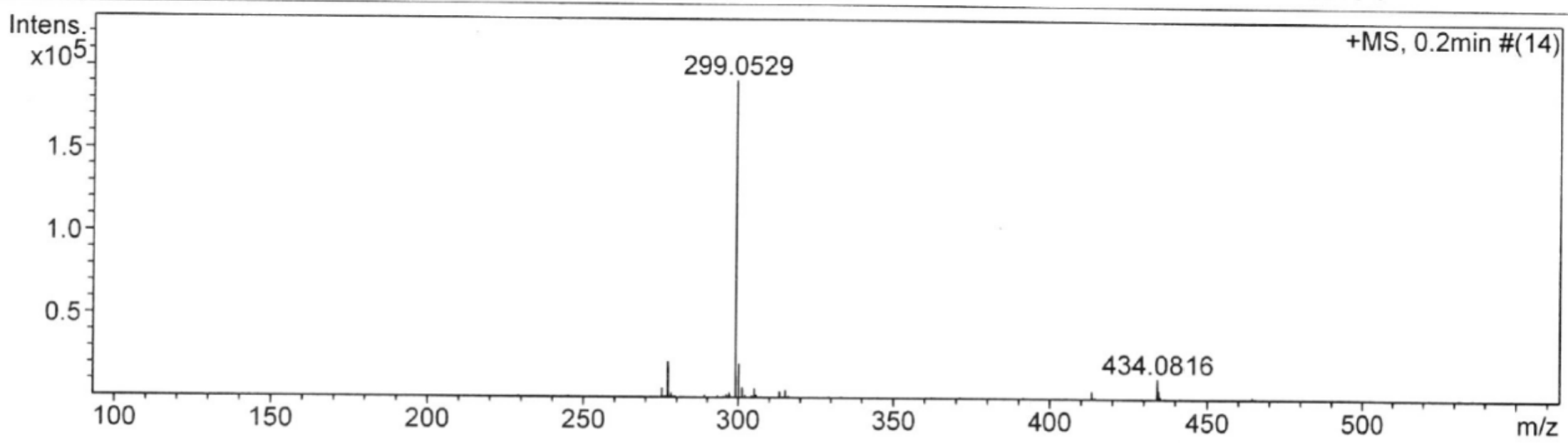

Figure S46. 500 MHz ^{1}H NMR spectrum of compound **10** in acetone-d_6

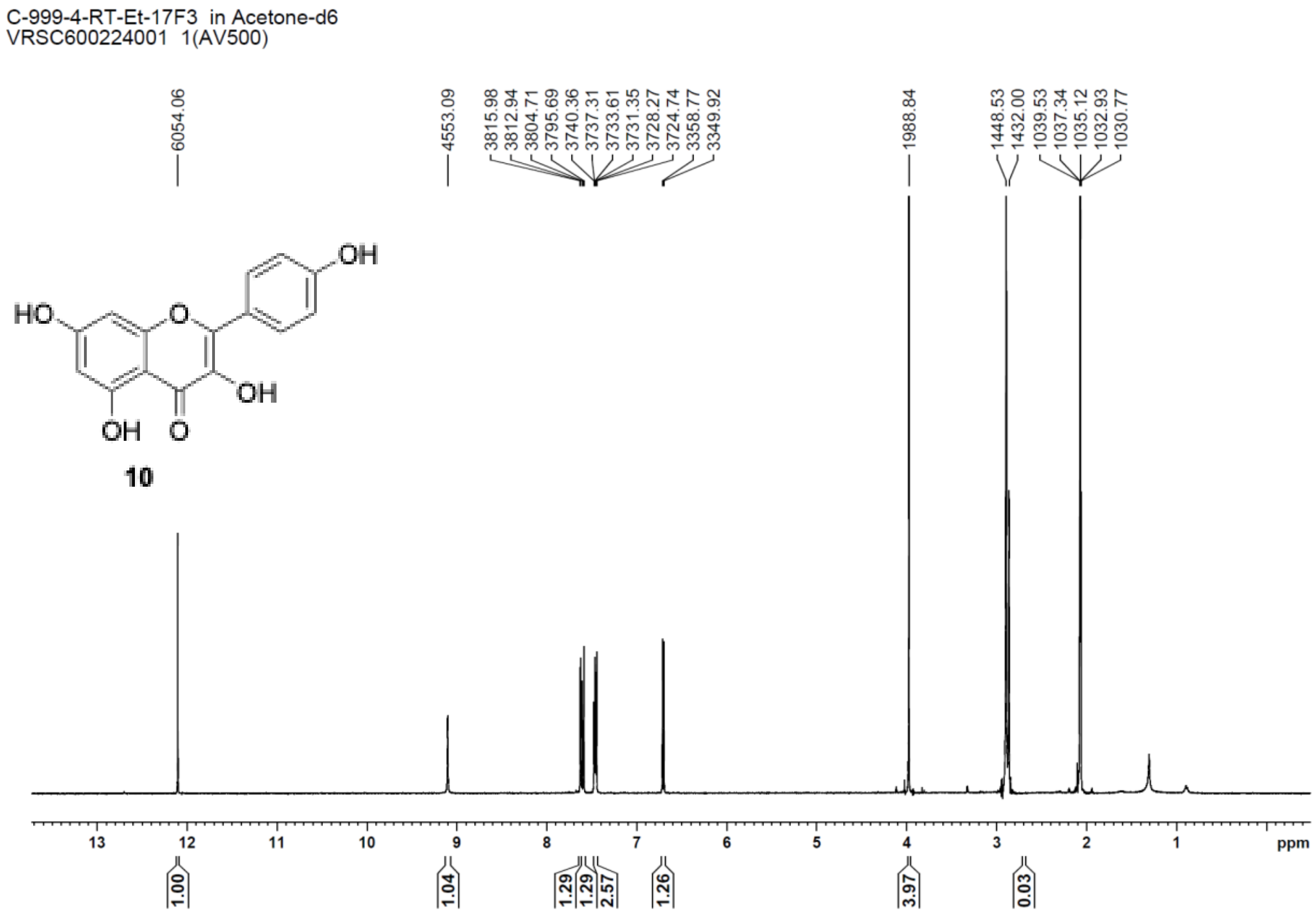

Figure S47. 125 MHz ^{13}C NMR spectrum of compound **10** in acetone-d_6

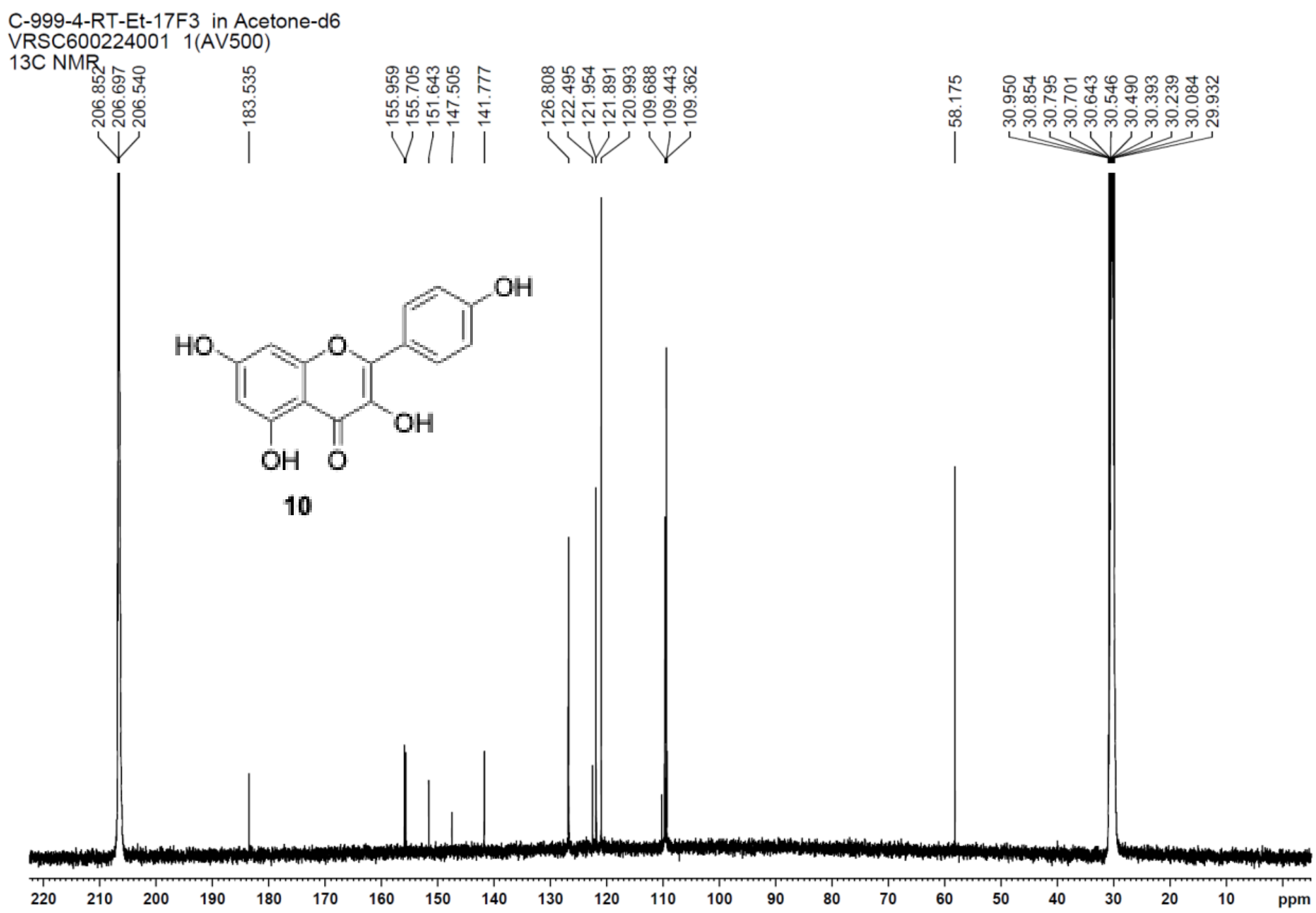

Figure S48. HRMS spectrum of compound **10**

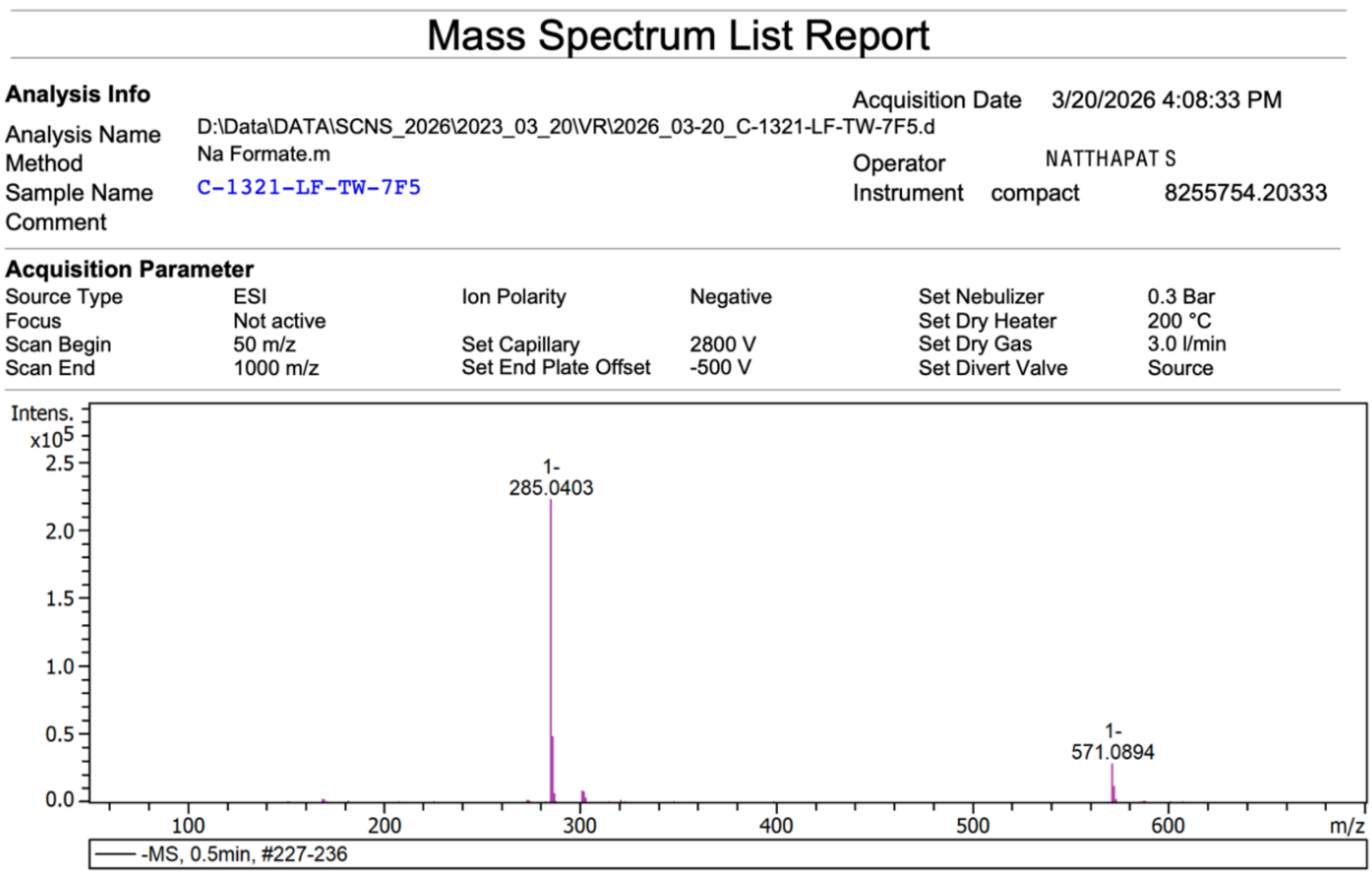

Figure S49. 500 MHz ^{1}H NMR spectrum of compound **11** in DMSO-d_6

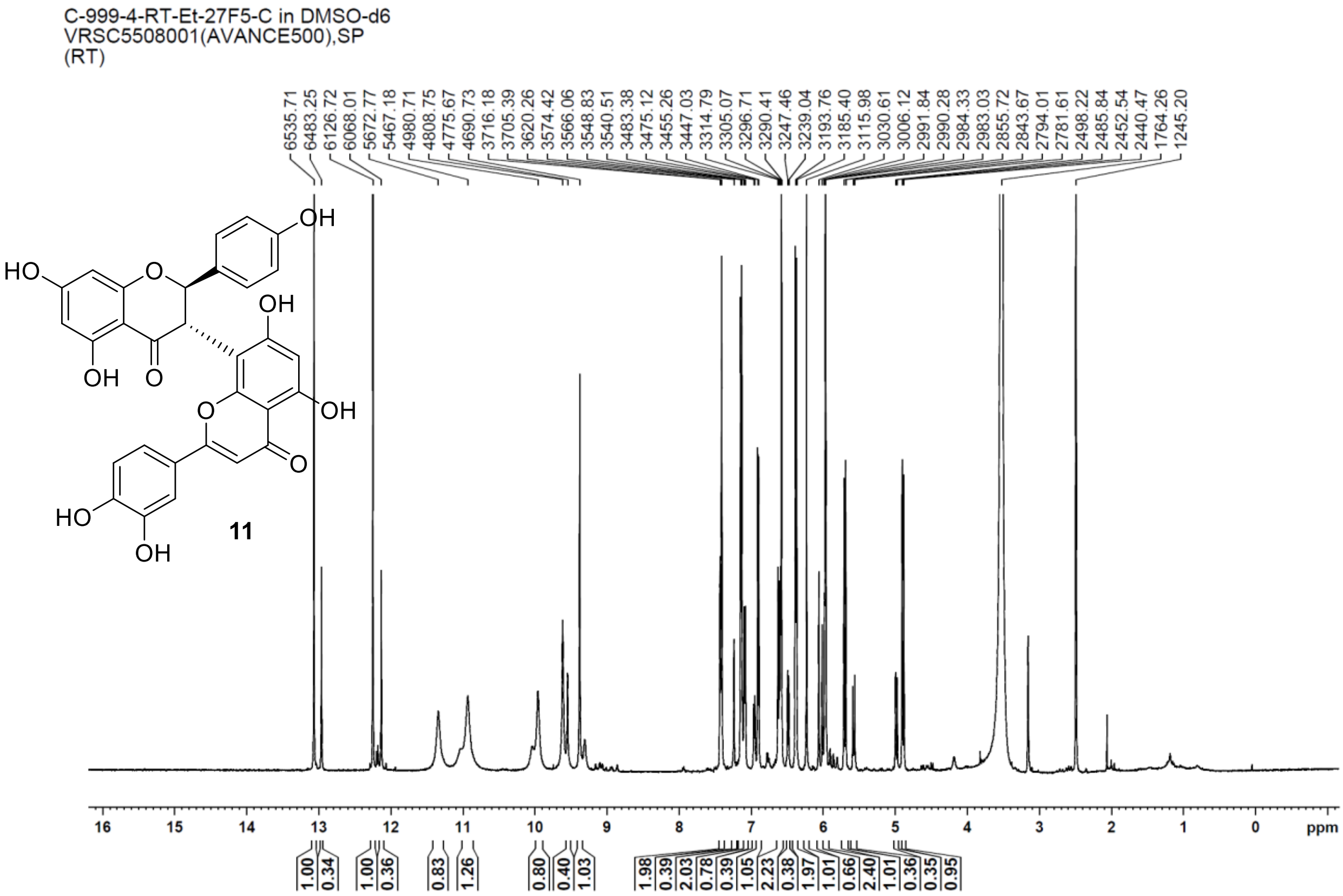

Figure S50. 125 MHz ^{13}C NMR spectrum of compound **11** in DMSO-d_6

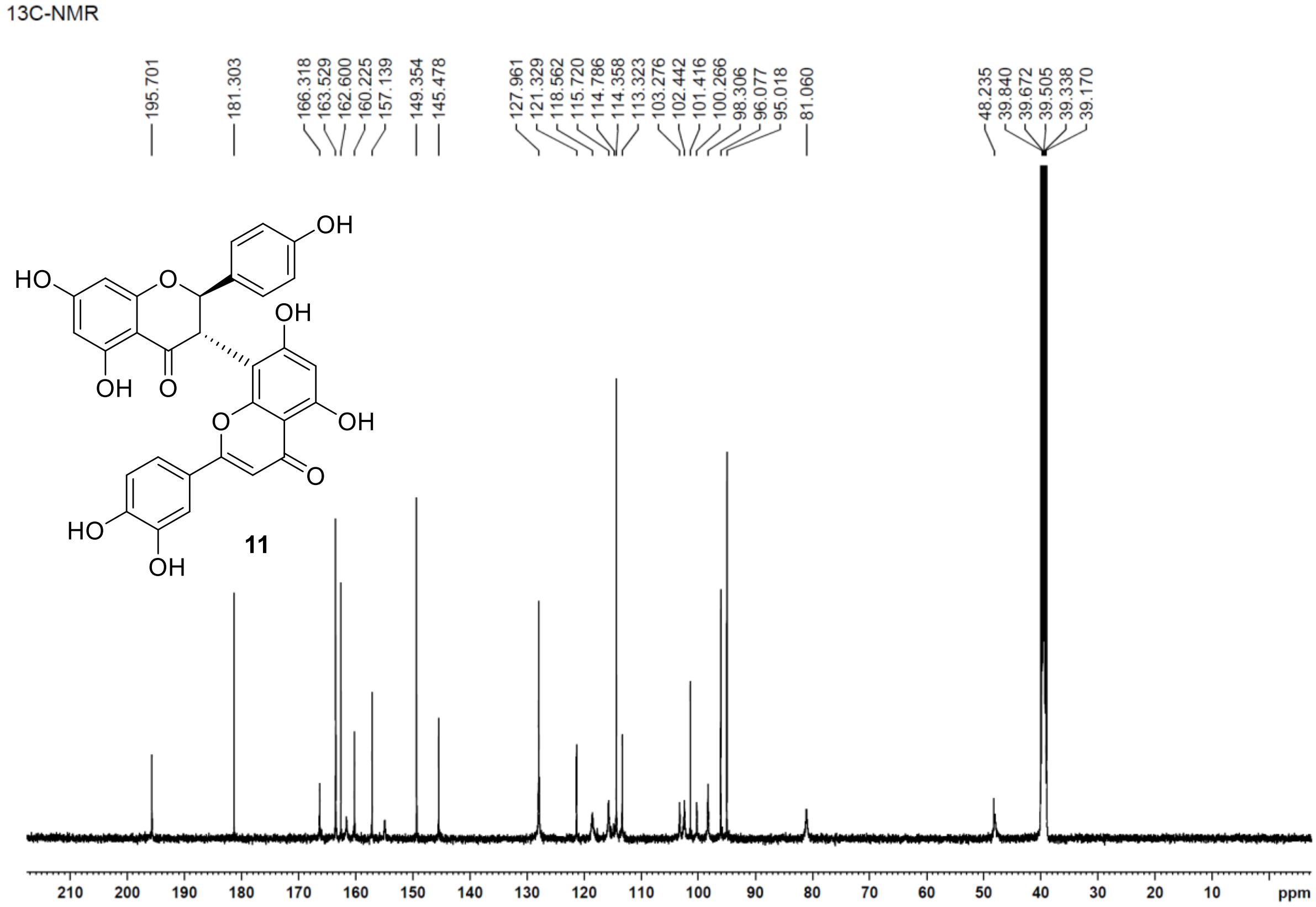

Figure S51. HRMS spectrum of compound **11**

Mass Spectrum List Report

Analysis Info

Analysis Name	VRSC20171229001.d	Acquisition Date	12/29/2017 2:48:35 PM
Method	Tune_wide_POS_Tawatchai_05Feb2016.m	Operator	Administrator
Sample Name	C-999-4-RT-Et-27F5-C 29-12-2017	Instrument	micrOTOF 72

Acquisition Parameter

Source Type	ESI	Ion Polarity	Positive	Set Corrector Fill	50 V
Scan Range	n/a	Capillary Exit	150.0 V	Set Pulsar Pull	337 V
Scan Begin	50 m/z	Hexapole RF	400.0 V	Set Pulsar Push	337 V
Scan End	3000 m/z	Skimmer 1	70.0 V	Set Reflector	1300 V
		Hexapole 1	25.0 V	Set Flight Tube	9000 V
				Set Detector TOF	2295 V

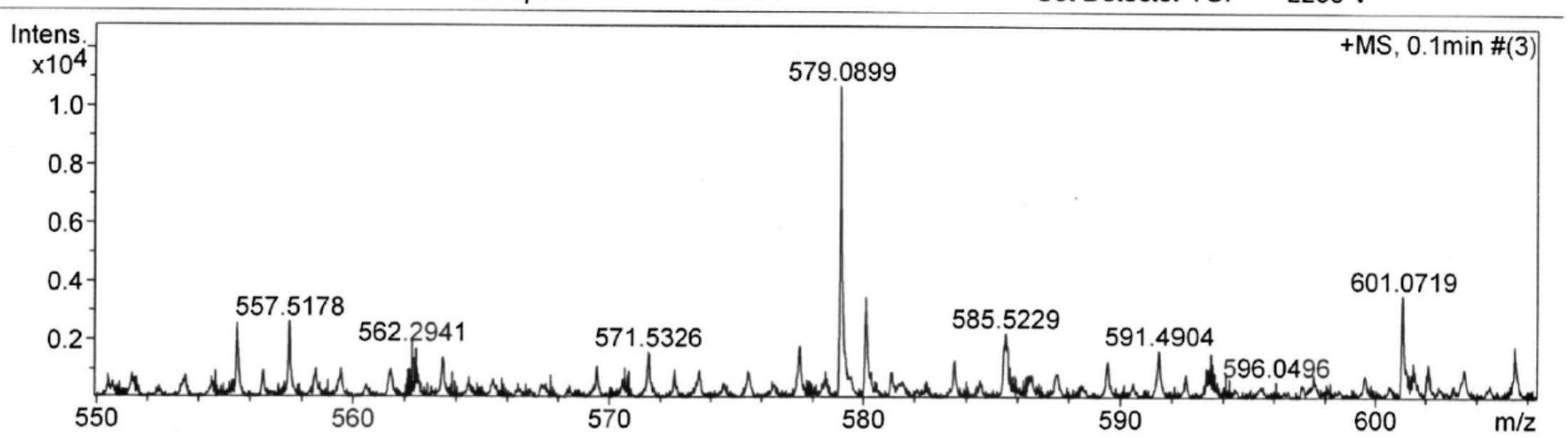

Figure S52. 500 MHz ^{1}H NMR spectrum of compound **12** in DMSO-d_6

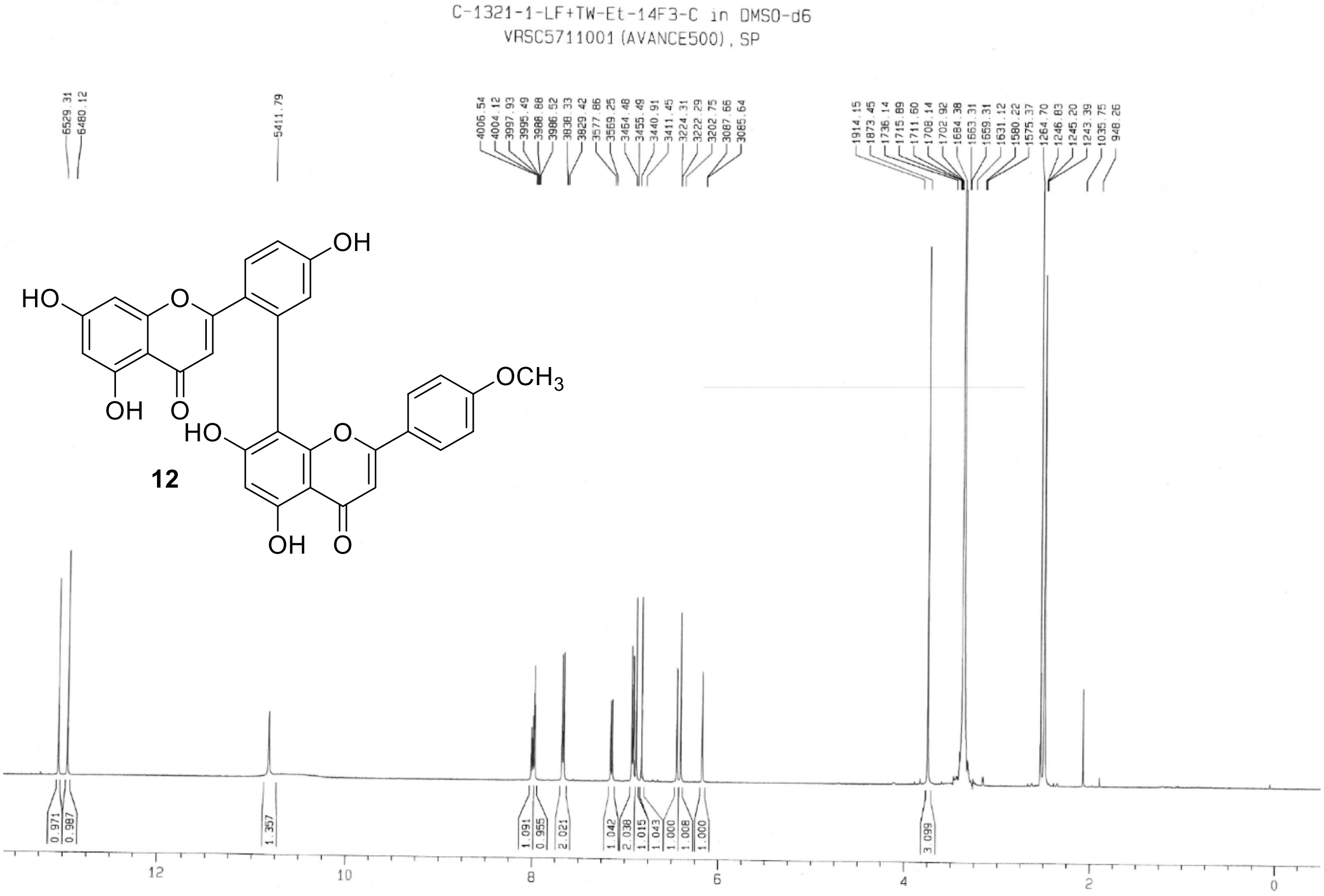

Figure S53. 125 MHz ^{13}C NMR spectrum of compound **12** in DMSO-d_6

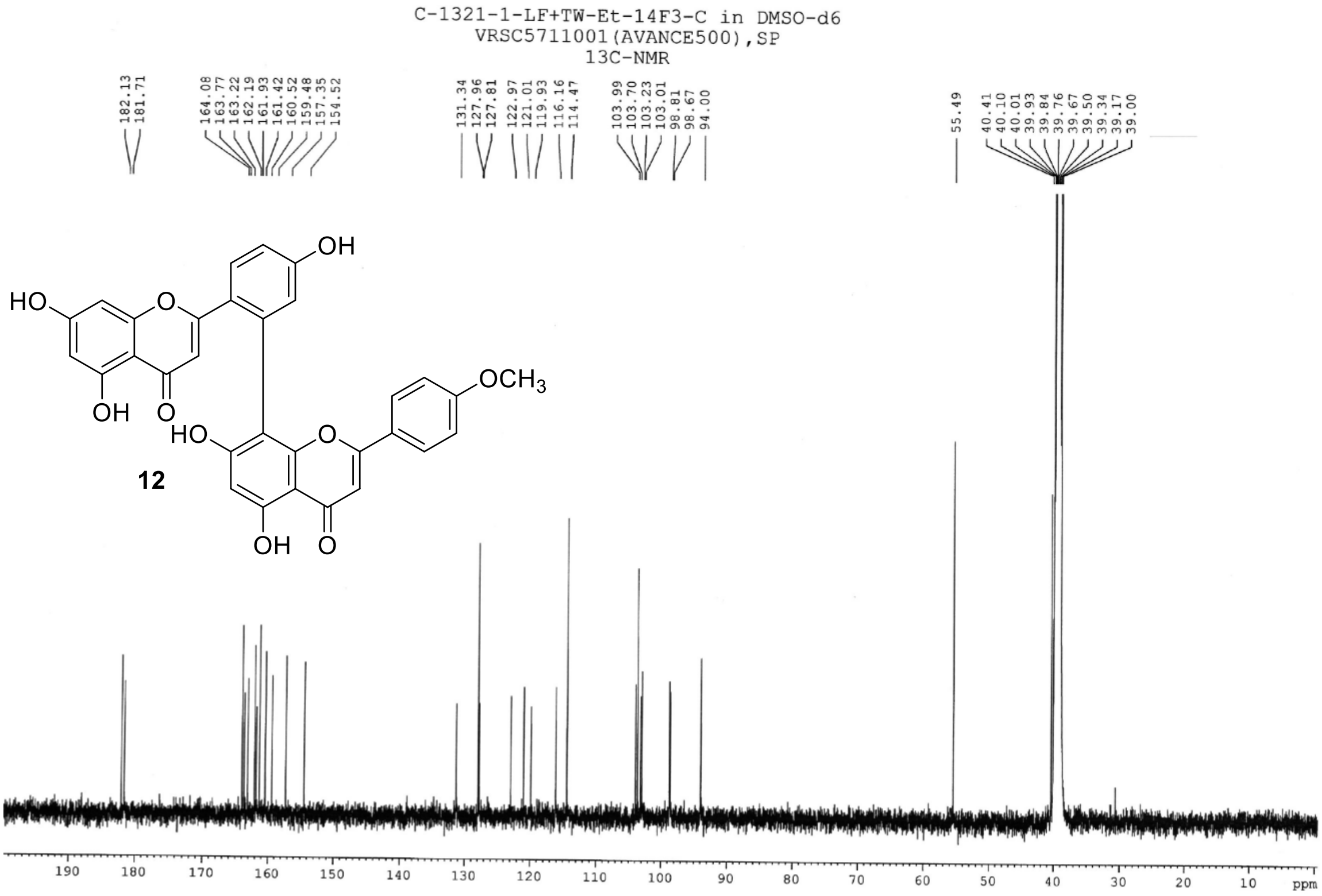

Figure S54. HRMS spectrum of compound **12**

Mass Spectrum List Report

Analysis Info

Analysis Name	VRSC570721003.d	Acquisition Date	7/21/2014 2:48:57 PM
Method	MKE_tune_wide_20130204.m	Operator	Administrator
Sample Name	C-1321-1-LF+TW-Et-14F3-C	Instrument	micrOTOF 72
	C-1321-1-LF+TW-Et-14F3-C		

Acquisition Parameter

				Set Corrector Fill	79 V
Source Type	ESI	Ion Polarity	Positive	Set Pulsar Pull	406 V
Scan Range	n/a	Capillary Exit	250.0 V	Set Pulsar Push	388 V
Scan Begin	50 m/z	Hexapole RF	400.0 V	Set Reflector	1300 V
Scan End	3000 m/z	Skimmer 1	45.0 V	Set Flight Tube	9000 V
		Hexapole 1	25.0 V	Set Detector TOF	1910 V

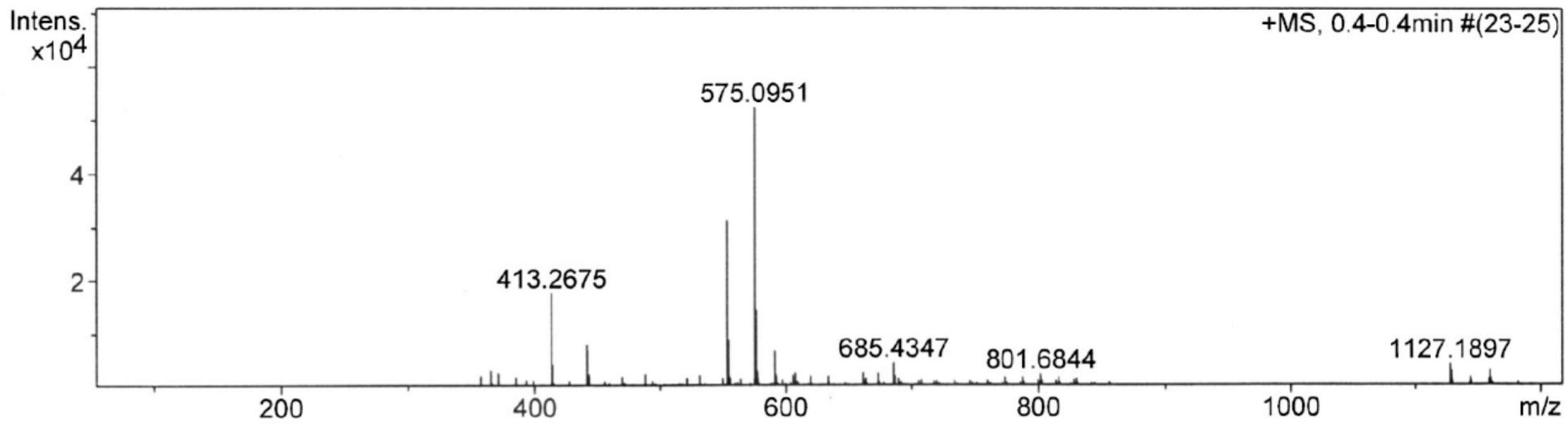

Figure S55. 500 MHz ^{1}H NMR spectrum of compound **13** in $CDCl_3$

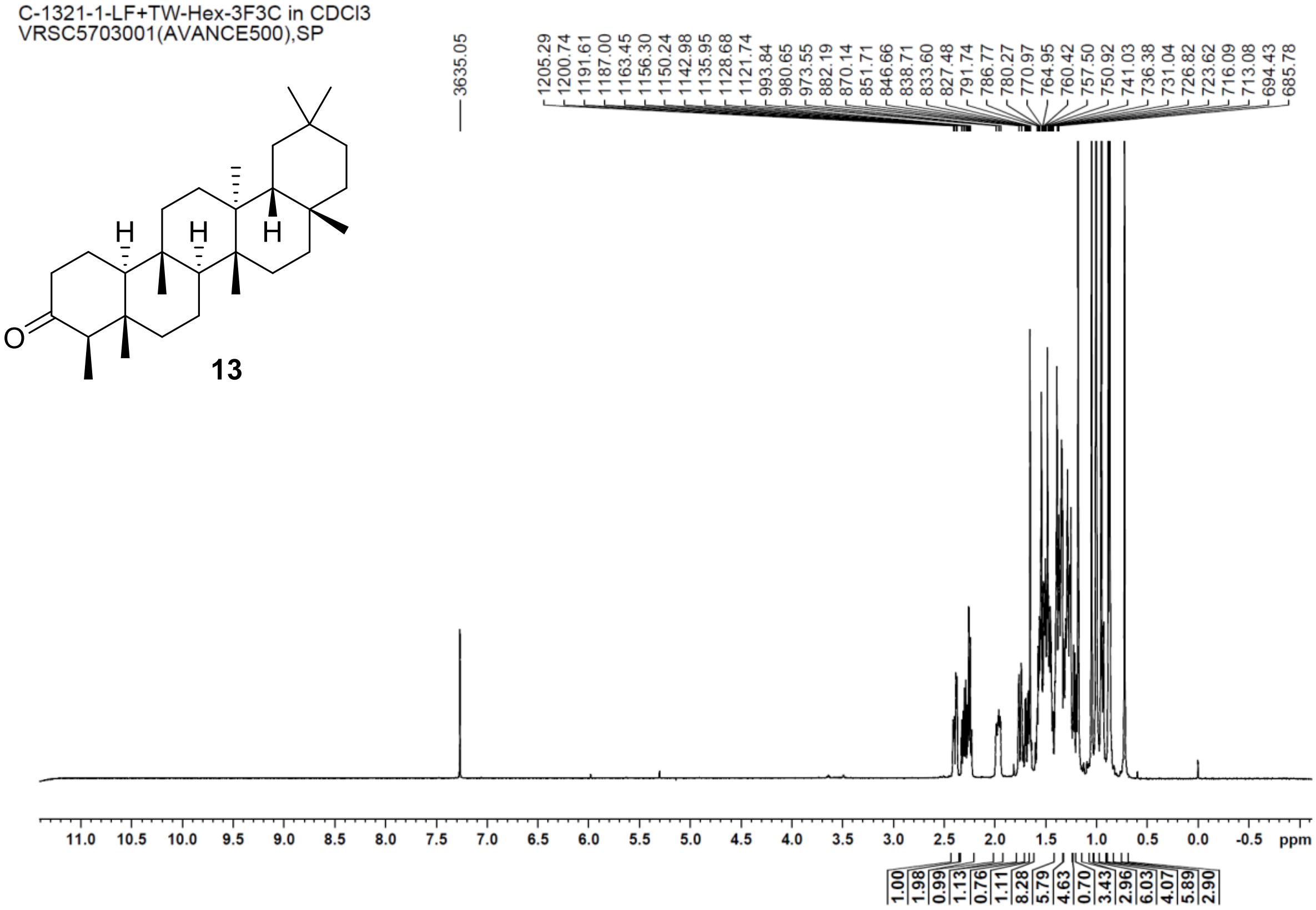

Figure S56. 125 MHz ^{13}C NMR spectrum of compound **13** in $CDCl_3$

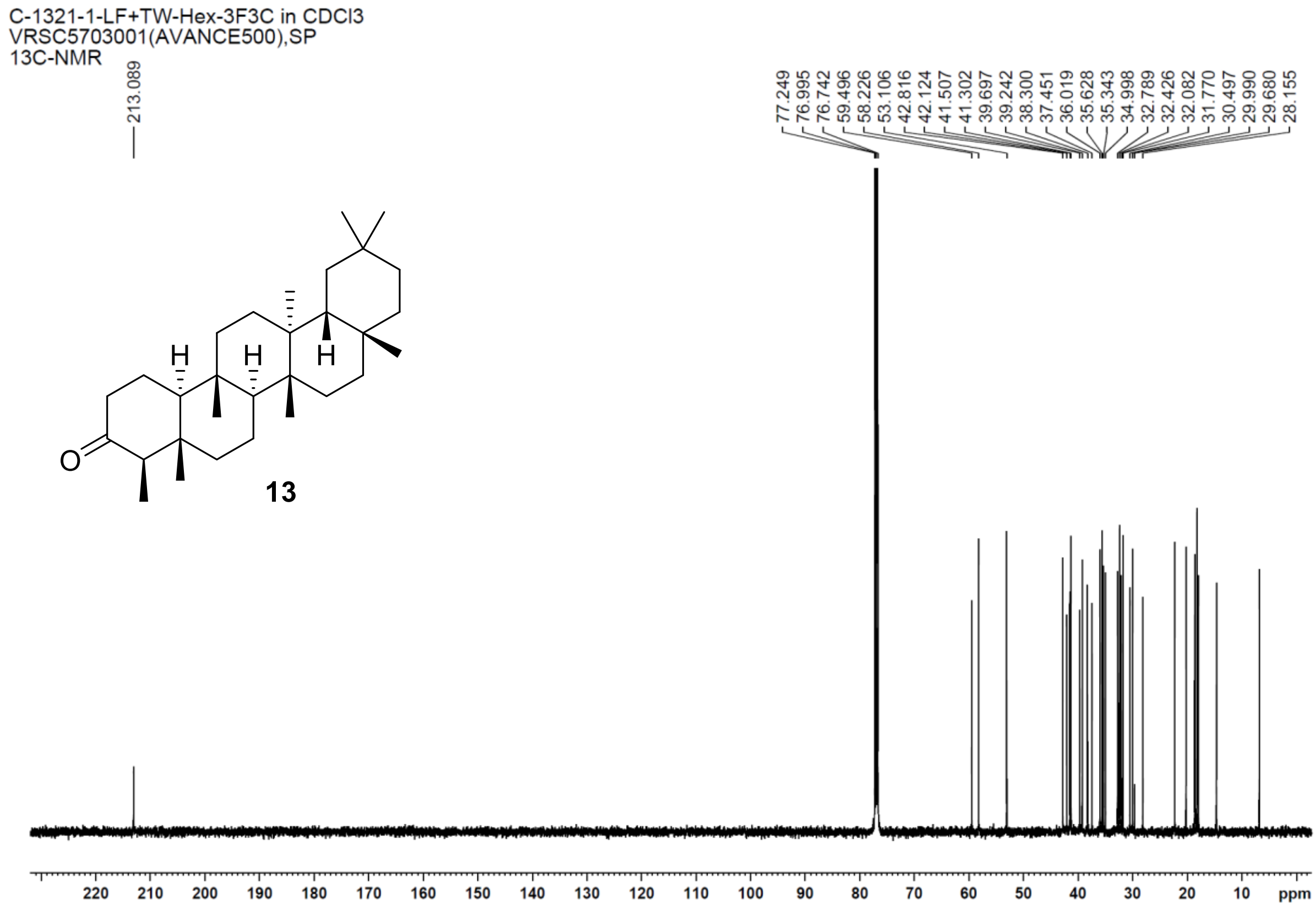

Figure S57. HRMS spectrum of compound **13**

Mass Spectrum List Report

Analysis Info

Analysis Name	VRSC570303001.d	Acquisition Date	3/3/2014 11:32:46 AM
Method	MKE_tune_low_positive_20130204.m	Operator	Administrator
Sample Name	C-1321-1-LF+TW-Hex-3F3-C	Instrument	micrOTOF 72
	C-1321-1-LF+TW-Hex-3F3-C		

Acquisition Parameter

Source Type	ESI	Ion Polarity	Positive	Set Corrector Fill	79 V
Scan Range	n/a	Capillary Exit	90.0 V	Set Pulsar Pull	406 V
Scan Begin	50 m/z	Hexapole RF	90.0 V	Set Pulsar Push	388 V
Scan End	3000 m/z	Skimmer 1	45.5 V	Set Reflector	1300 V
		Hexapole 1	25.0 V	Set Flight Tube	9000 V
				Set Detector TOF	1910 V

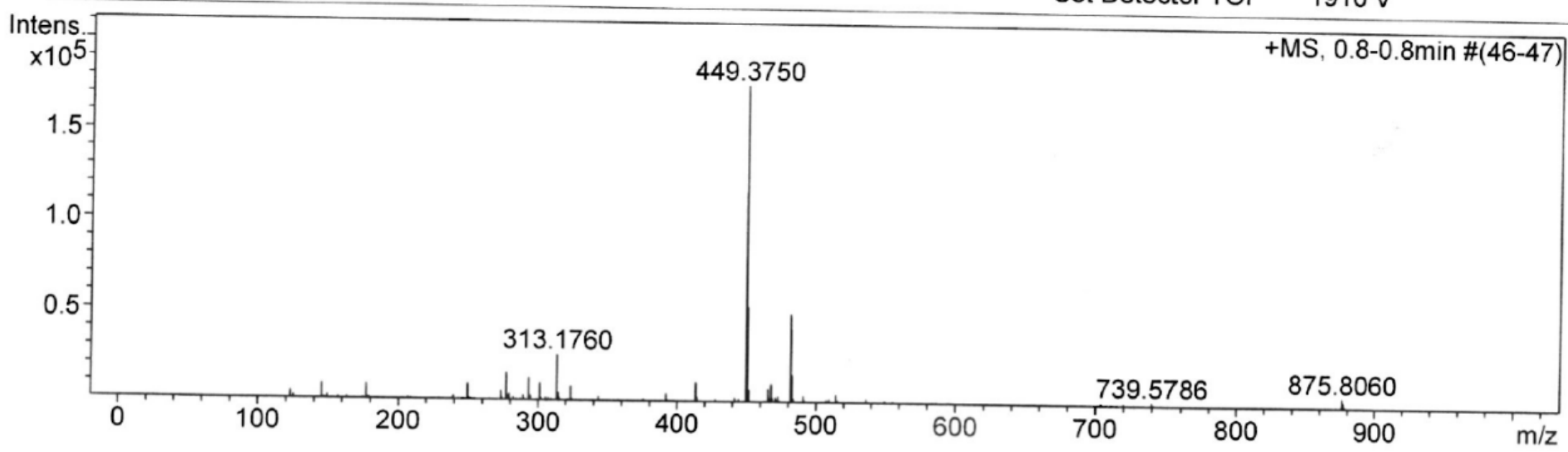

Figure S58. 500 MHz ^{1}H NMR spectrum of compound **14** in $CDCl_3$

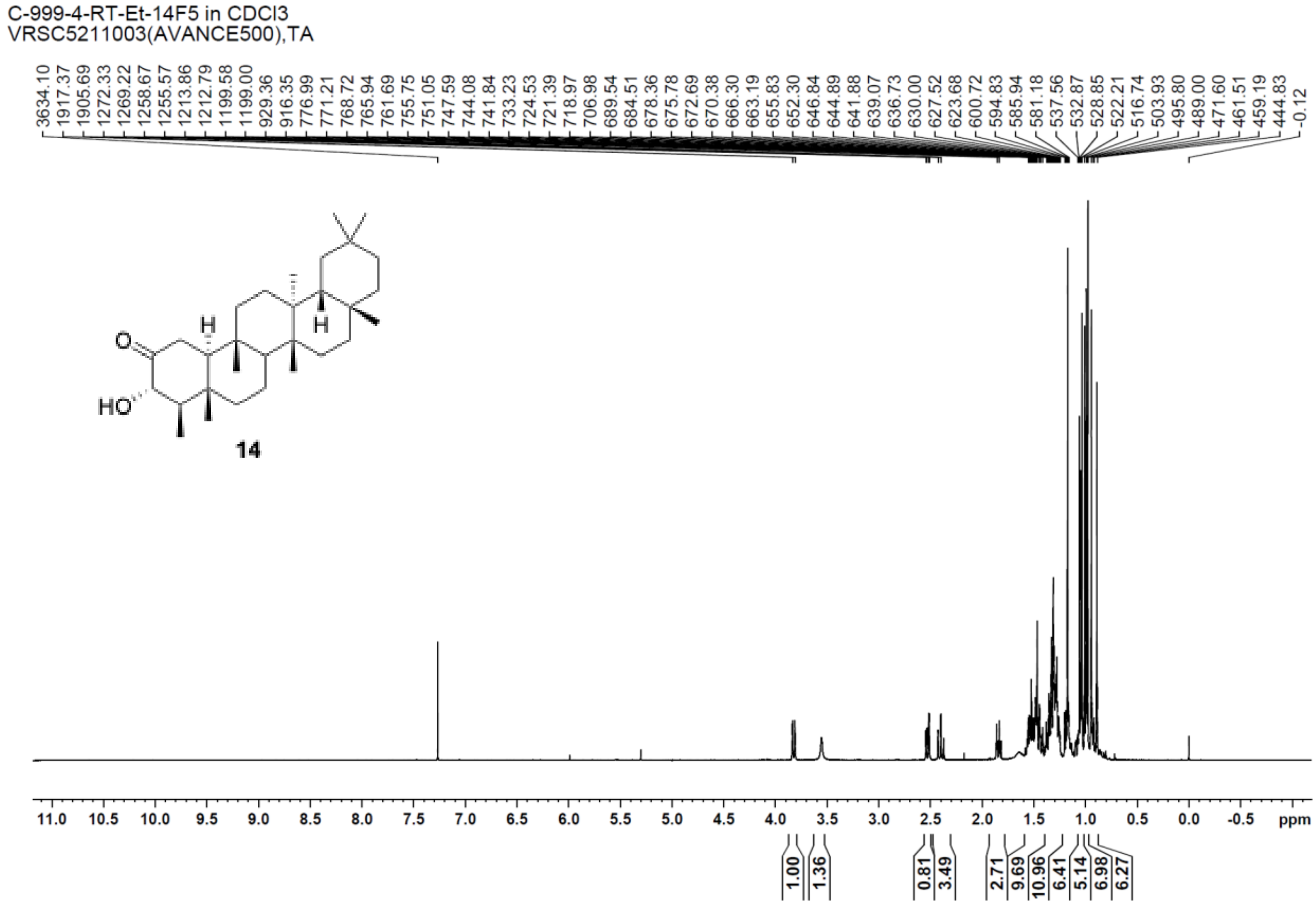

Figure S59. 125 MHz ^{13}C NMR spectrum of compound **14** in $CDCl_3$

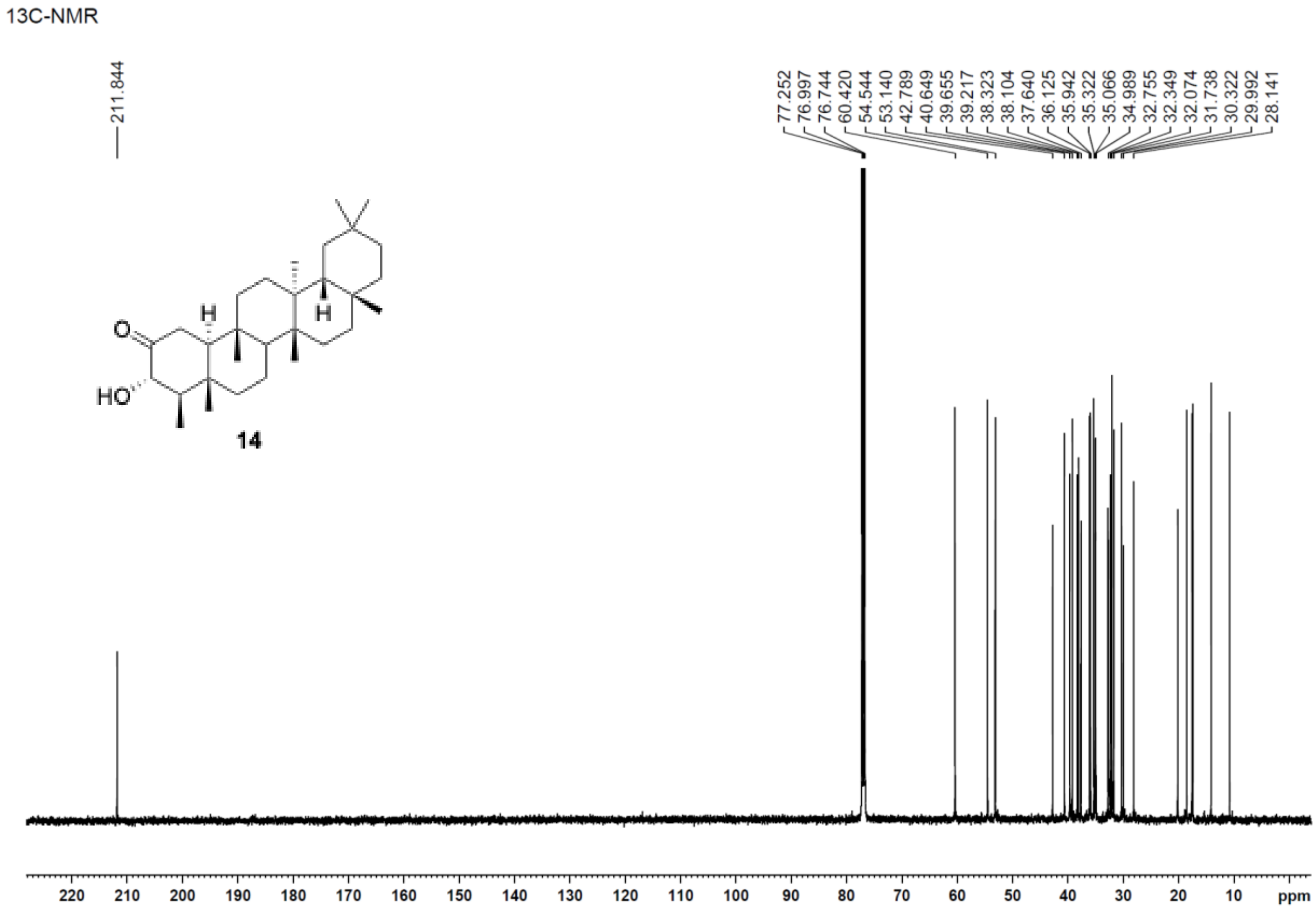

Figure S60. HRMS spectrum of compound **14**

Mass Spectrum List Report

Analysis Info

Analysis Name	VRSC0170808003.d	Acquisition Date	8/8/2017 9:08:55 PM
Method	Tune_wide_POS_Natee20130403.m	Operator	Administrator
Sample Name	C-999-4-RT-ET-14F5 CKJP20170808	Instrument	micrOTOF 72

Acquisition Parameter

Source Type	ESI	Ion Polarity	Positive	Set Corrector Fill	50 V
Scan Range	n/a	Capillary Exit	200.0 V	Set Pulsar Pull	337 V
Scan Begin	50 m/z	Hexapole RF	600.0 V	Set Pulsar Push	337 V
Scan End	3000 m/z	Skimmer 1	70.0 V	Set Reflector	1300 V
		Hexapole 1	25.0 V	Set Flight Tube	9000 V
				Set Detector TOF	2295 V

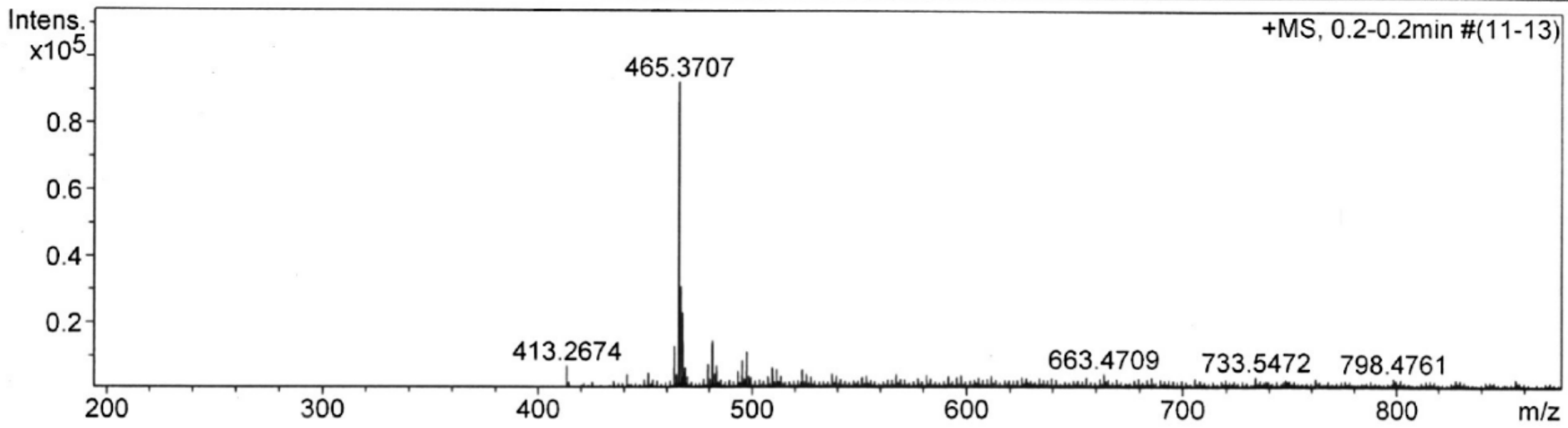

Figure S61. 500 MHz ^{1}H NMR spectrum of compound **15** in $CDCl_3$

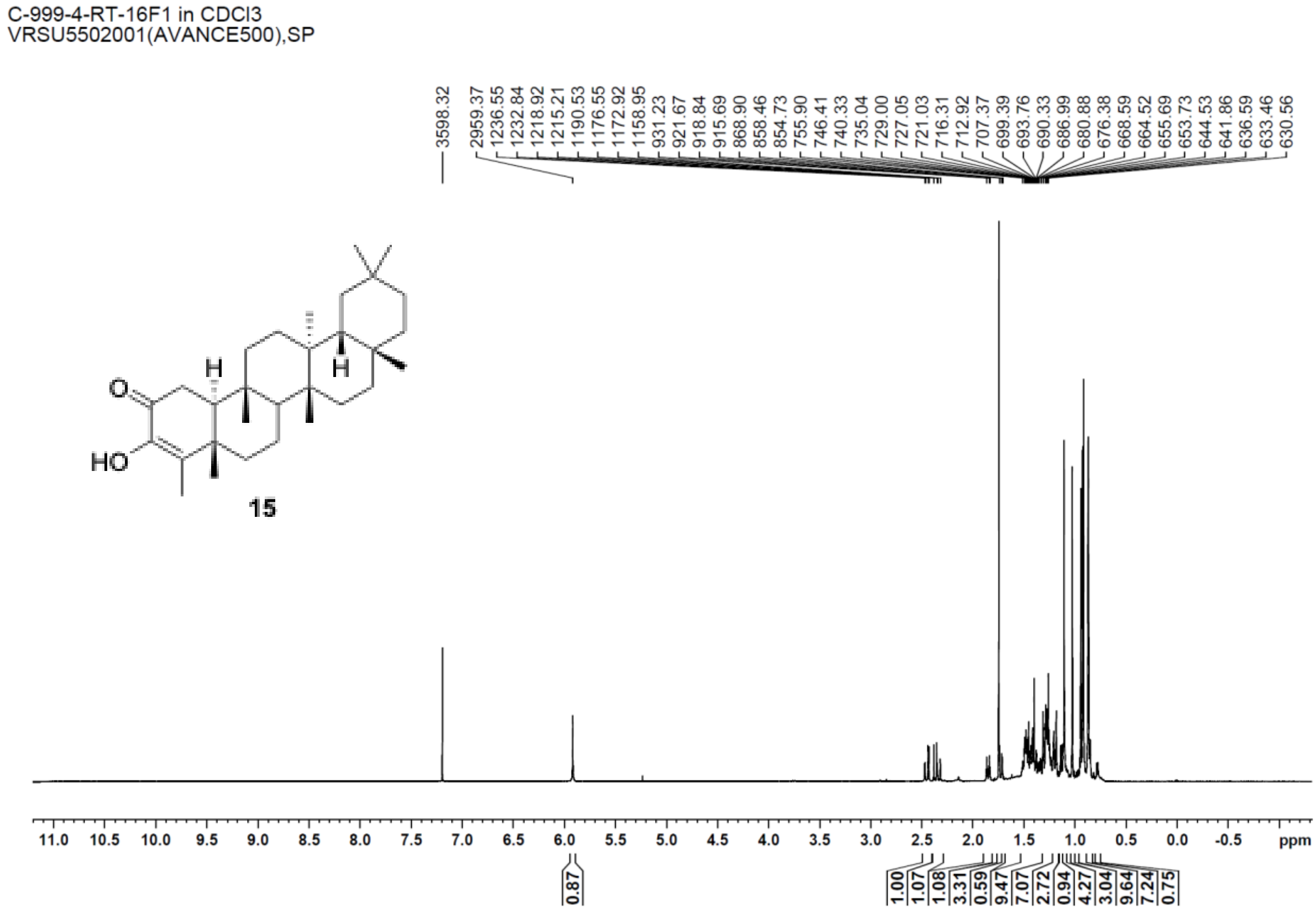

Figure S62. 125 MHz ^{13}C NMR spectrum of compound **15** in $CDCl_3$

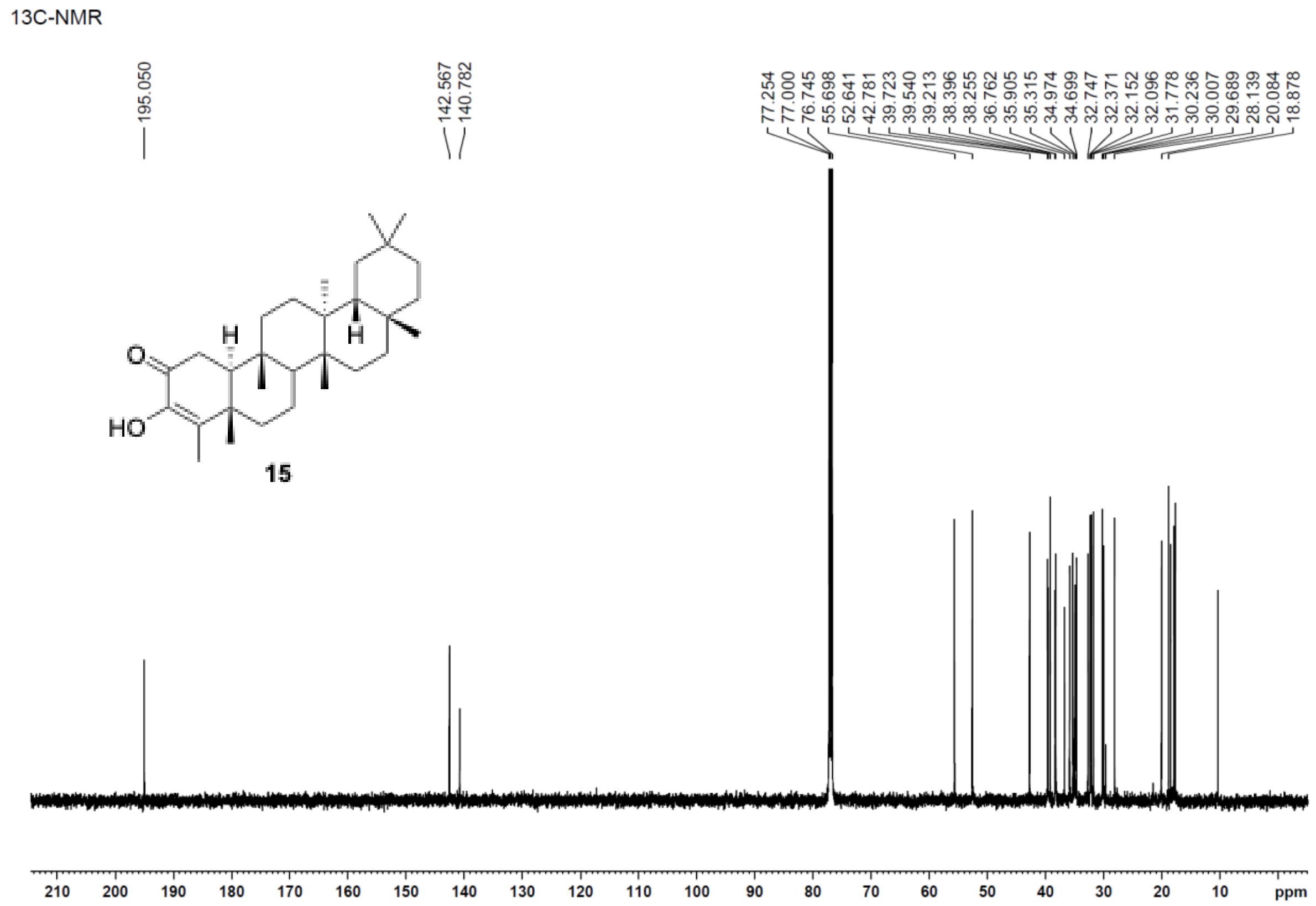

Figure S63. HRMS spectrum of compound **15**

Mass Spectrum List Report

Analysis Info

Analysis Name	VRSC0170808002.d	Acquisition Date	8/8/2017 9:07:15 PM
Method	Tune_wide_POS_Natee20130403.m	Operator	Administrator
Sample Name	C-999-4-RT-ET-15F1	Instrument	micrOTOF 72
	CKJP20170808		

Acquisition Parameter

Source Type	ESI	Ion Polarity	Positive	Set Corrector Fill	50 V
Scan Range	n/a	Capillary Exit	200.0 V	Set Pulsar Pull	337 V
Scan Begin	50 m/z	Hexapole RF	600.0 V	Set Pulsar Push	337 V
Scan End	3000 m/z	Skimmer 1	70.0 V	Set Reflector	1300 V
		Hexapole 1	25.0 V	Set Flight Tube	9000 V
				Set Detector TOF	2295 V

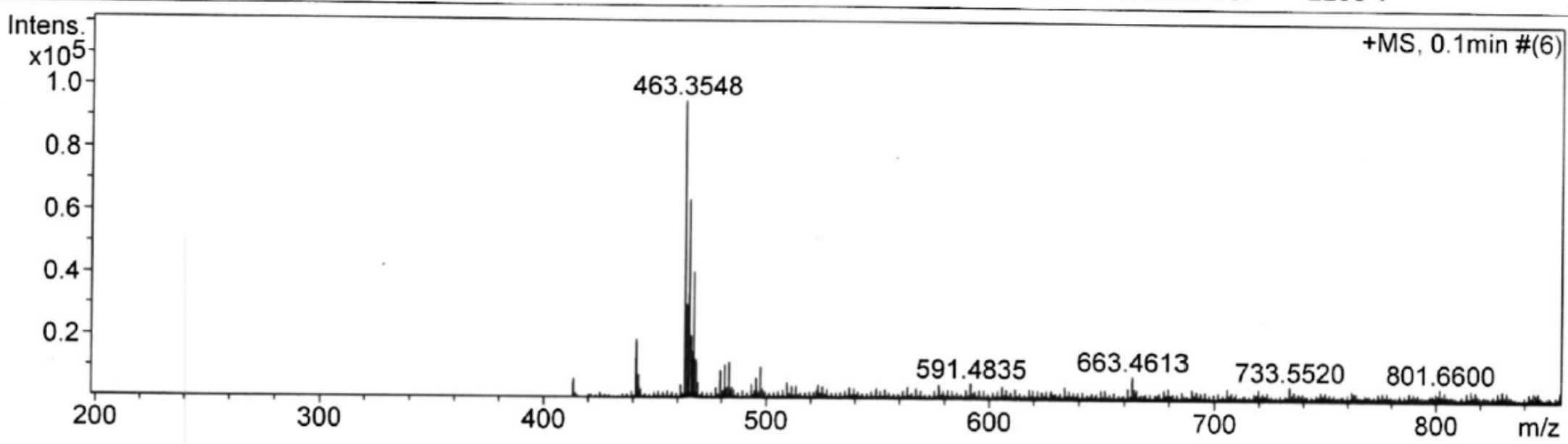

Figure S64. 500 MHz ^{1}H NMR spectrum of compound **16** in $CDCl_3$

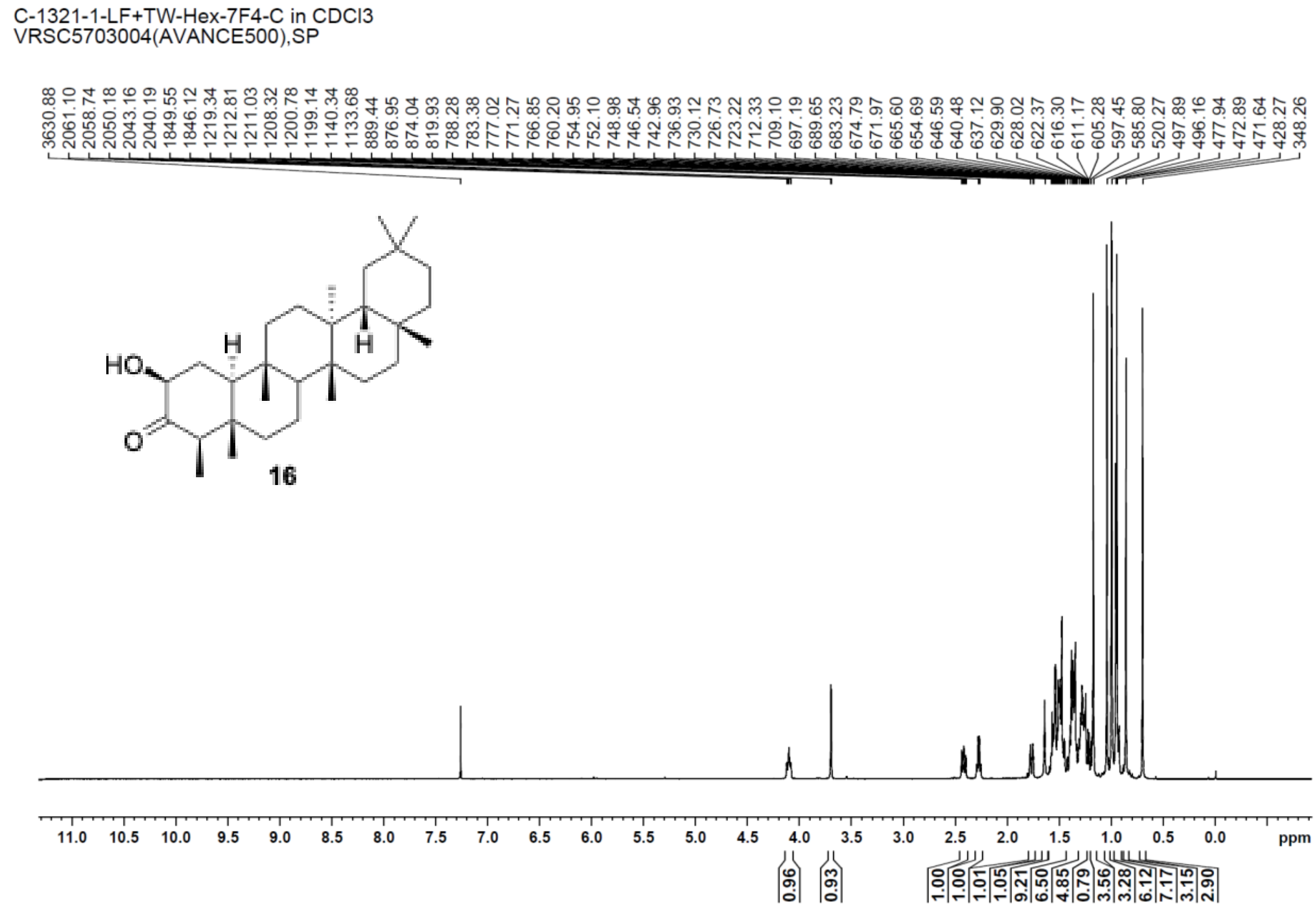

Figure S65. 125 MHz ^{13}C NMR spectrum of compound **16** in $CDCl_3$

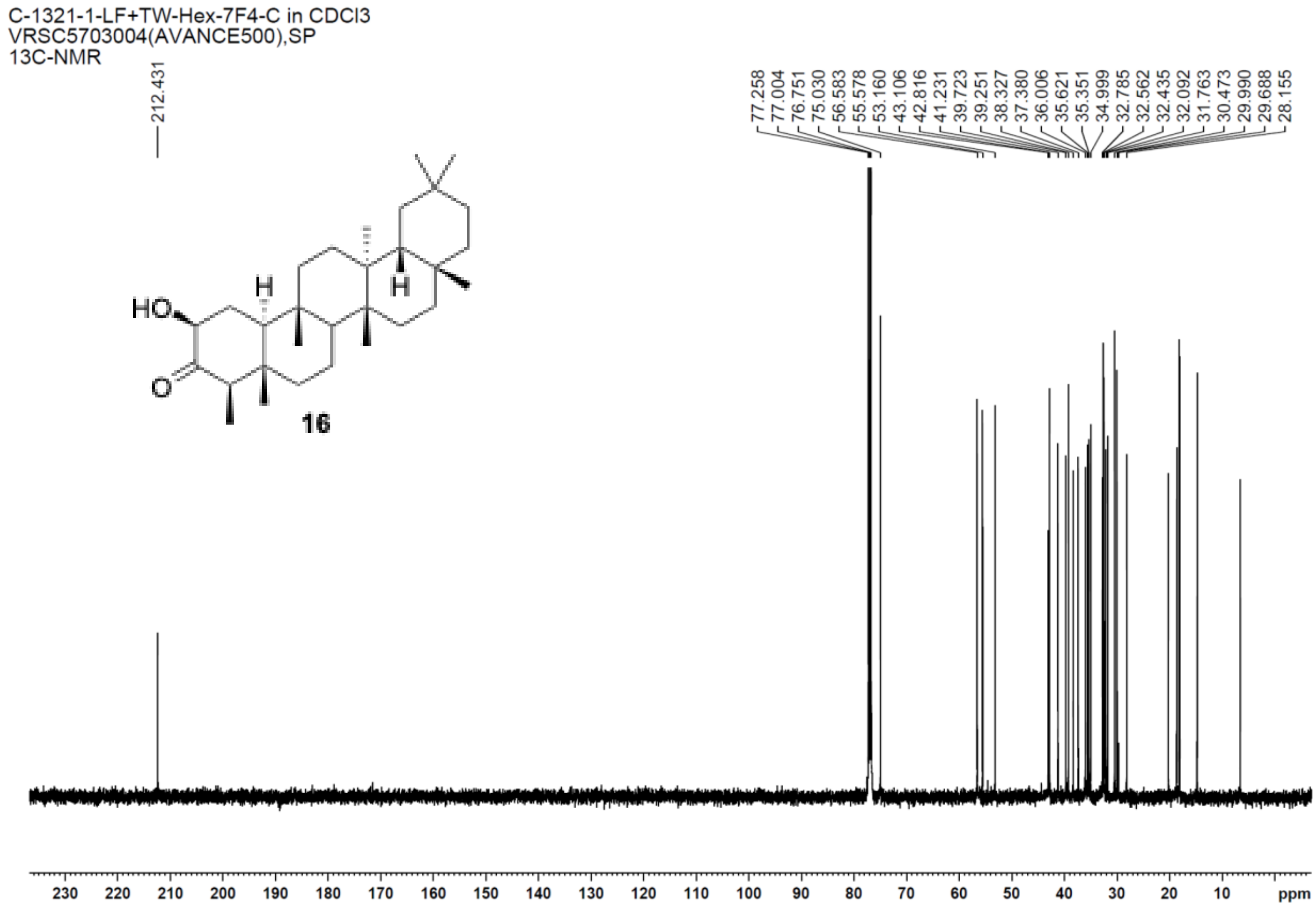

Figure S66. HRMS spectrum of compound **16**

Mass Spectrum List Report

Analysis Info

Analysis Name	VRSC570328001.d	Acquisition Date	3/28/2014 12:41:04 PM
Method	MKE_tune_wide_20130204.m	Operator	Administrator
Sample Name	C-1321-1-LF-TW-HEX-7F4-C	Instrument	micrOTOF 72
	C-1321-1-LF-TW-HEX-7F4-C		

Acquisition Parameter

Source Type	ESI	Ion Polarity	Positive	Set Corrector Fill	79 V
Scan Range	n/a	Capillary Exit	180.0 V	Set Pulsar Pull	406 V
Scan Begin	50 m/z	Hexapole RF	400.0 V	Set Pulsar Push	388 V
Scan End	3000 m/z	Skimmer 1	45.0 V	Set Reflector	1300 V
		Hexapole 1	25.0 V	Set Flight Tube	9000 V
				Set Detector TOF	1910 V

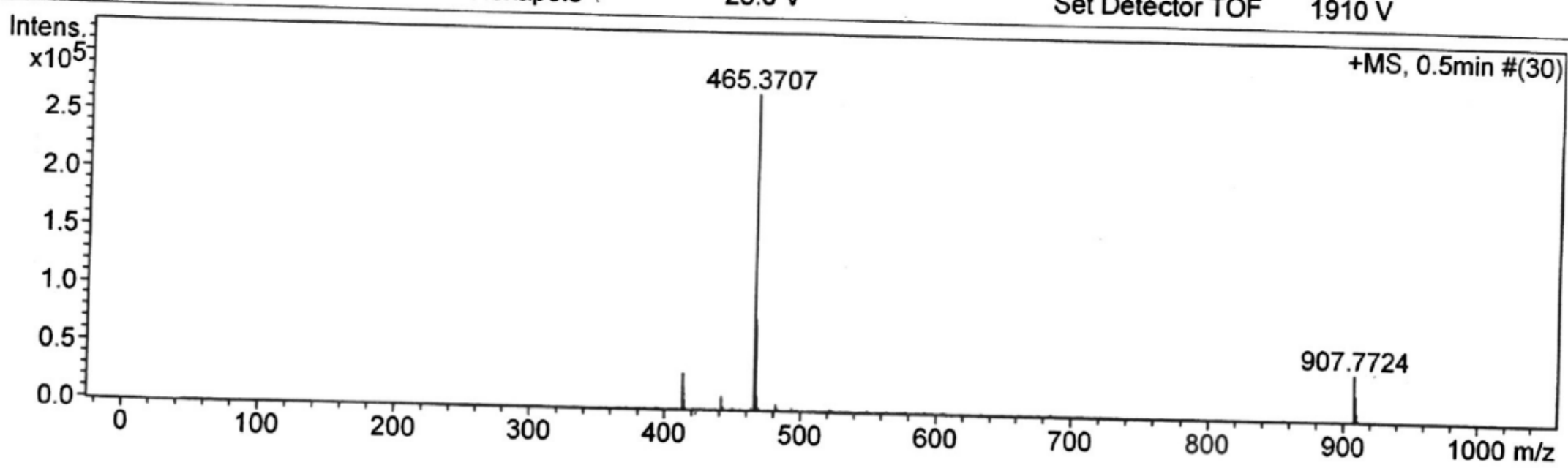

Figure S67. 500 MHz ^{1}H NMR spectrum of compound **17** in $CDCl_3$

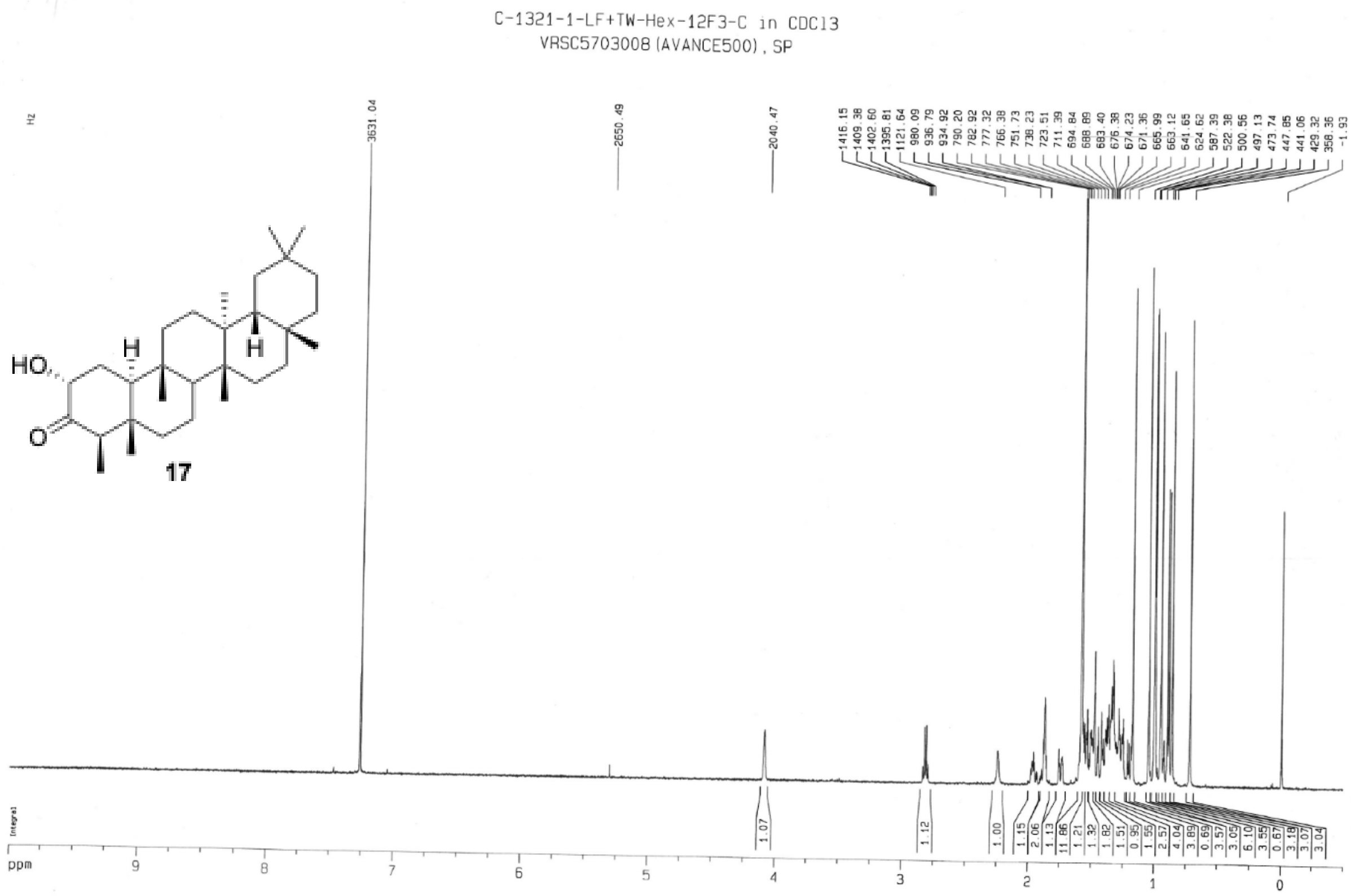

Figure S68. 125 MHz ^{13}C NMR spectrum of compound **17** in $CDCl_3$

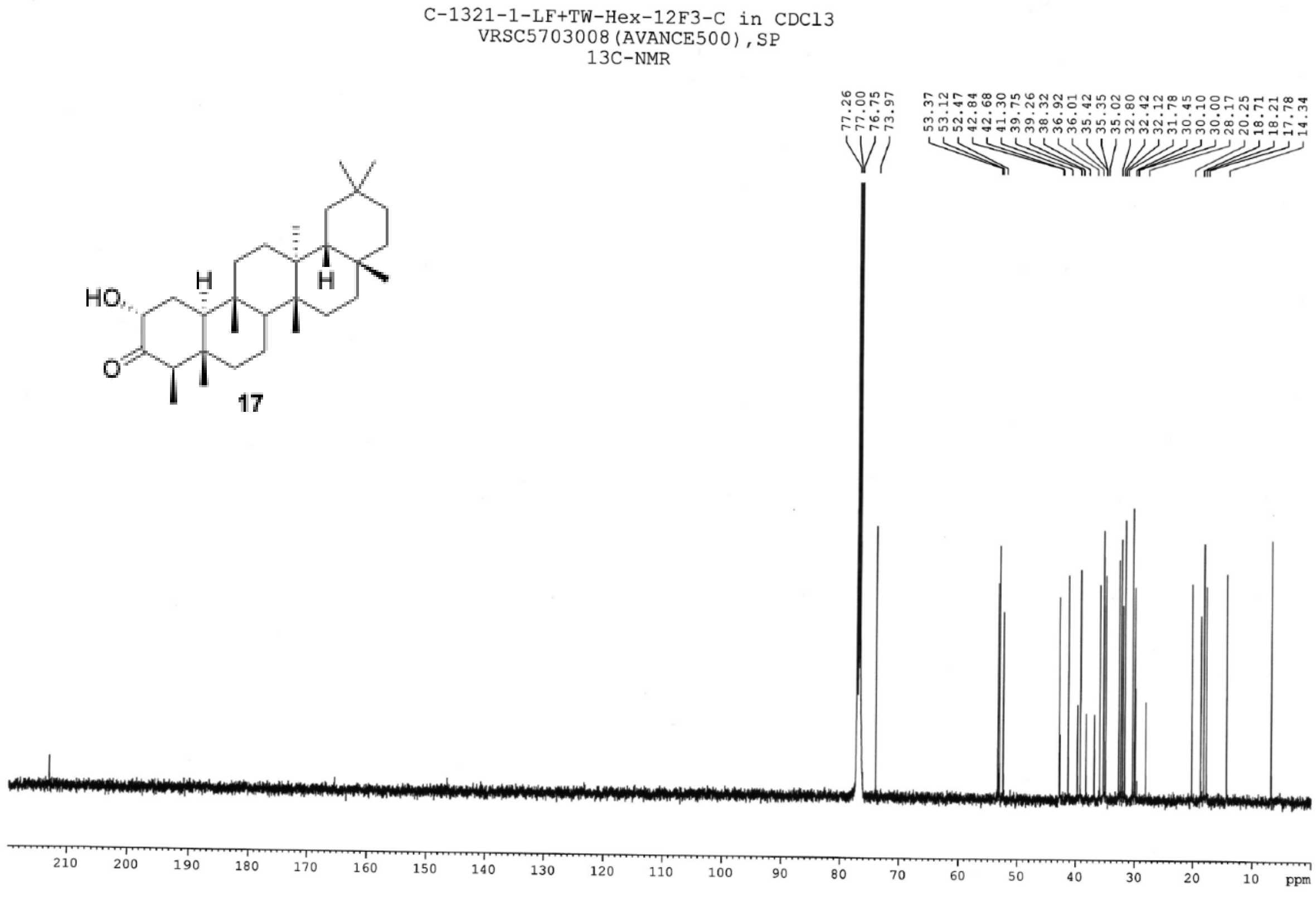

Figure S69. HRMS spectrum of compound **17**

Mass Spectrum List Report

Analysis Info

Analysis Name	VRSC570312001.d	Acquisition Date	3/12/2014 11:54:36 AM
Method	MKE_tune_wide_20130204.m	Operator	Administrator
Sample Name	C-1321-1-LF+TW-HEX-12F3-C<br>C-1321-1-LF+TW-HEX-12F3-C	Instrument	micrOTOF 72

Acquisition Parameter

Source Type	ESI	Ion Polarity	Positive	Set Corrector Fill	79 V
Scan Range	n/a	Capillary Exit	200.0 V	Set Pulsar Pull	406 V
Scan Begin	50 m/z	Hexapole RF	400.0 V	Set Pulsar Push	388 V
Scan End	3000 m/z	Skimmer 1	45.0 V	Set Reflector	1300 V
		Hexapole 1	25.0 V	Set Flight Tube	9000 V
				Set Detector TOF	1910 V

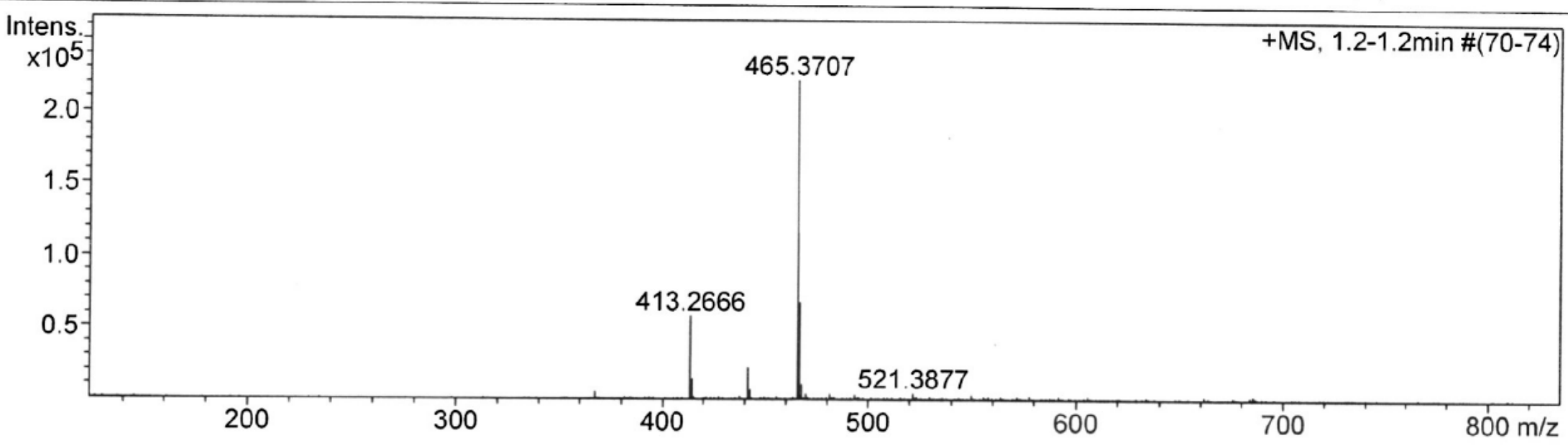

Figure S70. 500 MHz ^{1}H NMR spectrum of compound **18** in $CDCl_3$

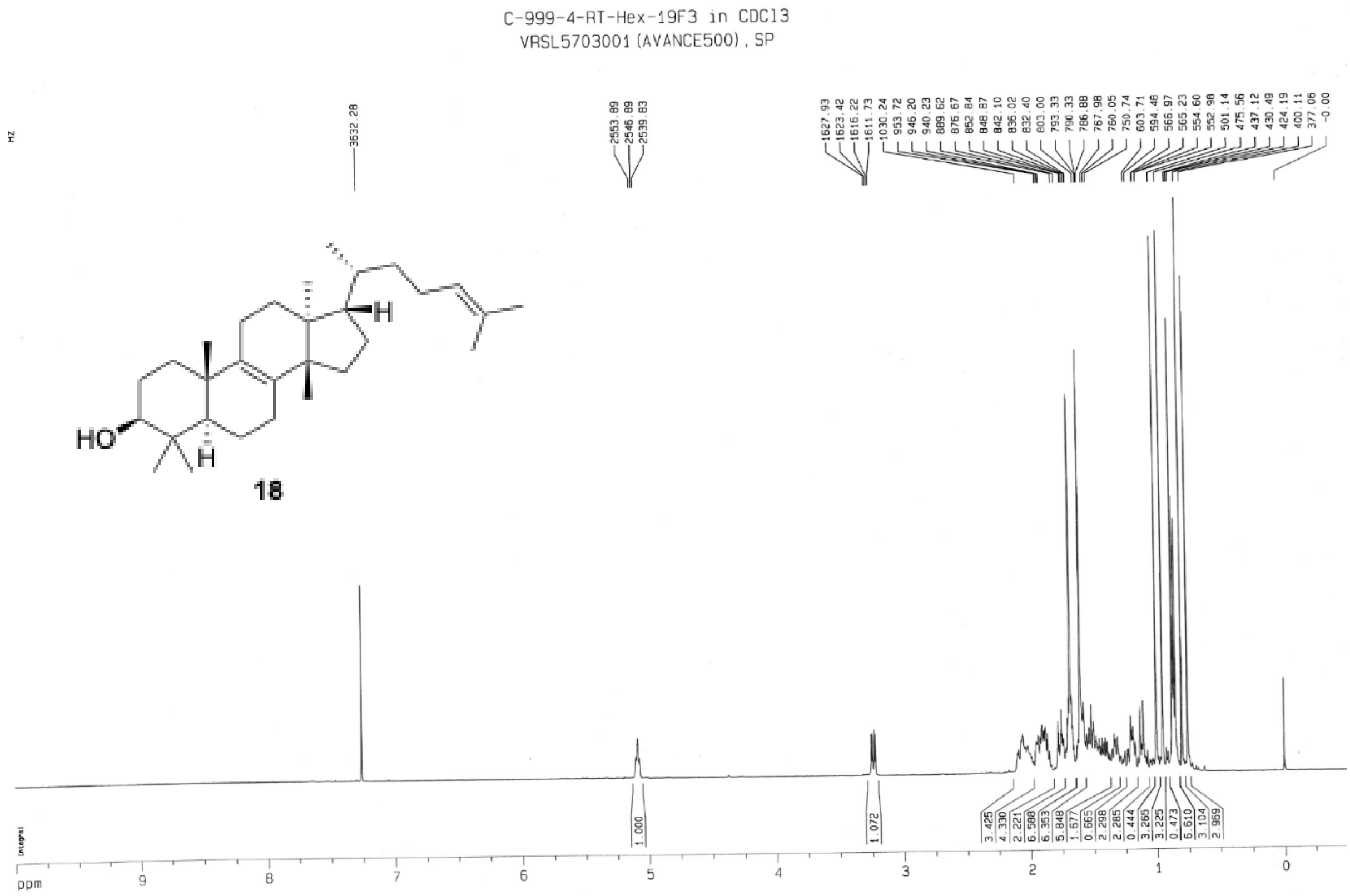

Figure S71. 125 MHz ^{13}C NMR spectrum of compound **18** in $CDCl_3$

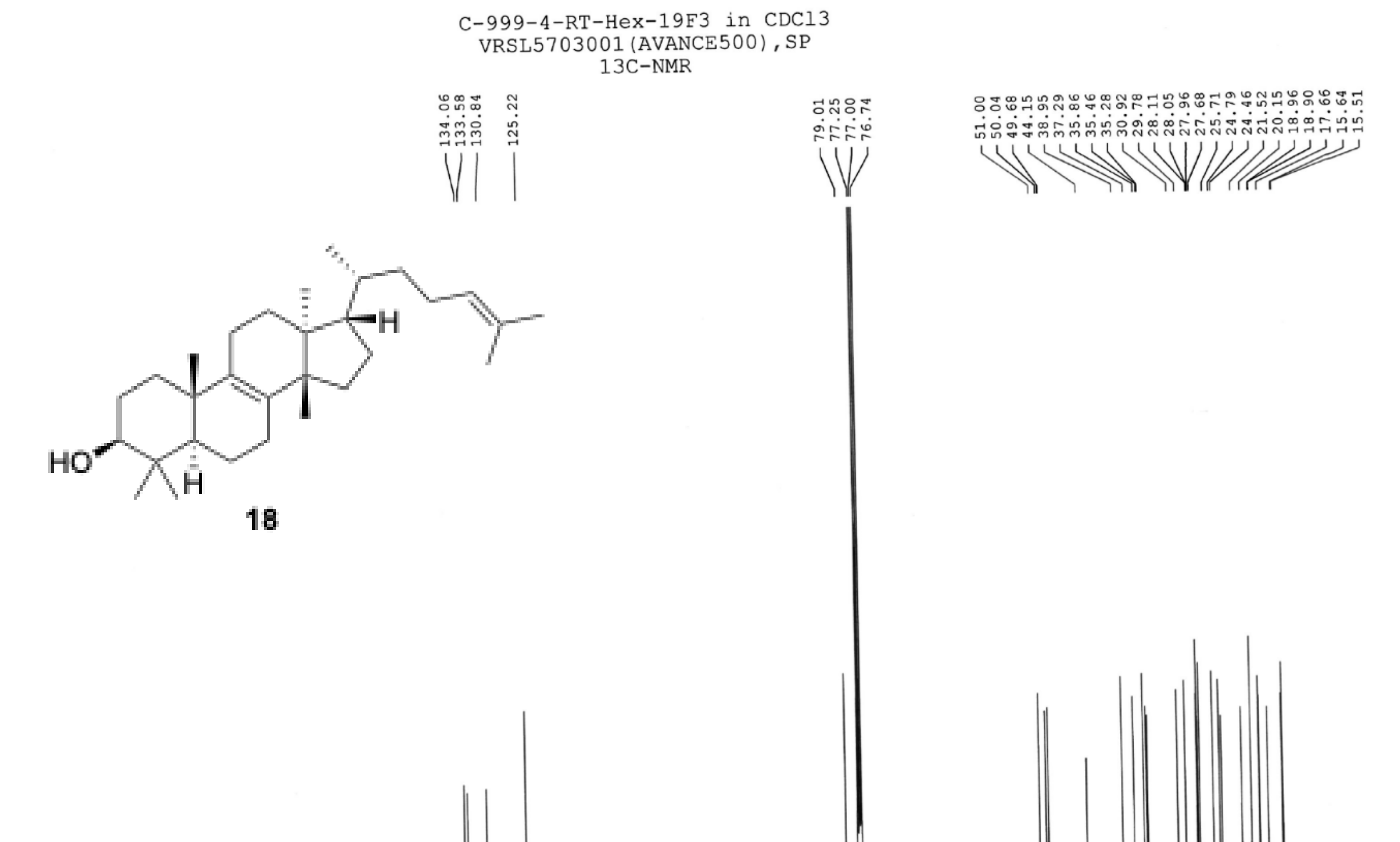

Figure S72. HRMS spectrum of compound **18**

Mass Spectrum List Report

Analysis Info

Analysis Name	CKSL570226001.d	Acquisition Date	2/26/2014 2:24:56 PM
Method	MKE_tune_wide_20130204.m	Operator	Administrator
Sample Name	C-999-4-RT-Hex-19F3	Instrument	micrOTOF 72
	C-999-4-RT-Hex-19F3		

Acquisition Parameter

				Set Corrector Fill	79 V
Source Type	ESI	Ion Polarity	Positive	Set Pulsar Pull	406 V
Scan Range	n/a	Capillary Exit	180.0 V	Set Pulsar Push	388 V
Scan Begin	50 m/z	Hexapole RF	400.0 V	Set Reflector	1300 V
Scan End	3000 m/z	Skimmer 1	45.0 V	Set Flight Tube	9000 V
		Hexapole 1	25.0 V	Set Detector TOF	1910 V

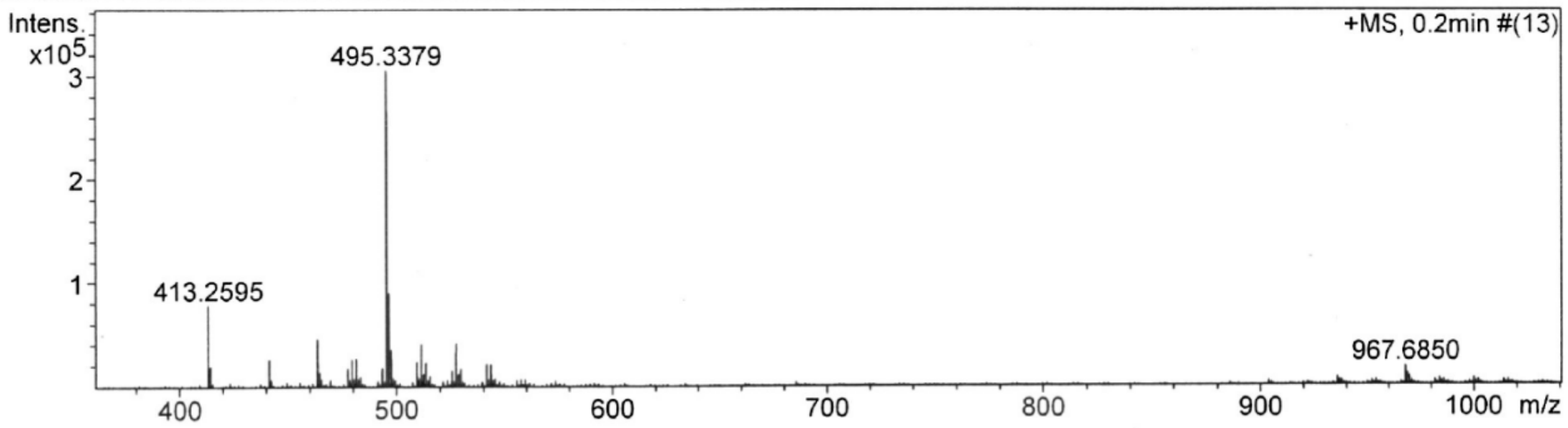

Table S1. Preliminary cytotoxicity screening of crude extracts

Extract	Cell lines (ED_{50})					
	P-388	KB	Col-2	MCF	Lu-1	ASK
Hexanes	>20	>20	>20	>20	>20	>20
EtOAc	<4	>20	>20	>20	>20	>20
MeOH	>20	>20	>20	>20	>20	>20

Results are expressed at ED_{50} value (µg/mL); ED_{50} <20 µg/mL is considered active. P-388 = murine lymphocytic leukemia, KB = human nasopharyngeal carcinoma, Col-2 = human colon cancer, MCF-7 = human breast cancer, Lu-1 = human lung cancer, ASK = rat glioma

Table S2. Preliminary anti-HIV-1 activity screening of crude extracts

Extract	Anti-syncytium				Anti-HIV-1 RT	
	IC_{50} (µg/mL)	EC_{50} (µg/mL)	SI	Activity	%Inhibition	Activity
Hexanes	136.03	34.08	3.99	A	51.92	M
EtOAc	182.07	116.80	1.56	A	–4.95	I
MeOH	95.54	64.29	1.49	A	88.48	VA

Syncytium inhibition assay: A = active, SI = Selective Index; RT assay: VA = very active (>70% inhibition), M = moderately active (>50% to 70% inhibition), I = inactive (<30%inhibition)

Figure S73. Dose-dependent anti-HIV-1 activity of isolated compounds.

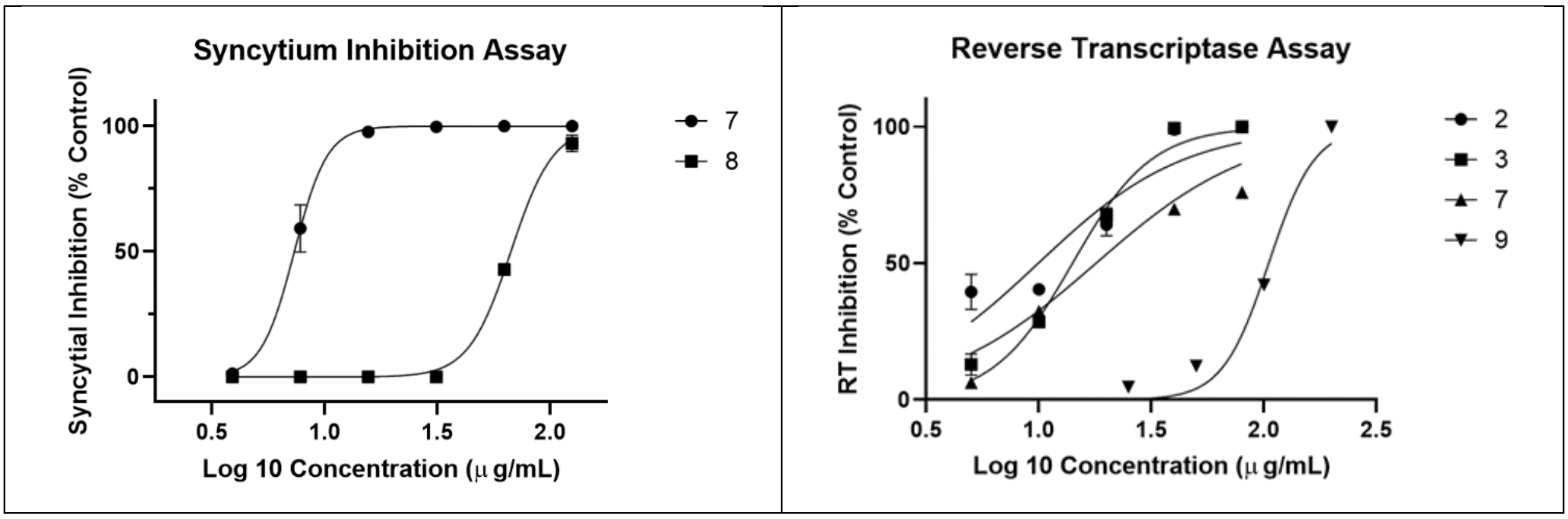

(A) Syncytium inhibition assay. Antiviral effects were quantified by the inhibition of syncytia formation. Compounds **7** and **8** exhibited EC_{50} values of 28.17 and 271.66 µM, respectively. Data shown are from a representative experiment performed in triplicate, expressed as the mean±SD.

(B) Reverse transcriptase (RT) assay. Compounds displaying high inhibitory activity at 200 µg/mL (**2**, **3**, **7**, and **9**) were selected for IC_{50} determination. Data represent the mean±SD of three independent experiments. All dose-response curves were generated using non-linear regression analysis in GraphPad Prism software.
